# Synthesis, molecular docking, assessment of biological and anti-diabetic properties of benzalacetophenone derivatives

**DOI:** 10.1038/s41598-025-96610-6

**Published:** 2025-04-23

**Authors:** Wesam S. Shehab, Nourhan Kh. R. Elhoseni, Wael M. Aboulthana, Mohamed G. Assy, Sahar M. Mousa, Gehan T. El-Bassyouni

**Affiliations:** 1 Department of Chemistry, Faculty of Science, Zagazig University, Zagazig, 44519 Egypt; 2 Biochemistry Department, National Research Centre, 33 El-Buhouth St., Dokki, Cairo, 12622 Egypt; 3 Inorganic Chemistry Dept, National Research Centre, 33 El-Buhouth St., Dokki, Cairo, 12622 Egypt; 4 Refractories, Ceramics and Building Materials Department, National Research Centre, 33 El-Buhouth St., Dokki, Cairo, 12622 Egypt

**Keywords:** Mn_3_O_4_ nano particle, Thiazine, Pyrazole, Pyrazoline, Diazapine, Biochemistry, Biological techniques, Drug discovery, Diseases, Molecular medicine, Chemistry, Nanoscience and technology

## Abstract

The current study compares the environmental impact of benzalacetophenone derivatives synthesized using conventional and nanomaterial-catalyzed techniques. Non-isolable thia-Michel adducts are produced when chalcone **1** reacts with thiourea thiolate anion; thiazine **2** is then obtained by cyclocondensation and dehydrogenation. Depending on the pH of the medium, hydrazine and target **1** can undergo pyrazole heterocyclization followed by N-acylation to produce compounds **3**,** 4**, and **5.** N-benzoylpyrazole derivative **6** was produced when compound 1 was allowed to condense with benzoylhydrazide. Hydroxylamine hydrochloride and chalcone 1 undergo cyclo-condensation, which releases H_2_O and yields an isoxazole derivative **7**. After cyclizing cyanoacetohydrazide via the α, β-unsaturated system and adding a cyclic imino moiety to the cyano functionality, product **8** is produced. Thioamide derivative **9** was made by the interaction of Target 1 with thiosemicarbazide. Acid caused 1 to cyclize with 2-aminothiophenol, forming thiazapine **10.** Michel’s addition initiated this process, which was followed by intramolecular cyclocondensation. Diazapines **11** and **12** were produced when compound 1 reacted with o-phenylenediamine and 3-nitro-o-phenylenediamine in a basic media. Reaction times and product yields were enhanced in several studies by using nanoparticles in place of conventional catalysts. Azoles and their derivatives are significant members of the organic chemical class due to their broad biological and pharmacological significance. All of the produced compounds exhibited strong antioxidant activity against DPPH and H_2_O_2_ radicals and noticeably higher activity against SOR and NO radicals when compared to regular ascorbic acid. Furthermore, the biological experiments that demonstrated the action of compounds **2**,** 3**, and **10** were corroborated by molecular docking analyses utilizing the MOE software, GacH as a maltose/maltodextrin-binding protein, and acarbose as the reference ligand.

**Supplementary Information:**

The online version contains supplementary material available at 10.1038/s41598-025-96610-6.

## Introduction

Concepts of diversity-oriented and adaptive multi-component processes have been a major source of inspiration for the synthetic scientific community in the past decade^1^. Certain chemicals that contain chalcones and the publication of their first creation was in 1899^2^. Active aromatic compounds known as chalcones are the progenitor of a wide range of bioorganic precursor molecules in medicinal science. Chalcones serve as useful bridges in the synthesis of several heterocyclic compounds, such as isoxazole^3,4^ and pyrazoline^5,6^, which have strong pharmacological effects^7,8^. Chalcones skeleton is found in a wide range of natural goods. It possesses beneficial bioactivities, including antioxidants that shield the body from chronic illnesses that could harm DNA and proteins and cause neurological, cardiovascular, and carcinogenic disorders^9^. They are very beneficial due to their anti-inflammatory qualities^10,11^. They are useful for a variety of purposes as xanthine oxidase inhibitors^12^, antihistaminic^13^, anticancer^14^, antimalarial^15^, antiviral^16^, antibacterial^17^, antioxidant^18^, antidiabetic^19^, and others. The widespread use of pyrazole and isoxazole heterocycles in the pharmaceutical and agricultural industries has drawn interest in the synthesis of their derivatives^20,21^, and gene inhibitory effects on histone deacetylase 3 and 8 (HDAC3 and HDAC8) and 20-hydroxy eicosatetraenoic acid (20-HETE) synthase^22–24^. Additionally, research backs up the usefulness of benzothiazepine and benzodiazepine compounds as anti-inflammatory medications^25,26^. There is evidence linking some benzothiazepine chemicals to possible calcium channel blocker action^27^. Research has demonstrated that benzodiazepines can reduce the activities of p53-mouse double minute 2 (p53-MDM2) and centrin-specific protease 1 (SENP1)^28^. Heterocyclic compounds containing an aryl sulfonate group are well known for their potent antibacterial properties^29^, and they also show promise in treating conditions such as HIV-1^30–32^. In recent years, metal nanoparticles, especially those supported by functionalized magnetic nanoparticles and superparamagnetic metal Nanoparticles, have attracted significant attention due to their potential applications in chemical. These innovations have allowed researchers to use Nano-catalysts as durable and eco-friendly alternatives in organic reactions^33–35^. However, interactions with metal ions can negatively affect the pharmacokinetics, bioavailability, and solubility of the resulting organic compounds, altering the mechanism of action of fungicidal and bactericidal drugs^36^.

## Results and discussion

### Characterization of Mn_3_O_4_ nanoparticle

#### XRD analysis

**Fig. 1:**
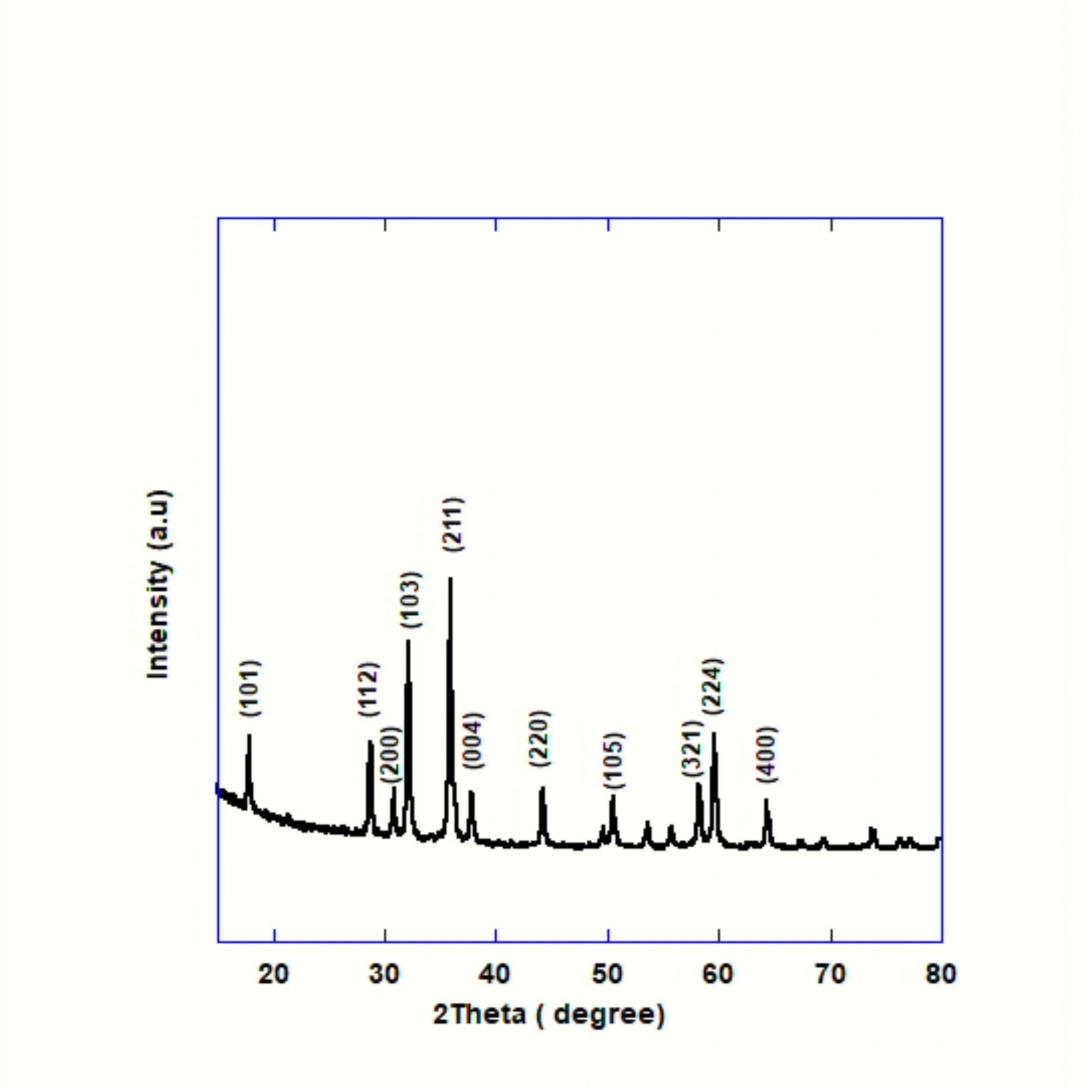
XRD pattern of Mn_3_O_4_-NPs.

The hausmannite structure (Mn_3_O_4_, the stable phase of manganese oxide) exhibits diffraction peaks in the powdered crystalline sample’s XRD pattern (Fig. 1). Comparing the measured diffraction peak positions to the standard value, it is determined that they are consistent with [(JCPDS 24–0734)], which is related with the tetragonal single phase of (Mn_3_O_4_). The tetragonal unit cell’s lattice characteristics, which are in line with earlier discoveries, were determined to be (a = b = 5.76 and c = 9.46)^37^. The XRD result showed no more impurity peaks, suggesting that no other manganese oxide formulations were found.

#### Morphological and elemental analysis

**Fig. 2:**
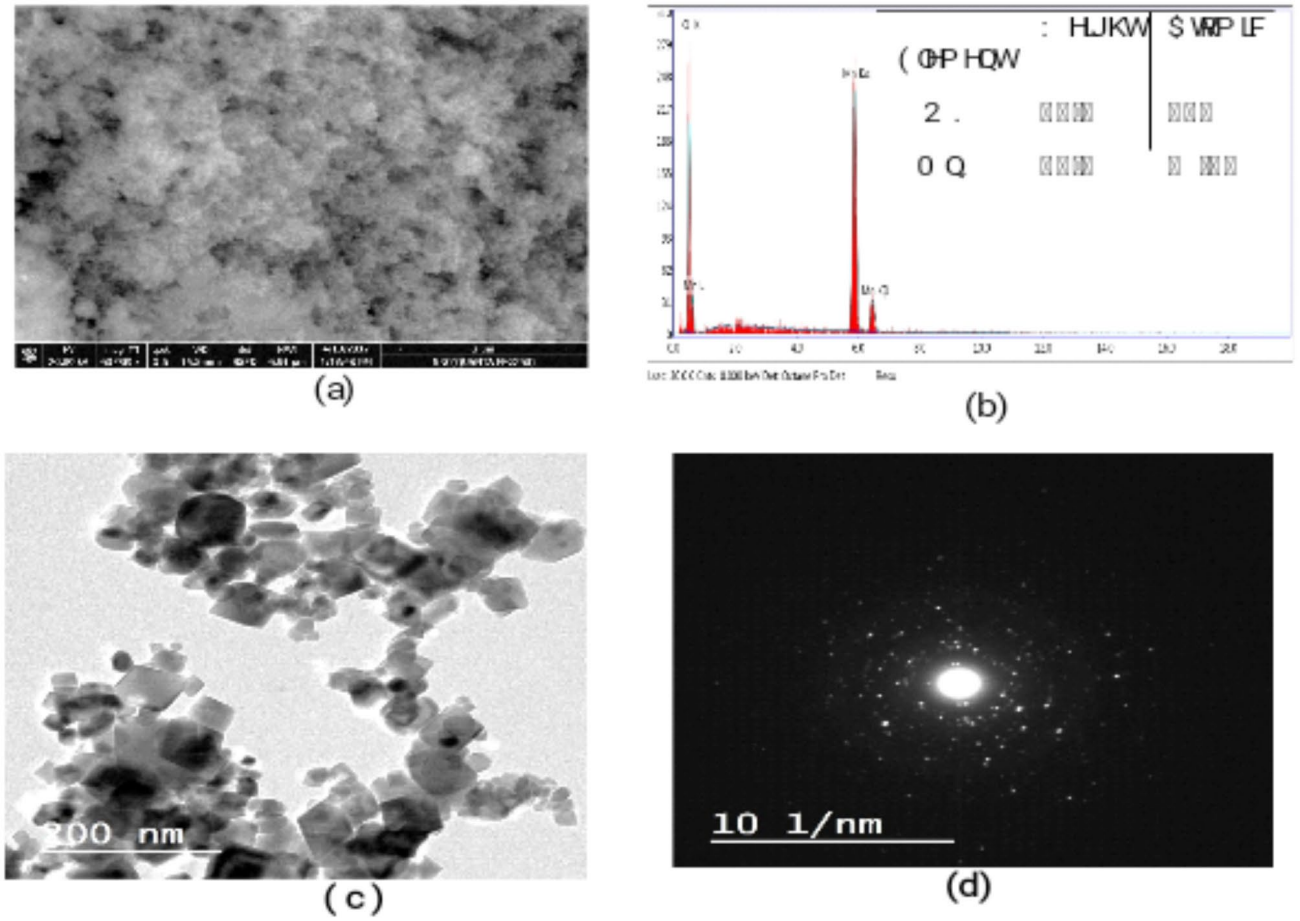
FE**-**SEM image (**a**) EDX spectrum (**b**) and HR-TEM image (**c**) diffraction pattern (SAED) of Mn_3_O_4_-NPs (**d**).

The size, shape, and crystallinity of Mn_3_O_4_ particles have all been investigated using field emission scanning microscopy (FE-SEM) and high-resolution transmission electron microscopy (HR-TEM). Figure 2a shows the (Mn_3_O_4_-NP) FE-SEM image. It was found that the particles were mostly agglomerated yet consistent in size, with either a spherical or cubic shape^38^. Only the elements of Mn and O were found in the EDX results as presented in Fig. 2b, indicating the remarkably pure nature of the synthesized sample. A typical HR-TEM image of the manganese oxide nanostructure (Mn_3_O_4_) is displayed in Fig. 2c^39^. The sample was mainly made up of cubic particles with a limited number of spherical structures, as evidenced by the particle size distribution, which was found to be between 15 and 70 nm. This observation agrees with SEM results^40^. Mn_3_O_4_ nanoparticles are polycrystalline, as seen by the selected area electron diffraction (SAED) pattern in Fig. 2d^41^.

### Chemistry

**Fig. 3:**
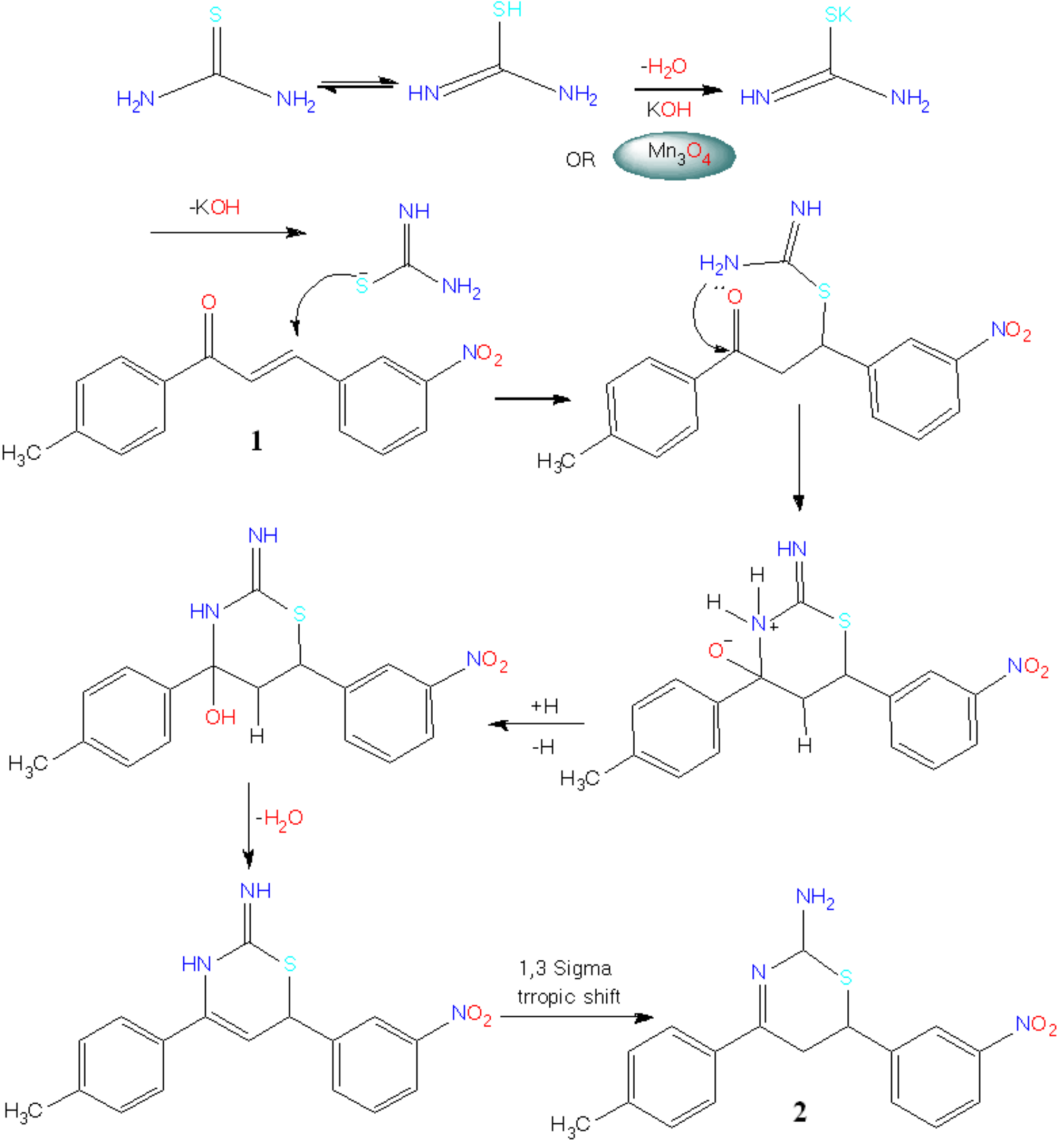
Mechanism of Mn_3_O_4_-NPs catalysis.

After employing Mn_3_O_4_ nanoparticles to carry out the reaction, an environmentally friendly synthesis of 2-aminothiazine **2** was achieved. Enolic thiourea’s reaction with base-induced hetero-cyclization of benzalacetophenone **1**^42^ produced **1**, 3-thiazine. Via Michael addition initiated the reaction, which was subsequently followed by intramolecular cyclization resulting in the departure of H_2_O through nucleophilic attack of NH_2_ on the electrophilic ketonic carbonyl carbon, yielding thiazine **2** as shown in (Fig. 3) “the optimizing table includes this reaction, as presented in Table 1”.

Infrared spectra of Thiazine 2 showed a C-S stretching band at 1176.11 cm^− 1^ and absorption bands at 3335.69 and 1658.25, which corresponded to the NH_2_ and C = N group. The ^1^H-NMR spectra revealed three distinct signals: CH_3_ at δ 2.50 ppm, CH at δ 3.47–3.49 ppm, CH_2_ (multiplet non-equivalent proton) at δ 4.55–4.64 ppm and CH at δ 5.31 ppm. In addition to the NH_2_ proton-related peak signal at δ 8.76 ppm.

**Table 1:** Demonstrates the reaction conditions optimization for the preparation of organic derivative products.

Compound	Solvent	Catalyst (base)	Time (h)	Yield (%)
**1**	EtOH	KOH	2	95
		Mn_3_O_4_	1/2	99
**2**	EtOH	KOH	5	47
		Mn_3_O_4_	2	65
**8**	EtOH	–	6	No reaction
		Mn_3_O_4_	2	45
**9**	AcOH	HCl	6	62
		Mn_3_O_4_	2	70
**10**	AcOH	HCl	6	44
		Mn_3_O_4_	2	60
**11**	EtOH	KOH	6	50
		Mn_3_O_4_	2	66
**12**	EtOH	KOH	6	55
		Mn_3_O_4_	2	70

**Fig. 4:**
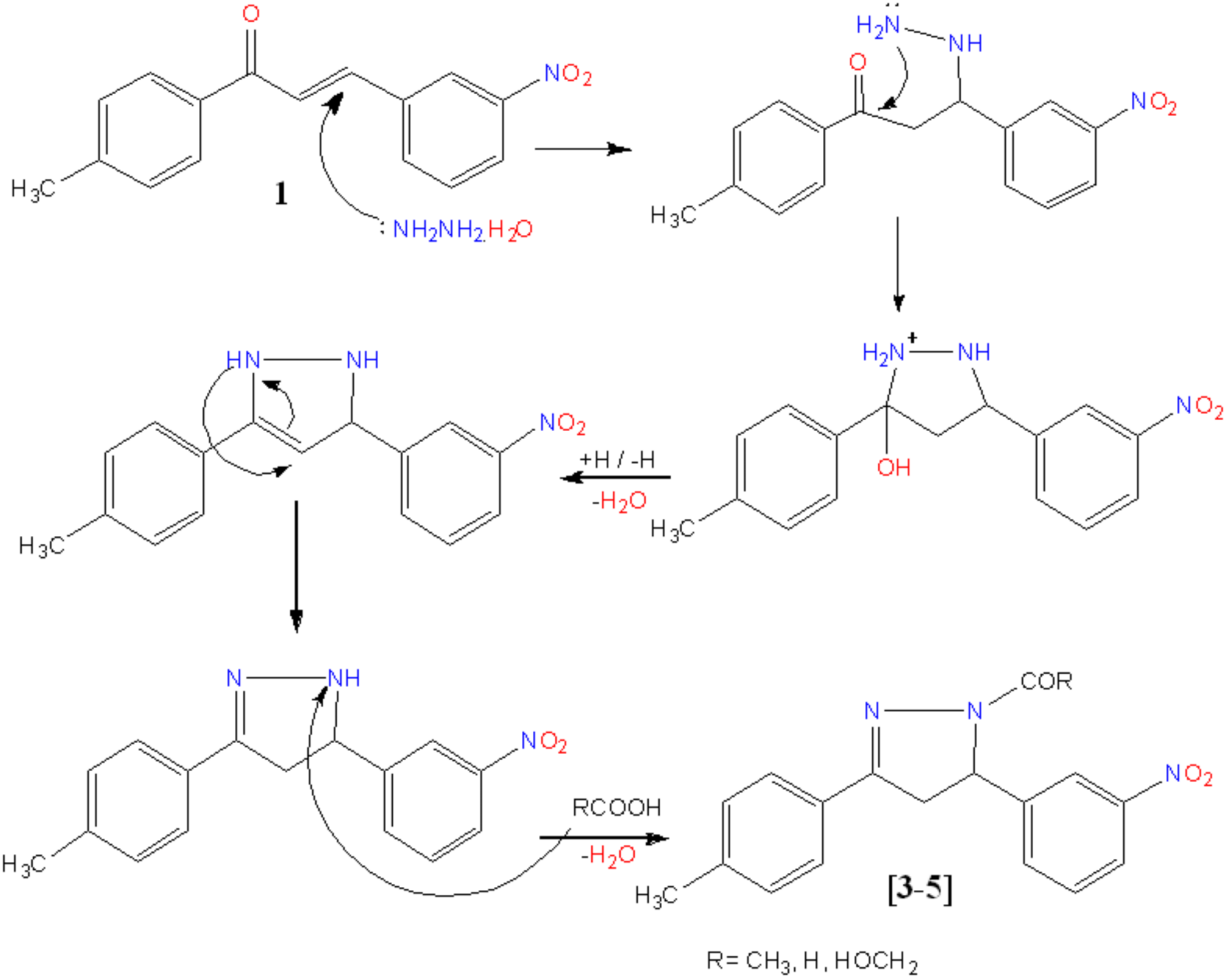
Mechanism of acylated pyrazole derivatives.

Refluxing of hydrazine and target **1** in acetic acid increases the electrophilic nature of C = O resulting in pyrazole cyclization followed by acylation producing N-acylated pyrazole derivatives **3** (Fig. 4). Its IR spectrum exposed absorption bands at 1650.39 cm^− 1^ for the C = O group Additionally, the ^1^H-NMR spectra showed singlet signals at δ 2.50 ppm (CH_3_), multiplet signals at δ 3.83–3.93 ppm (CH_2_), and multiplet signals at δ 5.67–5.72 ppm (CH). ^13^C NMR spectra stated at δ 167.62 ppm for C = O. Benzalacetophenone derivative **1** undergoes a reaction with NH_2_NH_2_ in boiling formic acid Generated N-formylpyrazole **4 ** (Fig. 4). In compound **4**, for instance, the IR spectrum displayed an absorption band for the aldehyde’s C = O at 1709.04 cm^− 1^. A singlet corresponding to CH_3_ at δ 2.50 ppm, CH_2_ signals at δ 3.89–3.99 ppm, CH signals at δ 5.68–5.73 ppm, and CH signals for aldehyde at δ 8.92 ppm were among the ^1^H NMR evidence that supported the structure. The appearance signal in the low field area of δ 156.20–159.80 ppm in the ^13^C NMR spectra was attributed to the carbonyl group. The reaction of chalcone **1** and hydrazine in the presence of the equivalent amount of glycolic acid resulted in acyl pyrazole **5 ** (Fig. 4). The IR spectrum of compound **5** exhibited a broad band at 3224.26–3463.16 cm^− 1^ which suggested the existence of the OH group, and the characteristic bands 1743.87 and 1643.58 cm^− 1^ signifying the presence of C= O and –C= N groups, respectively. The ^1^H NMR spectra of compound **5** showed CH and CH_2_ signals of the pyrazoline ring at δ 3.83–4.84 ppm and 5.69–5.75 ppm respectively. In addition to the peak signals at δ 9.51 ppm due to OH protons and CH_2_ protons of the acylated group at δ 5.44 ppm. At 169.70 ppm the carbon signal of *sp*^2^ carbonyl carbon was exposed.

**Fig. 5:**
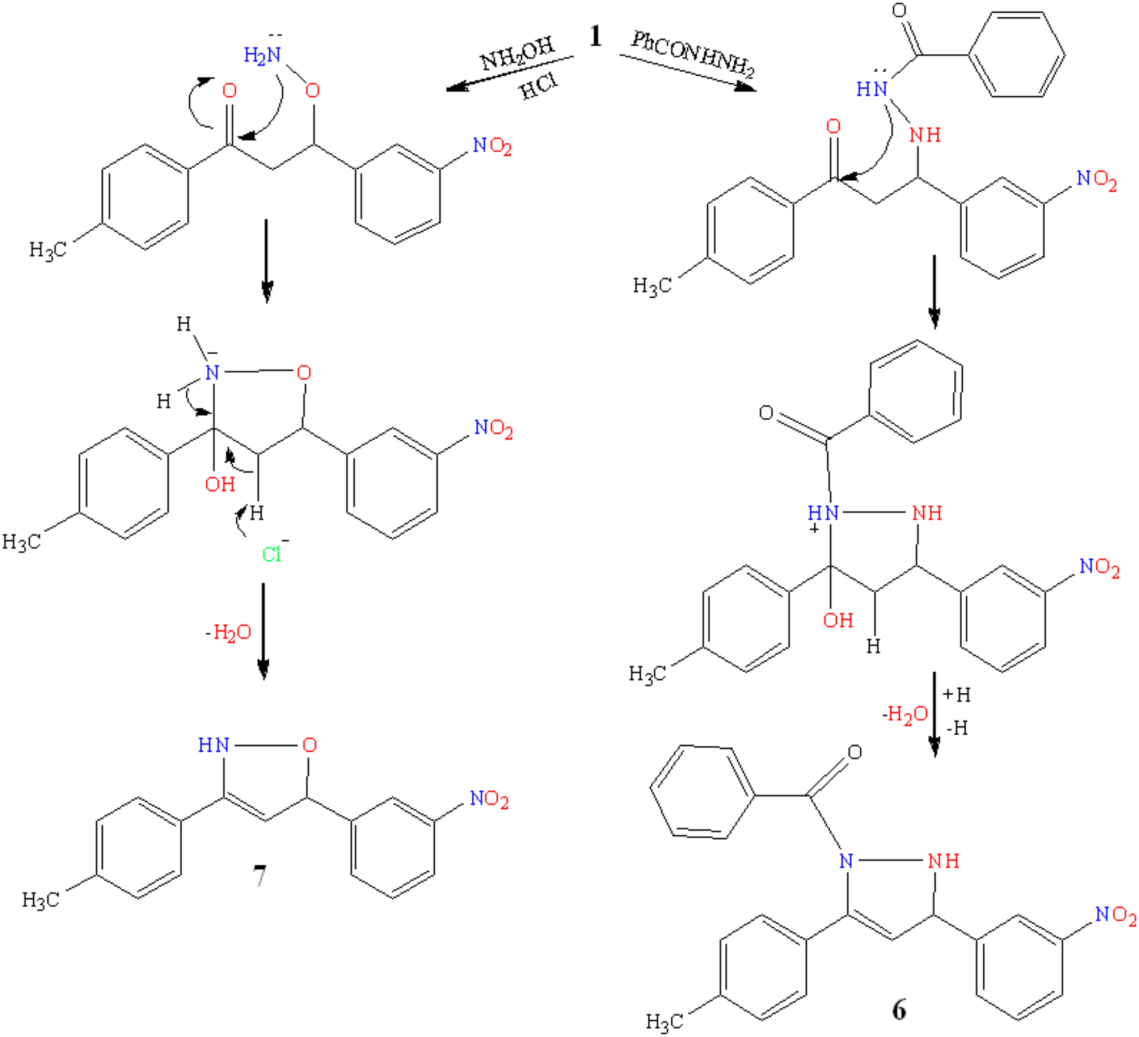
Synthesis of azole derivatives.

**Fig. 6:**
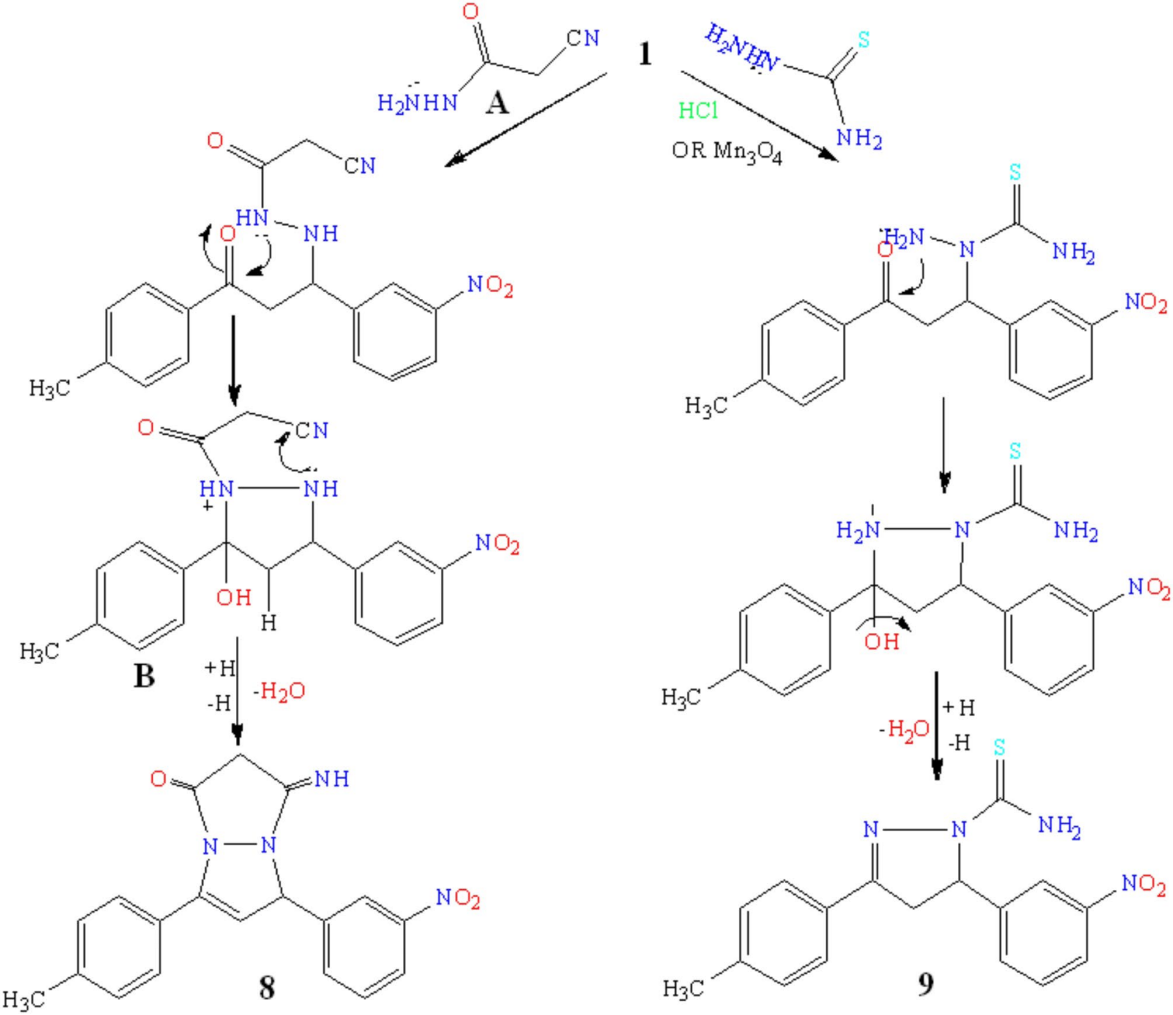
Synthesis of pyrazole derivatives.

The condensation reaction between compound **1** and benzoylhydrazide lead to N-benzoylpyrazole derivative **6**. Compound **6** exhibited IR spectrum at 3156.80 cm^− 1^ and 1667.79 cm^− 1^ for (NH), (C = O) respectively, whereas ^1^H-NMR spectra discovered the appearance of CH protons of pyrazoline ring at δ 2.35–2.36 ppm and 3.56-3-57 ppm and -NH signals at δ 10.49 ppm presented in (Fig. 5). An increase in the quantity of aromatic carbon signals was perceived in the ^13^C NMR spectrum of **6** at δ 127.47-132.56 ppm in addition to a peak at δ 165.85 ppm for C = O. Hydroxylamine hydrochloride and benzalacetophenone **1** perform a cyclo-condensation reaction, resulting in the elimination of H_2_O to produce isoxazole derivative **7**. Its infrared spectra revealed absorption bands for the C = C group at 1662.06 cm^− 1^ and for NH at 3191.42 cm^− 1^. Moreover, singlet signals at δ 11.70 ppm in its ^1^H-NMR spectrum were detected and assigned to the NH group (Fig. 5). Refluxing cyanoacetohydrazide and chalcone **1** in absolute ethanol resulted in complete recovery of the unchanged reactant. Target **1** was subjected to react with hydrazide **(A)** in the presence of Nano-catalyst via the formation of non-isolable canoacetopyrazole derivatives **(B)** that undergo intramolecular pyrazole cyclization via the addition of cyclic imino function to cyano group to form pyrazole **8** (Fig. 6). IR spectrum **8** contained C= N and C= O peaks 1604.14 and 1677.59 cm^− 1^, respectively. Compound **8** ^1^H-NMR spectrum showed two signals at 8.68 ppm, which were attributed to the presence of NH groups.

The reaction of equivalent amounts of thiosemicarbazide and benzalacetophenone **1** led to pyrazole cyclization to furnish pyrazole **9** when the reaction catalyzed by HCl increased the reactivity of carbonyl carbon of α, β unsaturated system while when the reaction was conducted on the absence of acidic medium the product was recovered with completely unchanged due to the un reactivity of carbonyl carbon by hyperconjugation of CH_3_. A good yield of **9** when catalyzed by nanoparticle Mn_3_O_4_ and acetic acid was achieved (Fig. 6). IR spectra of carbothioamide **9** showed that the C = N group had absorption bands at 1642.46 cm^− 1^, the C= S group had a stretching band at 1284.32 cm^− 1^, and the NH_2_ group had an absorption band at 3259.26 cm^− 1^.

The carbothioamide **9**’s ^1^H-NMR spectra presented that the pyrazoline ring’s CH_2_ multiplet signals at 3.31–3.49 ppm and CH signals at 5.57–5.59 ppm, and (NH) group appeared at 8.73 ppm. A signal was observed at δ 181.04 ppm for C = S in its ^13^C-NMR analysis (Fig. 6.

**Fig. 7:**
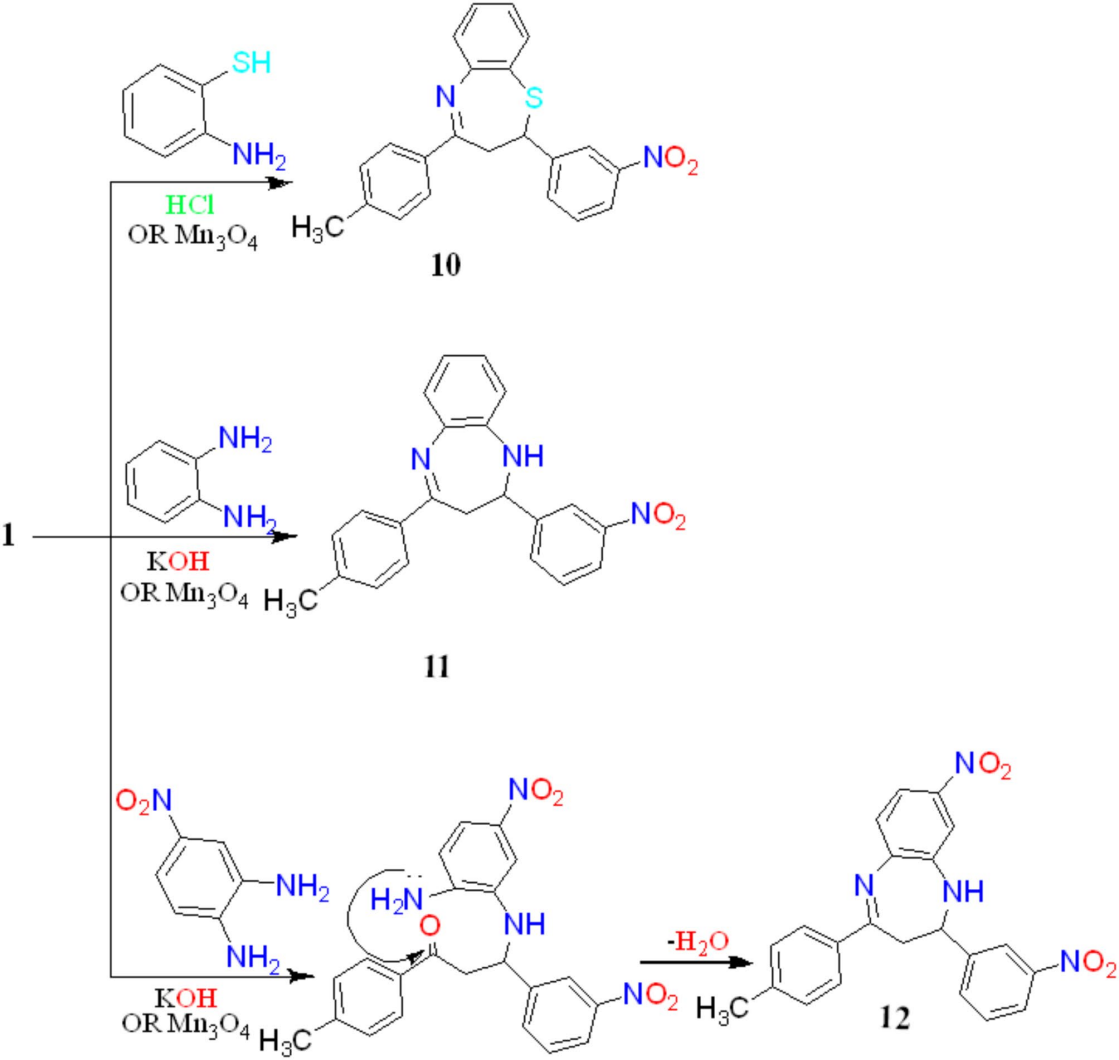
Synthesis of benzodiazepine and benzothiazepine derivatives.

Nano-catalytic process of 2-amino thiophenol with object **1** proceeded in a good yield and shortened the time of the traditional reaction to form **10**. Acid-mediated reaction of 2-aminothiophenol with benzalacetophenone **1** resulted in heterocyclization affording **10**. The reaction may be started by the addition of (SH) function to the polarized π band through 1,4 addition followed by intramolecular cyclodehydration via the attack of nucleophilic (NH_2_) to the electrophilic carbonyl carbon followed by dehydrogenation. Compound **10** showed a strong band for the C =N group at 1673.94 cm^− 1^ in The IR spectrum of **10** and the presence of a multiplet signal at δ 3.43–3.45 ppm represents the CH_2_ proton of thiazepine in the ^1^H-NMR spectrum. ^13^C-NMR spectra of compound **10** showed a signal at δ 181.04 ppm related to C= N (Fig. 7). *o*-phen-ethylenediamine and 3-nitro-*o*-phenylenediamine Nano catalytic procedure with purpose **1** produced a good yield and shortened the time required for the conventional reaction to generate **11** and **12**. Base-induced cyclocondensation of *o*-phenylenediamine and 3-nitro-*o*-phenylenediamine lead to diazapine derivatives **11** and **12**. The addition of an amino function to the polarized π band by 1,4 additions may initiate the reaction. This can be followed by intramolecular cyclodehydration, which occurs when nucleophilic nitrogen attacks the electrophilic carbonyl and then dehydrogenates. Conversely, the derivatives **11** and **12** exhibited (NH) group peaks at 3357.31 cm^− 1^ and 3352.16 cm^− 1^, along with a prominent C= N band at 1658.07 cm^− 1^ and 1659.81 cm^− 1^, respectively. The ^1^H-NMR spectrum displayed two singlet signals for **11** and **12** compounds at δ 2.50 ppm and 3.32–3.46 ppm for CH_3_ and CH_2_ correspondingly (Fig. 7 ).

### Molecular docking

During the development of novel medications, molecular docking studies are widely used to forecast how ligands and proteins will interact. The binding affinity is computed and the amino acid interactions that contribute to it are visualized to achieve this. By determining how well-isolated chemicals attach to proteins involved in glucose metabolism, docking makes it possible to anticipate anti-diabetic performance. Thus, in our work, we performed molecular docking research for novel synthesized azoles and azines to correspond with co-crystallized acarbose as a reference ligand isolated from GacH as a maltose/maltodextrin-binding protein (PDB code: 3JZJ)^43^. The pseudodisaccharide carnosine, which is the core structure of acarbose-like secondary metabolites, is mainly responsible for the inhibitory activity. It is composed of an unsaturated C7 cyclitol that is attached to 4-amino-4,6-dideoxyglucose via an imino bridge. Our candidates had extremely positive binding scores (**2**= − 7.068, **3**= − 6.735, and **10**= − 7.422 kcal/mol) contrasted to acarbose binding score (-12.318 kcal/mol), the conclusion drawn from these results was that these compounds were stabilized within the binding pocket of GacH as a maltose/malto-dextrin-binding protein. Additionally, acarbose was stabilized inside the protein binding pocket by bound eleven H-bonds GLU-32, ASP-133, GLU-83, ASP-180, GLU-83, ARG-81, TRP-290, ARG-358, ARG-358, TRP-254 and TYR-182 at 2.75, 2.89, 2.75, 2.84, 2.73, 2.93, 3.02, 2.92, 2.93, 3.96 and 4.16 Å, respectively. Table 2 demonstrates how the binding of essential amino acids stabilized candidates **2**,** 3**, and **10** in the binding pocket. Table 3 shows the produced ligands’ 2D and 3D receptor interactions with the biggest pocket (3JZJ) protein.

**Table 2:** The docked acarbose reference standard within the binding pocket of GacH binding protein is compared to the binding scores, RMSD values, receptor contacts, distances, and energy of twelve candidates (**2**, **3**, and **10**).

Ligand	Docking Score	RMSD	Receptor interaction	Distance Å	E (Kcal/mol)
**2**	− 7.068	1.099	ASP-133/H-donor	2.82	− 5.8
			ARG-358/H-acceptor	3.35	− 0.8
			GLU-83/Ionic	3.92	− 0.7
			ASP-133/Ionic	3.70	− 1.2
			ASP-133/Ionic	3.70	− 1.2
			ASP-133/Ionic	2.82	− 5.8
			ASP-133/Ionic	3.84	− 0.8
**3**	− 6.735	2.212	ARG-81/H-acceptor	3.25	− 0.5
			PHE 59/H-acceptor	3.04	− 1.4
			TRP-254/H-pi	3.88	− 0.8
**10**	− 7.422	1.045	PHE-59/H-acceptor	3.14	− 0.8
			TRP-234/H-pi	3.80	− 1.2
			ASN-29/pi-H	4.33	− 0.7
Acarbose (docked)	− 12.318	1.564	GLU-32/H-donor	2.75	− 4.5
			ASP-133/H-donor	2.89	− 3.0
			GLU-83/H-donor	2.75	− 4.1
			ASP-180/H-donor	2.84	− 3.3
			GLU-83/H-donor	2.73	− 2.7
			ARG-81/H-acceptor	2.93	− 3.9
			TRP-290/H-acceptor	3.02	− 0.9
			ARG-358/H-acceptor	2.92	− 3.1
			ARG-358/H-acceptor	2.93	− 2.7
			TRP-254/H-pi	3.96	− 0.8
			TYR-182/H-pi	4.16	− 1.0

**Table 3:** Displays the receptor interaction and position of the most promising possibilities (**2**, **3**, and **10**) within the binding protein pocket about the reference standard of docked acarbose.

Molecule	2D receptor interaction	3D receptor interaction	3D receptor positioning
**2**			
**3**			
**10**			
Acarbose (docked)			

### In vitro biological activity

#### Antioxidant activity

Consumption of exogenous antioxidants is considered the main strategy for preventing and treating various chronic diseases such as cancer, atherosclerosis, and rheumatoid arthritis that are induced by excessive lipid oxidation and inflammation^44^. TAC and IRP levels were measured at equal doses (1000 µg/mL) to evaluate the antioxidant activity of the various synthetic compounds. As shown in Table 4, TAC and IRP levels were greater in compounds **2** and **10** than in the other examined compounds. One possible explanation for this could be the existence of easily oxidized free amino groups. This is because oxidants can also attack these compounds because the nitrogen atom only has one pair of electrons^45^. Furthermore, the primary source of these compounds’ antioxidant activity is their capacity to transfer a hydrogen atom to alkyl peroxyl radicals without participating in the chain reaction that propagates peroxidative radicals through reactions with hydroperoxides or substrates. The Bond Dissociation Enthalpy, which is dependent on the substituents on the aromatic ring, is inversely correlated with these characteristics. Alkyl and methoxy groups at the ortho and para positions, on the other hand, donate electrons to the phenoxyl radical, lowering the Bond Dissociation Enthalpy and increasing antioxidant activity^46^. To create more potent antioxidants that may also show enhanced biological activity, the substitution of sulfur for oxygen in the scaffold of cyclic compounds has been explored^47^.

**Table 4:** Antioxidant and scavenging activities at equal concentrations (1000 µg/mL) of the different synthetic compounds.

Sample	Antioxidant activity		Scavenging activity	
	TAC (mg Gallic acid/g)	IRP (µg/mL)	DPPH	ABTS
			Inhibition (%)	
1	21.63 ± 0.04	16.48 ± 0.04	14.33 ± 0.04	19.48 ± 0.04
2	**77.46 ± 0.38**	**72.31 ± 0.38**	**62.88 ± 0.33**	**68.03 ± 0.33**
3	52.16 ± 0.34	47.01 ± 0.34	40.88 ± 0.29	46.03 ± 0.29
4	21.97 ± 0.04	16.82 ± 0.04	14.62 ± 0.04	19.77 ± 0.04
5	17.30 ± 0.04	12.15 ± 0.04	10.57 ± 0.03	15.72 ± 0.03
6	45.36 ± 0.29	40.21 ± 0.29	34.96 ± 0.26	40.11 ± 0.26
7	22.15 ± 0.05	17.00 ± 0.05	14.78 ± 0.04	19.93 ± 0.04
8	38.21 ± 0.29	33.06 ± 0.29	28.75 ± 0.26	33.90 ± 0.26
9	46.49 ± 0.30	41.34 ± 0.30	35.95 ± 0.26	41.10 ± 0.26
10	**78.12 ± 0.39**	**72.97 ± 0.39**	**63.45 ± 0.34**	**68.60 ± 0.34**
11	68.80 ± 0.33	63.65 ± 0.33	55.35 ± 0.29	60.50 ± 0.29
12	69.74 ± 0.34	64.59 ± 0.34	56.16 ± 0.30	61.31 ± 0.30
Ascorbic acid	86.40 ± 0.08	79.43 ± 0.10	68.35 ± 0.04	73.50 ± 0.04

The data was computed using three replicates and displayed as mean ± standard error (SE). The sample with the highest activity is indicated by an bold.

#### Scavenging activity

The radical scavenging activity of the different synthetic compounds is the main role of antioxidants, which would be responsible for inhibiting hydrogen atom transfer and electron migration^45^. It was evaluated by calculating their inhibitory effect against DPPH and ABTS radicals at equal concentrations (1000 µg/mL). Furthermore, the IC_50_ values of DPPH and ABTS scavenging activities were calculated. It was presented that the compounds with greater antioxidant activity had lower IC_50_ values.

Since the ABTS assay is involved in the electron transfer pathway (ABTS to ABTS) and the DPPH radical is exclusively involved in hydrogen (H^+^) transfer (DPPH to DPPH-H), it was discovered that the ABTS assay is more sensitive than the DPPH assay^48^. As shown in Table 4 and based on the data from the TAC and IRP mentioned above, it was observed that compounds **2** and **10** exhibited higher inhibition percentages (%) against DPPH 62.88 ± 0.33 and 63.45 ± 0.34%, respectively) and ABTS radicals 68.03 ± 0.33 and 68.60 ± 0.34%, respectively) with lower IC_50_ values against both DPPH 5.17 ± 0.04 and 5.12 ± 0.04 mg/mL, respectively) and ABTS radicals 4.66 ± 0.04 and 4.62 ± 0.04 mg/mL, respectively) (Table 6). The compounds exhibited a linear rise in DPPH and ABTS radical scavenging capabilities as their concentrations increased, potentially due to the elevation of TAC and IRP^49^. In the current investigation, at equivalent doses, ascorbic acid scavenges more DPPH and ABTS radicals than the investigated substances. Removing water-soluble free radicals is one of its representative antioxidant qualities. It becomes an ascorbate radical when the ascorbate radical’s electron provides a lipid radical to stop the oxidative chain reaction^50^.

#### Anti-diabetic activity

Elevated glucose levels are a hallmark of diabetes mellitus (DM), a chronic metabolic illness^51^. By evaluating the inhibitory effect of various synthetic compounds on α-amylase and α-glucosidase enzymes and contrasting it with the efficacy of acarbose, a conventional medicine, the anti-diabetic activity of these compounds was assessed. The α-amylase and α-glucosidase enzymes control blood glucose levels. Carbs are broken down into disaccharides by α-amylase, and then into monosaccharides by α-glucosidase. One of the best treatment approaches is thought to be preventing these enzymes from functioning to control hyperglycemia^52^. Comparing compounds **2** and **10** to acarbose’s effectiveness against α-amylase (70.05 ± 0.15%) and α-glucosidase activity (59.90 ± 0.15%) at equal concentrations, the former showed the highest inhibitory effect on both enzymes (59.15 ± 0.29 and 59.65 ± 0.29%, respectively) and α-glucosidase activity (49.00 ± 0.29 and 49.50 ± 0.29%, respectively) (Table 5). Concerning the IC_50_ values, the lowest values were noticed with these compounds against both α-amylase and α-glucosidase as shown in Table 6. In vitro, inhibition of these enzymes by these cyclic compounds might be attributed to their binding affinity to these enzymes^53^. Moreover, the structure-activity relationship in addition to the number and orientation of the amino groups in the synthetic compounds might be related to their inhibitory mechanism on these enzymes^54^.

**Table 5:** Anti-diabetic efficacy in vitro at equivalent concentrations (1000 µg/mL) of the various synthesized substances.

Sample	Anti-diabetic activity	
	α-amylase	α-glucosidase
	Inhibition (%)	
1	16.94 ± 0.03	6.79 ± 0.03
2	**59.15 ± 0.29**	**49.00 ± 0.29**
3	40.03 ± 0.26	29.88 ± 0.26
4	17.19 ± 0.03	7.04 ± 0.03
5	13.67 ± 0.03	3.52 ± 0.03
6	34.88 ± 0.22	24.73 ± 0.22
7	17.33 ± 0.03	7.18 ± 0.03
8	29.48 ± 0.22	19.33 ± 0.22
9	35.74 ± 0.23	25.59 ± 0.23
10	**59.65 ± 0.29**	**49.50 ± 0.29**
11	52.61 ± 0.25	42.46 ± 0.25
12	53.32 ± 0.26	43.17 ± 0.26

	Acarbose	
STD	70.05 ± 0.15	59.90 ± 0.15

The most active sample is indicated by the bold, which represents the mean ± standard error (SE) of the data, which were computed from three replicates.

**Table 6:** The median inhibitory concentrations (IC_50_) of the different synthetic compounds against DPPH and ABTS radicals.

Sample	Scavenging activity		Anti-diabetic activity	
	DPPH	ABTS	α-amylase	α-glucosidase
	IC_50_ (mg/mL)			
1	22.67 ± 0.14	16.26 ± 0.14	13.15 ± 0.03	18.79 ± 0.06
2	**5.17 ± 0.04**	**4.66 ± 0.04**	**3.77 ± 0.01**	**2.60 ± 0.01**
3	7.95 ± 0.07	6.88 ± 0.06	5.57 ± 0.03	4.27 ± 0.03
4	22.22 ± 0.14	16.02 ± 0.14	12.96 ± 0.03	18.11 ± 0.05
5	30.74 ± 0.20	20.15 ± 0.17	16.30 ± 0.04	36.27 ± 0.12
6	9.29 ± 0.09	7.90 ± 0.07	6.39 ± 0.03	5.16 ± 0.04
7	21.98 ± 0.14	15.89 ± 0.14	12.85 ± 0.03	17.76 ± 0.05
8	11.30 ± 0.12	9.35 ± 0.08	7.56 ± 0.04	6.60 ± 0.06
9	9.04 ± 0.09	7.71 ± 0.06	6.23 ± 0.03	4.99 ± 0.04
10	**5.12 ± 0.04**	**4.62 ± 0.04**	**3.73 ± 0.01**	**2.58 ± 0.01**
11	5.87 ± 0.04	5.24 ± 0.04	4.23 ± 0.02	3.01 ± 0.02
12	5.79 ± 0.04	5.17 ± 0.04	4.18 ± 0.02	2.96 ± 0.02

	Ascorbic acid		Acarbose	
STD	4.75 ± 0.02	4.31 ± 0.04	3.18 ± 0.02	2.13 ± 0.02

Significance value bold.

## Experimental section

### Material and method

The target compounds were synthesized using premium ingredients. Solvents (ethanol 99.8%, pyridine, acetic anhydride, and acetic acid 99.7%) were provided by Sigma-Aldrich Company. (Mg (NO_3_)_2_.4H_2_O - BHD Laborator, Suppliers, 99%).

### Preparation of Mn_3_O_4_ nanoparticles

To produce a (0.4 M) solution, 10 g of manganese nitrate was dissolved in 500 mL of distilled water. At room temperature, the manganese nitrate solution was agitated and dripped with a 2 M NaOH solution until the mixture’s pH = 10. The solid precipitation starts white at this pH and soon turns brown. Two hours were spent on the chemical reaction while the precipitate was allowed to boil overnight. The brownish precipitate was then filtered and dried for four hours at 100^o^C after being repeatedly cleaned with distilled water to remove the excess NaOH.

### Characterization

XRD is carried out using a [Bruker D8 advance diffractometer, Germany] and Cu Kα radiation (λ = 1.5406 Å). The surface structure of the as-prepared Mn_3_O_4_-NPs was investigated using microscopic analysis, for instance, field emission scanning electron microscopy (FE-SEM, model FEJ Quanta 250 Fei, Netherlands), in addition to energy dispersive X-ray analysis (EDX). Furthermore, the crystallinity and nature of the nanoparticles were observed using a high-resolution transmission electron microscope (HR-TEM; Joel model JEM-2100; Japan; operating voltage 200 kV). Melting points were restrained without correction using a digital Electrothermal IA 9100 Series instrument from Cole-Parmer (Beacon Road, Stone, Staffordshire, ST15 OSA, UK). A Perkin Elmer CHN 2400 was exploited for C, H, and N analyses. Using KBr wafers, FT-IR 460 PLUS was used to record IR spectra between 4000 and 400 cm^− 1^. At the National Research Centre (NRC), the ^1^H and ^13^C-NMR spectra were confirmed using a JEOL 500 MHz NMR spectrometer and DMSO-d_6_ as a solvent. Chemical shifts were expressed as δ (ppm).

### Preparation of organic derivative products

#### Product 2: 6-(3-Nitrophenyl)-4-(*p*-tolyl)-2*H*-1,3-thiazin-2-amine

#####  Nano catalytic method

After two hours of reflux heating in 20 milliliters of ethanol, a mixture of compound **1** (0.01 mmol) and thiourea (0.01 mmol) in the presence of nano Mn_3_O_4_ (0.025 mmol) was cooled to room temperature. The resulting precipitate was filtered, dried, and then crystallized in ethanol to provide a final product **2** (brown orange crystal, m.p. above 300 °C) with a yield of 65%.

##### Traditional method

After the reaction was completed, the mixture was cooled to room temperature. Compound **1** (0.01 mol) and thiourea (0.01 mol) were heated under reflux in 20 mL of ethanol for five hours. KOH (0.025 mol) was also present in this combination. The resulting precipitate was collected by filtration, dried, and then crystallized with ethanol to provide product **2**, a brown-orange crystal with a yield of 47% and a melting point (m.p.) of greater than 300 °C. IR (KBr, ν cm^− 1^): 3335.69 (NH_2_), 1658.25 (C = N str), 1167.11 (C-S str); ^1^H-NMR (DMSO-d_6_, 500 MHz): δ (ppm) 2.50 (s, 3 H, CH_3_), 3.47–3.49 (m, 2 H, CH_2_-thiazine),4.55–4.64 (m, 1H, CH-thiazine), 5.31 (s, 1H, CH), 8.76 (s, 2 H, NH_2_) 7.28–8.62 (m, 8 H, Ar-H); MS (*m/z*) (%): 327.10 (M+, 9), 90.79 (100); Elemental analysis: Calcd for C_17_H_17_N_3_O_2_S (327.10) C, 62.37; H, 5.23; N, 12.83; S, 9.79%; Found: C, 62.36; H, 5.24; N, 12.82; S, 9.79%.

#### Product 3: 1-(5-(3-Nitrophenyl)-3-(*p*-tolyl)-4,5-dihydro-1*H*-pyrazol-1-yl)ethan-1-one

Compound **1** (0.01 mol) and hydrazine hydrate (0.01 mol) were combined with 30 mL of acetic acid and heated under reflux for six hours. The reaction mixture was then cooled, and the separated solid product was filtered, dried, and crystallized from ethanol to get compound **3**. 68% yield, buff crystals; m.p. (160–165) ℃. IR (KBr, v cm^− 1^): 1650.39 (C = O str), 1592.76 (C = N str), and 1267.37 (C-N str); ^1^H-NMR (DMSO-d_6_, 500 MHz): δ (ppm) 2.50 (s, 3 H, CH_3_), 3.33 (s, 3 H, CH_3_ for acetyl group), 3.83-3-93 *j* coupling = 10 (m, 2 H, CH_2_-pyrazole), 5.67–5.72 *j* coupling = 5 (m, 1H, CH-pyrazole), 7.26–8.12 (m, 8 H, Ar–H); ^13^C-NMR (DMSO-d_6_, 100 MHz): δ (ppm) 21.01, 21.46, 38.65, 38.94, 39.22, 39.49, 39.77, 40.05, 40.33, 41.81, 58.77, 120.58, 122.22, 126.68, 128.10, 129.33, 130.32, 132.34, 140.31, 144.44, 147.88, 154.29, 167.62 (C= O); Elemental analysis: Calcd for C_18_H_17_N_3_O_3_ (323.13) C, 66.86; H, 5.30; N, 13.00%; Found: C, 66.87; H, 5.31; N, 12.98%.

#### Product 4: 5-(3-Nitrophenyl)-3-(*p*-tolyl)-4,5-dihydro-1*H*-pyrazole-1-carbaldehyde

Compound **4** was produced by heating a mixture of compound 1 (0.01 mol) and hydrazine hydrate (0.01 mol) in 30 mL of formic acid under reflux for four hours, cooling the reaction mixture after the separated solid product was filtered, dried, and crystallized from ethanol. Yield 70%, yellow crystals; m.p. (165–170) ^o^C. IR (KBr, ν cm^− 1^): 1709.04 (C = O str), 1594.44 (C = N str), 1268.75 (C-N str); ^1^H-NMR (DMSO-d_6_, 500 MHz): δ (ppm) 2.50 (s, 3 H, CH_3_), 3.89–3.99 *j* coupling = 5 (m, 2 H, CH_2_-pyrazole), 5.68–5.73 *j* coupling = 5 (m, 1H, CH-pyrazole), 7.26–8.12 (m, 8 H, Ar–H), 8.92 (s, 1H, CH-aldehyde); ^13^C-NMR (DMSO-d_6_, 100 MHz): δ (ppm) 21.02, 38.94, 39.22, 39.50, 39.77, 40.05, 41.99, 57.78, 120.80, 122.51, 126.77, 127.77, 129.39, 130.43, 132.57, 140.61, 143.28, 144.10, 147.92, 156.20, 158.99, 159. 80 (C=O); Elemental analysis: Calcd for C_17_H_15_N_3_O_3_ (309.11) C, 66.01; H, 4.89; N, 13.58%; Found: C, 66.00; H, 4.90; N, 13.59%.

#### Product 5: 2-Hydroxy-1-(5-(3-nitrophenyl)-3-(*p*-tolyl)-4,5-dihydro-1*H*-pyrazol-1-yl)ethan-1-one.

Compound **5** was produced by heating a mixture of compound 1 (0.01 mol) and hydrazine hydrate (0.01 mol) in 30 mL of glycolic acid under reflux for six hours, cooling the reaction mixture after the separated solid product was filtered, dried, and crystallized from ethanol. Yield: 70%; melting point: 145–150 ℃; pale yellow crystals. IR (KBr, ν cm^− 1^): 3224.26-3463.16 (OH), 1743.87 (C = O str), 1643.85 (C = N str), 1279.39 (C-N str); ^1^H-NMR (DMSO-d_6_, 500 MHz): δ (ppm) 2.50 (s, 3 H, CH_3_), 3.83–4.84 *j* coupling = 10 (m, 2 H, CH_2_-pyrazole), 5.44 (s, 2 H, CH_2_-OH), 5.69–5.75 *j* coupling = 10 (m, 1H, CH-pyrazole), 7.27–8.15 (m, 8 H, Ar-H), 5.44 (s, 1H, OH); ^13^C-NMR (DMSO-d_6_, 100 MHz): δ (ppm) 21.09, 38.66, 38.94, 39.22, 39.49, 39.77, 40.05, 41.32, 59.12, 59.40, 60.45, 60.81, 120.82, 122.40, 126.86, 128.00, 128.74, 129.41, 130.45, 132.56, 140.55, 144.22, 144.10, 147.95, 155.06, 169.70 (C = O), 170.02 ; MS (*m/z*) (%): 339.12 (M+, 9), 159.04 (100); Elemental analysis: Calcd for C_18_H_17_N_3_O_4_ (339.12) C, 63.71; H, 5.05; N, 12.38%; Found: C, 63.65; H, 5.03; N, 12.39%.

#### Product 6: (3-(3-nitrophenyl)-5-(*p*-tolyl)-2,3-dihydro-1*H*-pyrazol-1-yl)(phenyl)methanone.

Compound **1** (0.01 mol) and benzoylhydrazide (0.01 mol) were combined and heated under reflux in 20 mL of ethanol for six hours. Following the reaction, the mixture was allowed to cool to room temperature. The mixture also contained 40% KOH (5 mL). The resulting precipitate was filtered, dried, and then crystallized by ethanol to provide a final product **6** with a yield of 42%, a pale yellow crystal with a melting point of between 245 and 250 °C. IR (KBr, ν cm^− 1^): 3156.80 (NH), 1667.79 (C=O str), 1284.74 (C-N str); ^1^H-NMR (DMSO-d_6_, 500 MHz): δ (ppm) 2.35–2.36 *j* coupling = 5 (d, 1H, CH-pyrazole), 2.50 (s, 3 H, CH3), 3.56–3.57 (m, 1H, CH-pyrazole), 7.29–8.14 (m, 13H, Ar–H), 10.49 (s, 1H, NH); ^13^C-NMR (DMSO-d_6_, 100 MHz): δ (ppm) 38.65, 38.93, 39.21, 39.48, 39.76, 40.04, 40.32, 127.47, 128.05, 128.54, 128.91, 129.23, 129.43, 131.89, 132.56, 165.85 (C= O); MS (*m/z*) (%): 385.14 (M+, 9), 68.86 (100); Elemental analysis: Calcd for C_23_H_19_N_3_O_3_ (385.14) C, 71.68; H, 4.97; N, 10.90%; Found: C, 71.69; H, 4.98; N, 10.91%.

#### Product 7: 5-(3-Nitrophenyl)-3-(*p*-tolyl)-2,5-dihydroisoxazole

Compound **7** was produced by heating a mixture of compound 1 (0.01 mol) and hydroxylamine hydrochloride (0.01 mol) in 15 mL dioxane under reflux for six hours, cooling the reaction mixture after the separated solid product was filtered, dried, and crystallized from ethanol. 55% yield; m.p. (140–150 ℃); yellow crystals. IR (KBr, ν cm^− 1^): 3191.42 (NH), 1662.06 (C =C str), 1254.38 (C–N str); ^1^H-NMR (DMSO-d_6_, 500 MHz): δ (ppm) 2.50 (s, 3 H, CH_3_), 3.56–3.57 (d, 1H, CH-pyrazole), 6.88–6.93 (d, 1H, CH–O), 7.17–8.16 (m, 8 H, Ar–H), 11.70 (s, 1H, NH); MS (*m/z*) (%): 280.10 (M+, 9), 91.02 (100); Elemental analysis: Calcd for C_16_H_14_N_2_O_3_ (282.10) C, 68.08; H, 5.00; N, 9.92%; Found: C, 68.09; H, 4.99; N, 9.93%.

#### Product 8: 3-Imino-5-(3-nitrophenyl)-7-(*p*-tolyl)-2, 3-dihydro-1*H*, 5*H*-pyrazolo-[1,2-*a*]pyrazol-1-one

#####  Nano catalytic method

Compound **1** (0.01 mmol), cyanoacetohydrazide (0.01 mmol), and nano Mn_3_O_4_ (0.025 mmol) were combined and heated under reflux in 20 mL of ethanol for two hours. The reaction was then finished, and the mixture was allowed to cool to room temperature. The resulting precipitate was filtered, dried, and then crystallized by ethanol to provide a final product **8** with a 45% yield - a yellow crystal.

##### Traditional method

Compound **1** (0.01 mol) and cyanoacetohydrazide (0.01 mol) were combined and heated for six hours under reflux in 20 mL of ethanol. During recuperation, the reactant did not change. IR (KBr, v cm^− 1^): 3069.41 (NH), 1677.59 (C= O str), 1604.14 (C=N str); ^1^H-NMR (DMSO-d_6_, 500 MHz): δ (ppm) 2.50 (s, H, CH_3_), 3.20–3.23 *j* coupling = 10 (d, 1H, CH-pyrazole), 4.38–4.40 *j* coupling = 10 (d, 1H, CH-pyrazole), 7.28–8.62 (m, 8 H, Ar–H), 8.68 (s, 1H, NH); Elemental analysis: C, 65.51; H, 4.63; N, 16.08%; Found: C, 65.49; H, 4.65; N, 16.07%; Calcd for C_19_H_16_N_4_O_3_ (348.36).

#### Product 9: 5-(3-Nitrophenyl)-3-(*p*-tolyl)-4,5-dihydro-1*H*-pyrazole-1-carbothio-amide

#####  Nano catalytic method

Compound **1** (0.01 mmol) and thiosemicarbazide (0.01 mmol) were combined with nano Mn_3_O_4_ (0.025 mmol) and heated under reflux in 20 mL of acetic acid for two hours. Following the reaction, the mixture was allowed to cool to room temperature. After filtration, drying, and ethanol crystallization, the resulting precipitate was collected, yielding a final product with a yield of 70%, a yellow crystal with a melting point of 165–170 ℃.

##### Traditional method

For six hours, a solution containing compound **1** (0.01 mol) and thiosemicarbazide (0.01 mol) was heated under reflux in 20 mL of acetic acid. Cooling the reaction mixture allowed the separated solid product to be filtered off, dried, and crystallized from the ethanol, yielding yellow crystals of product **9** with a melting point of (165–170) ℃ and yield: 62%. IR (KBr, ν cm^− 1^): 3259.26 (NH), 1642.46 (C= N str), 1162.41 (C= S str); ^1^H-NMR (DMSO-d_6_, 500 MHz): δ (ppm) 2.50 (s, H, CH_3_), 3.31–3.49 *j* coupling = 25 (m, 2 H, CH_2_-pyrazole), 5.57–5.59 *j* coupling = 10 (m, 1H, CH-pyrazole), 7.28–8.62 (m, 8 H, Ar–H), 8.73 (s, 2 H, NH_2_); ^13^C-NMR (DMSO-d_6_, 100 MHz): δ (ppm) 20.77, 21.30, 38.67, 38.94, 39.22, 39.50, 39.78, 40.05, 40.32, 42.71, 59.28, 82.48, 86.95, 89.58, 121.29, 122.51, 123.10, 124.07, 124. 85, 128.15, 128.98, 129.41, 129.62, 132.50, 133.42, 133.96, 135.24, 135.61, 140.21, 140.65, 143.60, 143.83, 144.09, 147.69, 148.21, 181.04 (C = S); MS (*m/z*) (%): 340.10 (M+, 9), 90.75 (100); Elemental analysis: Calcd for C_17_H_16_N_4_O_2_S (340.10) C, 59.98; H, 4.74; N, 16.46; S, 9.42%; Found: C, 59.96; H, 4.72; N, 16.48; S, 9.41%.

#### Product 10: 2-(3-Nitrophenyl)-4-(*p*-tolyl)-2,3-dihydrobenzo[*b*]^1,4^thiazepine

#####  Nano catalytic method

Compounds **1** (0.01 mmol) and 2-aminothiophenol (0.01 mmol) were combined with nano Mn_3_O_4_ (0.025 mmol) and heated under reflux in 20 mL of acetic acid for two hours. The reaction was then finished, and the mixture was allowed to cool to room temperature. After filtration, drying, and ethanol crystallization, the resulting precipitate was collected, yielding a final product with a yield of 60%, a yellow-green crystal with a melting point of 190–200 ℃.

##### Traditional method

Twenty milliliters of acetic acid were heated under reflux for six hours to combine compound **1** (0.01 mol) and 2-aminothiophenol (0.01 mol). Product **10** was produced by filtering out, drying, and crystallizing the separated solid product from the ethanol after the reaction mixture was cooled. The product was yellow-green crystals of m.p. (190–200) ℃; yield: 44%; IR (KBr, v cm^− 1^): 1673.94 (C= str), 1161.69 (C–S str); ^1^H-NMR (DMSO-d_6_, 500 MHz): δ (ppm) 2.07 (t, 1H, CH-thiazepine), 2.50 (s, 3 H, CH_3_), 3.43–3.45 *j* coupling = 10 (m, 2 H, CH_2_-thiazepine), 7.49–8.45 (m, 12 H, Ar–H); ^13^C-NMR (DMSO-d_6_, 100 MHz): δ (ppm) 20.77, 21.30, 38.67, 38.94, 39.22, 39.50, 39.78, 40.05, 40.32, 42.71, 59.28, 82.48, 86.95, 89.58, 121.29, 122.51, 123.10, 124.07, 124.85, 128.15, 128.98, 129.41, 129.62, 132.50, 133.42, 133.96, 135.24, 135.61, 136.72, 140.21, 140.65, 143.60, 143.83, 144.09, 147.69, 148.21, 181.04 (C= S); MS (*m/z*) (%): 374.11 (M+, 9), 118.79 (100); Elemental analysis: Calcd for C_22_H_18_N_2_O_2_S (374.11) C, 70.57; H, 4.85; N, 7.48; S, 8.56%; Found: C, 70.55; H, 4.86; N, 7.49; S, 8.55%.

#### Product 11: 2-(3-Nitrophenyl)-4-(*p*-tolyl)-2,3-dihydro-1*H*-benzo[*b*]^1,4^diazepine.

#####  Nano catalytic method

Compound **1** (0.01 mmol), *o*-phenylenediamine (0.01 mmol), and nano Mn_3_O_4_ (0.025 mmol) were combined and heated under reflux in 20 mL of acetic acid for two hours. The reaction was then finished, and the mixture was allowed to cool to room temperature. The resulting precipitate was filtered, dried, and then crystallized in ethanol to provide a final product (**11**), a red-brown crystal with a 66% yield and a melting point of 200–210 ^o^C.

##### Traditional method

For six hours, a mixture of compound **1** (0.01 mol) and *o*-phenylenediamine (0.01 mol) was heated under reflux in 20 mL of ethanol with 5 mL of 40% KOH. Red-brown crystals of product **11** were obtained by filtering out, drying, and crystallizing the separated solid product from the ethanol after the reaction mixture had cooled, the yield was 50% at the melting point (200–210 ℃). IR (KBr, ν cm^− 1^): 3357.31(NH), 1658.07 (C=N str), 1263.69 (C–N str); ^1^H-NMR (DMSO-d_6_, 500 MHz):δ (ppm) 2.50 (s, 3 H, CH_3_), 3.32–2.46 (m, 2 H, CH_2_-thiazepine), 6.37–6.50 (m, H, CH-thiazepine), 7.03–7.86 (m, 12 H, Ar–H), 8.83 (s, H, NH); Elemental analysis: Calcd for C_22_H_19_N_3_O_2_ (375.15) C, 73.93; H, 5.36; N, 11.76%; Found: C, 73.92; H, 5.37; N, 11.75%.

#### Product 12: 7-Nitro-2-(3-nitrophenyl)-4-(*p*-tolyl)-2,3-dihydro-1*H*-benzo[*b*]^1,4^diazepine

##### Nano catalytic method

Compound **1** (0.01 mmol), *p*-nitro-*o*-phenylinediamine (0.01 mmol), and nano Mn_3_O_4_ (0.025 mmol) were combined and heated under reflux in 20 mL of acetic acid for two hours. Following the reaction, the mixture was allowed to cool to room temperature. Filtration was used to collect the precipitate, which was subsequently dried and crystallized by ethanol to produce a final product (**12**) with a 70% yield and a brown crystal melting point of 210–205 ^o^C.

##### Traditional method

For six hours, a mixture of compound **1** (0.01 mol) and *p*-nitro-*o*-phenylinediamine (0.01 mol) was heated under reflux in 20 mL of ethanol with 5 mL of 40% KOH. After cooling the reaction mixture, the separated solid product was filtered out, dried, and crystallized from the ethanol to produce brown crystals of product **12** with 55% yield at m.p.(210–215)℃. IR (KBr, ν cm^− 1^): 3352.16 (NH), 1659.81 (C= N str), 1282.53 (C–N str); ^1^H-NMR (DMSO-d_6_, 500 MHz): δ (ppm) 2.50 (s, 3 H, CH_3_), 3.32–3.46 (m, 2 H, CH_2_-thiazepine), (m, H, CH-thiazepine), 7.19–7.86 (m, 11 H, Ar–H), 8.33 (s, H, NH); Elemental analysis: Calcd for C_22_H_18_N_4_O_4_ (402.41) C, 65.66; H, 4.51; N, 13.92%; Found: C, 65.67; H, 4.52; N, 13.91%.

### In vitro biological activities material and method

Every experiment was performed in triplicate, and all biological activities were evaluated in the produced samples at equal doses of 1000 µg/mL.

#### Antioxidant activity

The total antioxidant capacity (TAC) was determined according to the method proposed by^55^. The result was expressed as mg gallic acid equivalent per gram weight, using the same amounts of ascorbic acid as a standard. The iron-reducing power (IRP) in µg/mL was determined using the procedure recommended by^56^. A stronger reducing power is indicated by a high absorbance at 700 nm, using ascorbic acid as a reference.

#### Scavenging activity

##### DPPH radical-scavenging activity

Using the procedure outlined by Rahman et al., the 1,1-Diphenyl-2-picryl-hydrazyl (DPPH) radical scavenging activity was assessed^57^. A positive control with the same concentration was ascorbic acid. It was determined what percentage of the DPPH free radical was inhibited. Plotting a series of sample concentrations against the percent of DPPH inhibition allowed for the determination of the median inhibitory concentration (IC_50_) for each examined sample.

##### ABTS radical scavenging assay

The protocol recommended by Arnao et al. was used for the 2,2′-azinobis-(3-ethylbenzothiazoline-6-sulfonic acid) (ABTS) test^58^. The ability of the samples and ascorbic acid to scavenge ABTS was compared. This fraction of the ABTS radical was found to be inhibited. For each component under test, the IC_50_ was determined by plotting a curve with a range of sample concentrations against the percentage of ABTS inhibition.

#### Anti-diabetic activity

The assay determined the percentage of α-amylase^59^ and α-glucosidase^60^ enzyme inhibition using acarbose as the standard drug. The IC_50_ was calculated for each tested sample by plotting a series of sample concentrations against the percentage of enzyme inhibition.

## Conclusions

The yield and reaction time of certain reactions were enhanced by green feeding with Mn_3_O_4_, whereas benzalacetophenone derivatives generated non-isolable thia Michel adducts that subsequently underwent cyclo-condensation and dehydrogenation to create thiazine **2**. Pyrazole heterocyclization was created by Target **1** and hydrazine at theoretical quantities in an acidic medium. This was followed by N-acylation, which produced **3**,** 4**, and **5**. To produce a pyrazoline derivative, compound **1** and benzoyl hydrazine cyclocondensed. 6. Oxazole compounds are created by hydroxyleamine hydrochloride undergoing oxa Michel, cyclodehydration, and cyclocondensation **7**. Cyanoacetohydrazide with product **1** cyclized to pyrazol **8**, and thioamide derivatives **9** resulted from cyclocondensation of **1** with thiosemicarbazide. Chalcone **1** was cyclized with 2-aminothiophenol, resulting in the formation of thiazapine **10**, diazapine **11**, and **12** generated from the reaction of *o*-phenylenediamine and 3-nitro-*o*-phenylenediamine in a basic medium with chalcone **1**. Due to their significant biological and pharmacological properties, azoles and their derivatives are key compounds in organic chemistry. Compared to standard ascorbic acid, all synthesized compounds showed strong antioxidant activity against DPPH and H_2_O_2_ radicals, with even higher activity against SOR and NO radicals. Compounds **2**, **3**, and **10** exhibited the most potent anti-diabetic effects, surpassing regular acarbose.

These biological findings were further validated by molecular docking experiments using the MOE software, which highlighted the activity of compounds **2**,** 3**, and **10** using acarbose as the reference ligand and GacH as a protein that binds maltose/maltodextrin.

## Electronic supplementary material

Below is the link to the electronic supplementary material.

Supplementary Material 1.

## Data Availability

Data is provided within the manuscript or supplementary information files.
