# Supplementary material for: Synthesis, molecular docking, assessment of biological and anti-diabetic properties of benzalacetophenone derivatives
Source: Sci Rep. 2025 Apr 23;15:14159. doi: 10.1038/s41598-025-96610-6 (PMC12019235; doi:10.1038/s41598-025-96610-6)
Supplement: Supplementary file 1 — Supplementary Material 1. [file 41598_2025_96610_MOESM1_ESM.pdf]

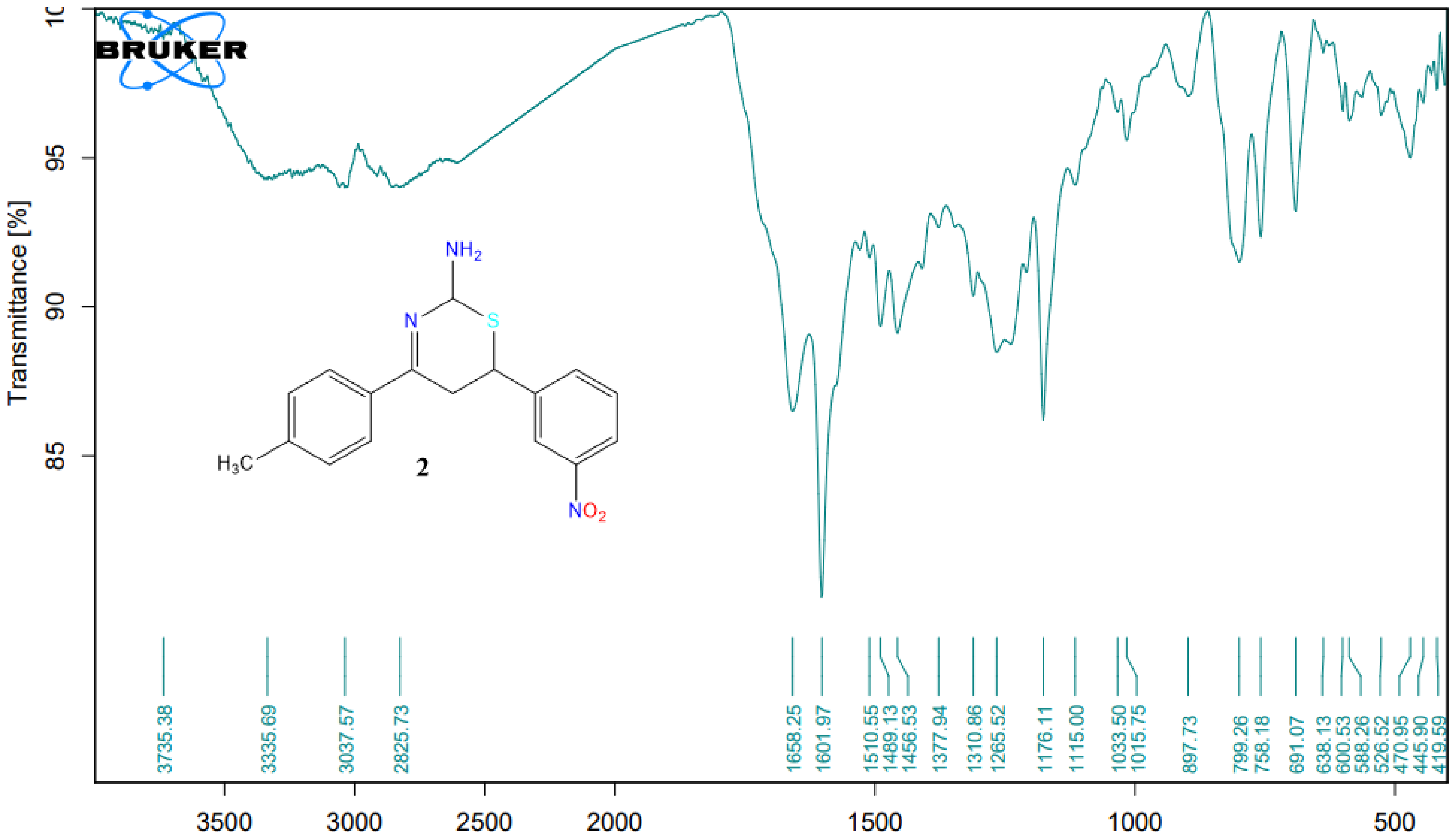

S1: FT-IR spectrum of compound 2 showing characteristic absorption bands at **3335.69 cm⁻¹** (NH_2 stretching), **1658.25 cm⁻¹** (C=N stretching), and **1176.11 cm⁻¹** (C-S stretching).

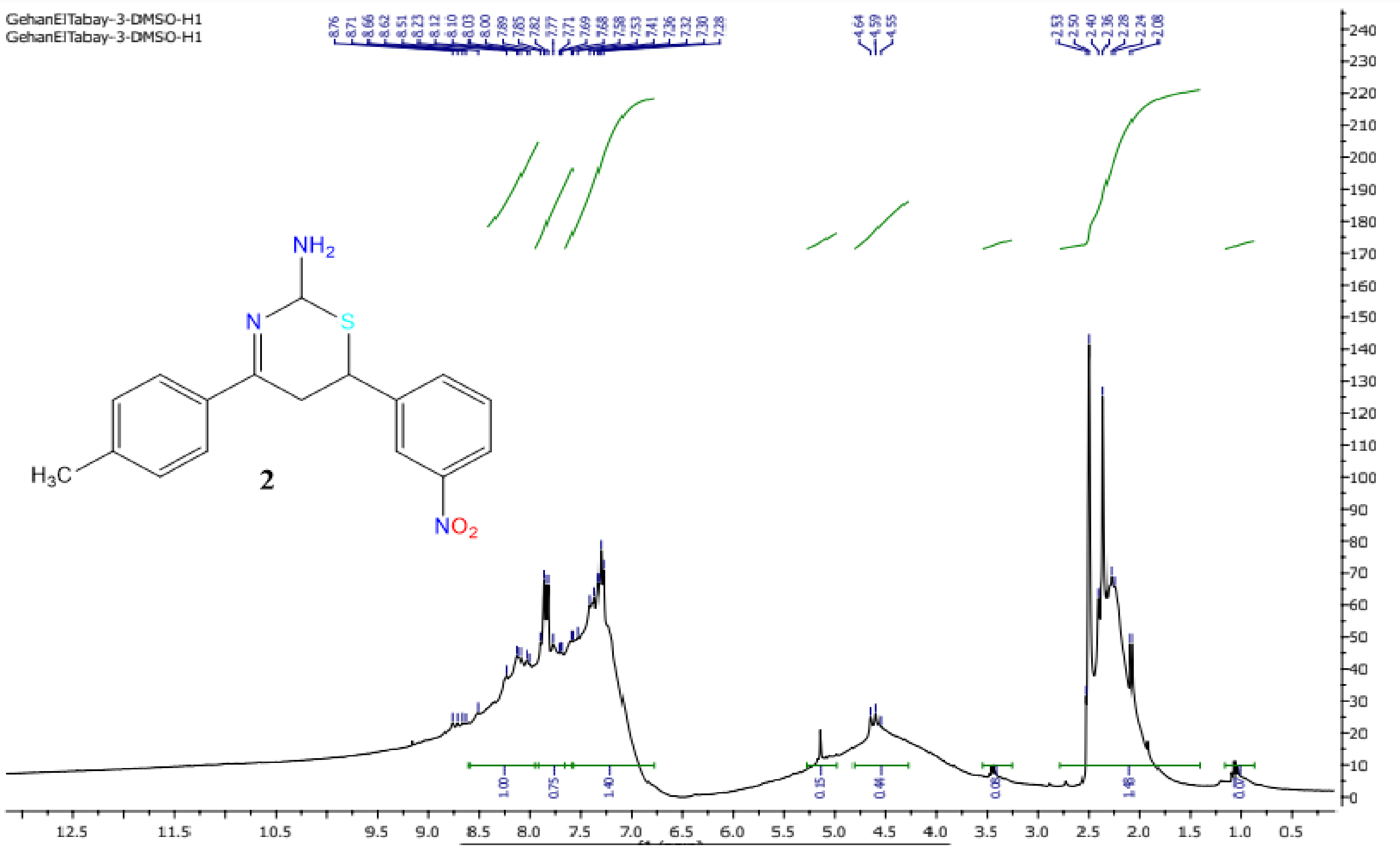

S2: ¹H NMR spectrum of compound 2 recorded in **DMSO-d_6**. The spectrum shows characteristic signals at δ 2.50 ppm (CH_3), δ 3.47–3.49 ppm (CH), δ 4.55–4.64 ppm (CH_2, multiplet), δ 5.31 ppm (CH), and δ 8.76 ppm (NH_2).

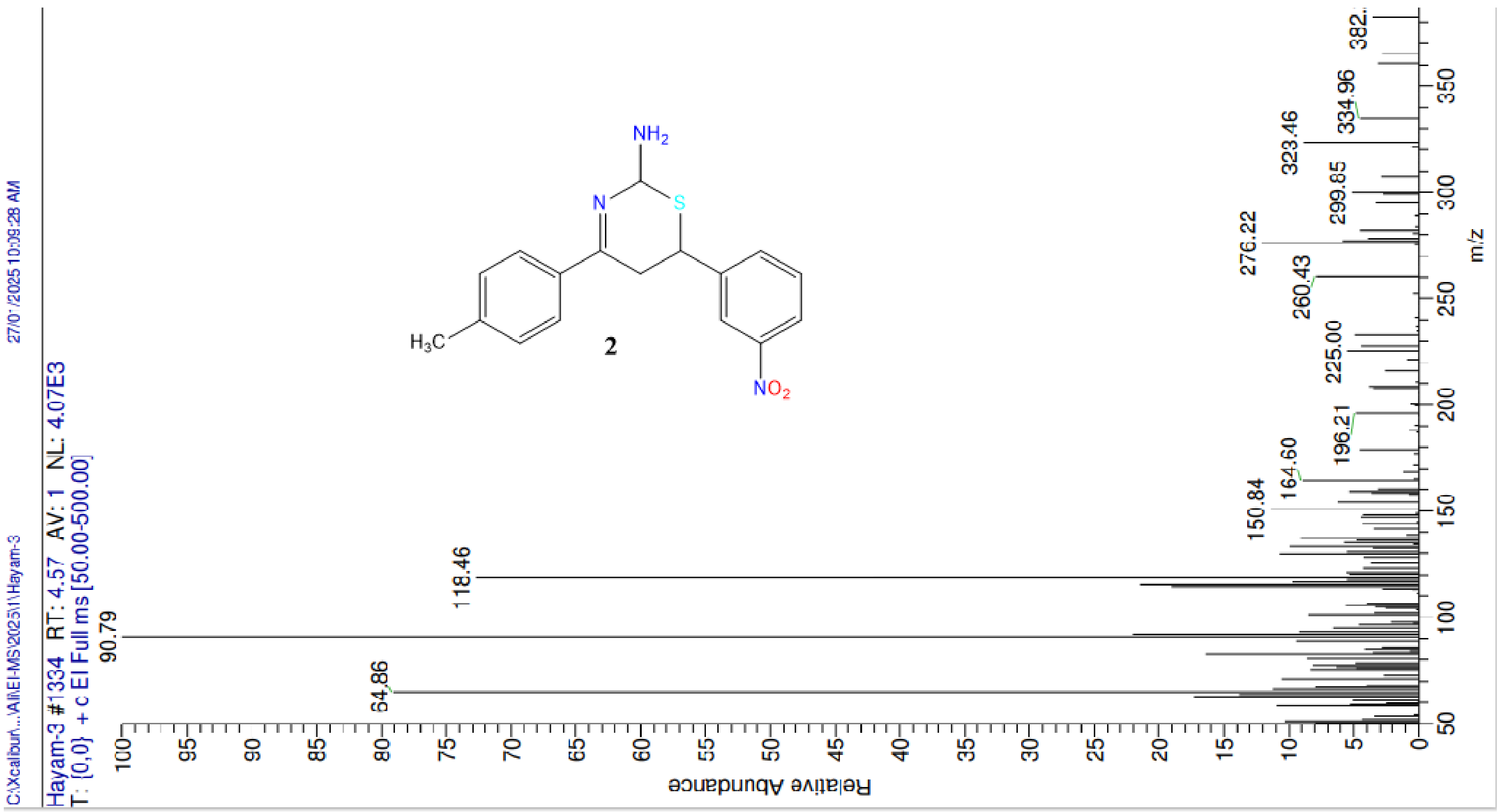

S3: Mass Spectrum of Compound 2. The mass spectrum of **compound 2** shows a molecular ion peak at **m/z = 327.10**, confirming the molecular weight of the synthesized compound. Fragmentation peaks at **m/z = 323.46, 299.85, 260.43, 225.00, and 118.46** correspond to characteristic fragment ions, supporting the proposed structure.

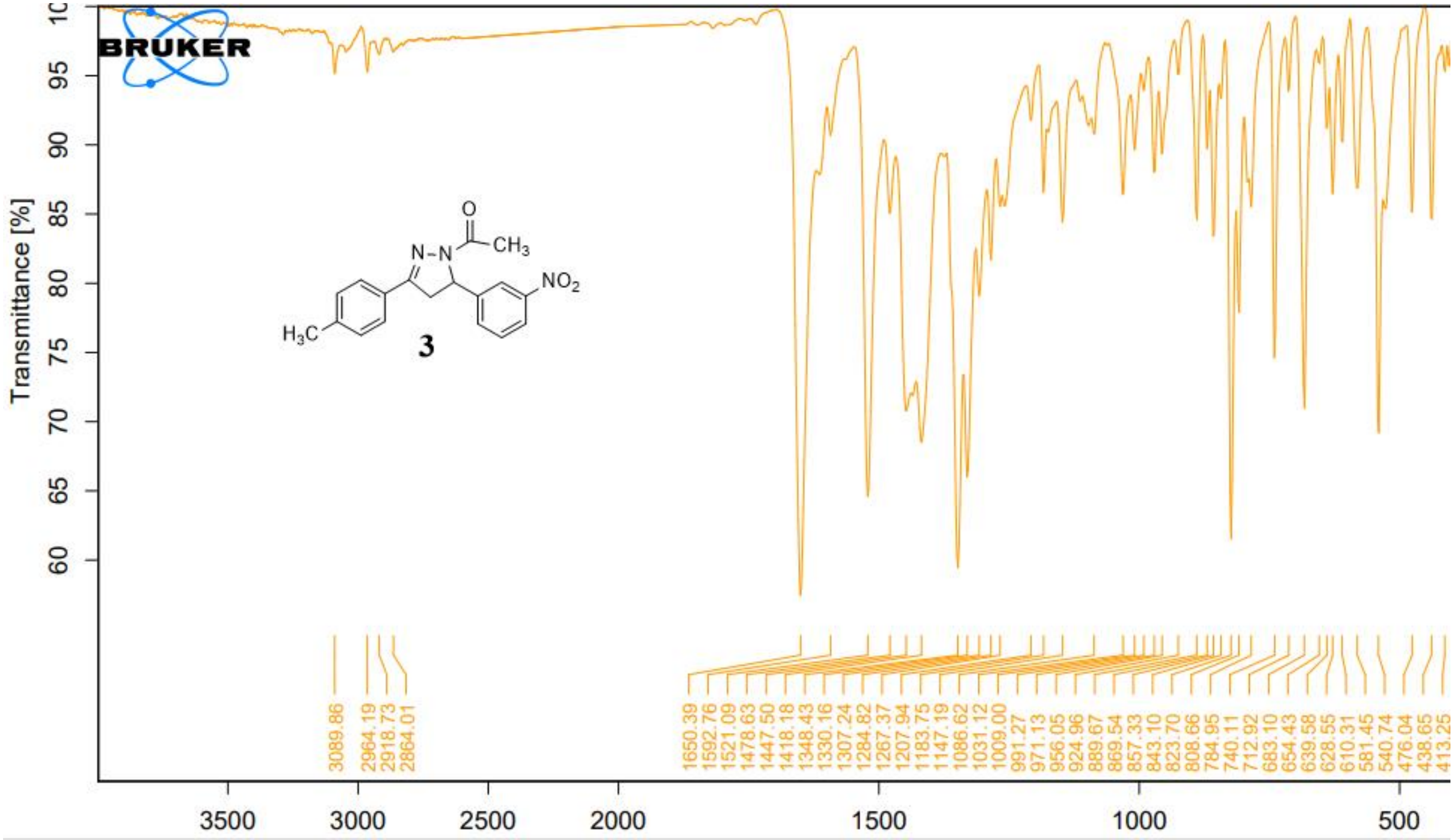

S4: Infrared (IR) Spectrum of Compound 3. The IR spectrum of **compound 3** revealed an absorption band at **1650.39 cm⁻¹**, corresponding to the **C=O** stretching vibration. Additional bands at **3089.86 cm⁻¹** and **2963.19 cm⁻¹** were attributed to aromatic and aliphatic C-H stretching, respectively.

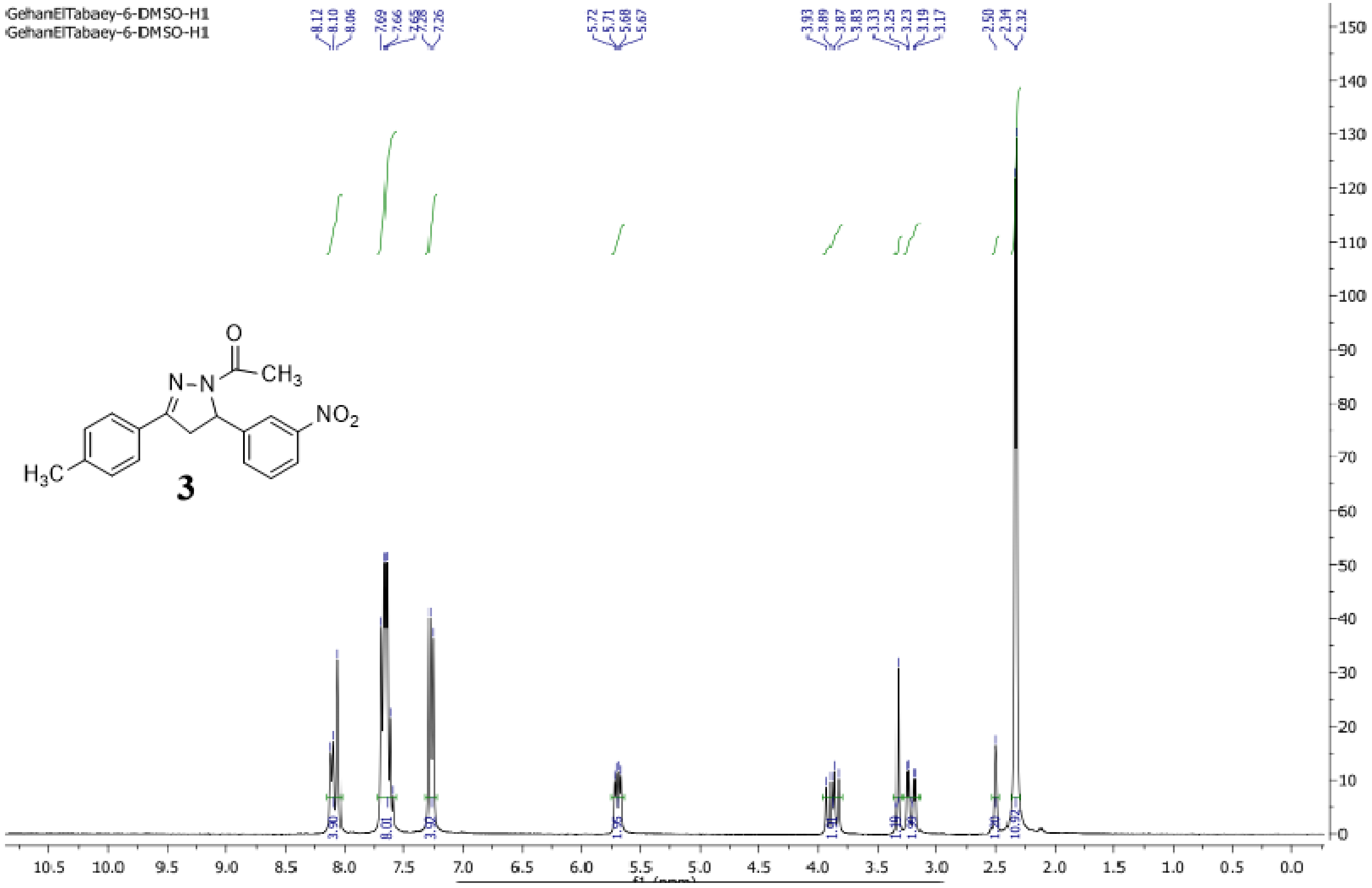

S5: ^{1} H-NMR Spectrum of Compound 3. ^{1}H-NMR spectrum of **compound 3** displayed a **singlet** at **δ 2.50 ppm**, corresponding to the **methyl (-CH_3) group**. Additionally, **multiplet signals** were observed at **δ 3.83–3.93 ppm**, which can be attributed to the **methylene (-CH_2) protons**, while another set of **multiplet signals** appeared at **δ 5.67–5.72 ppm**, assigned to the **methine (-CH) proton**.

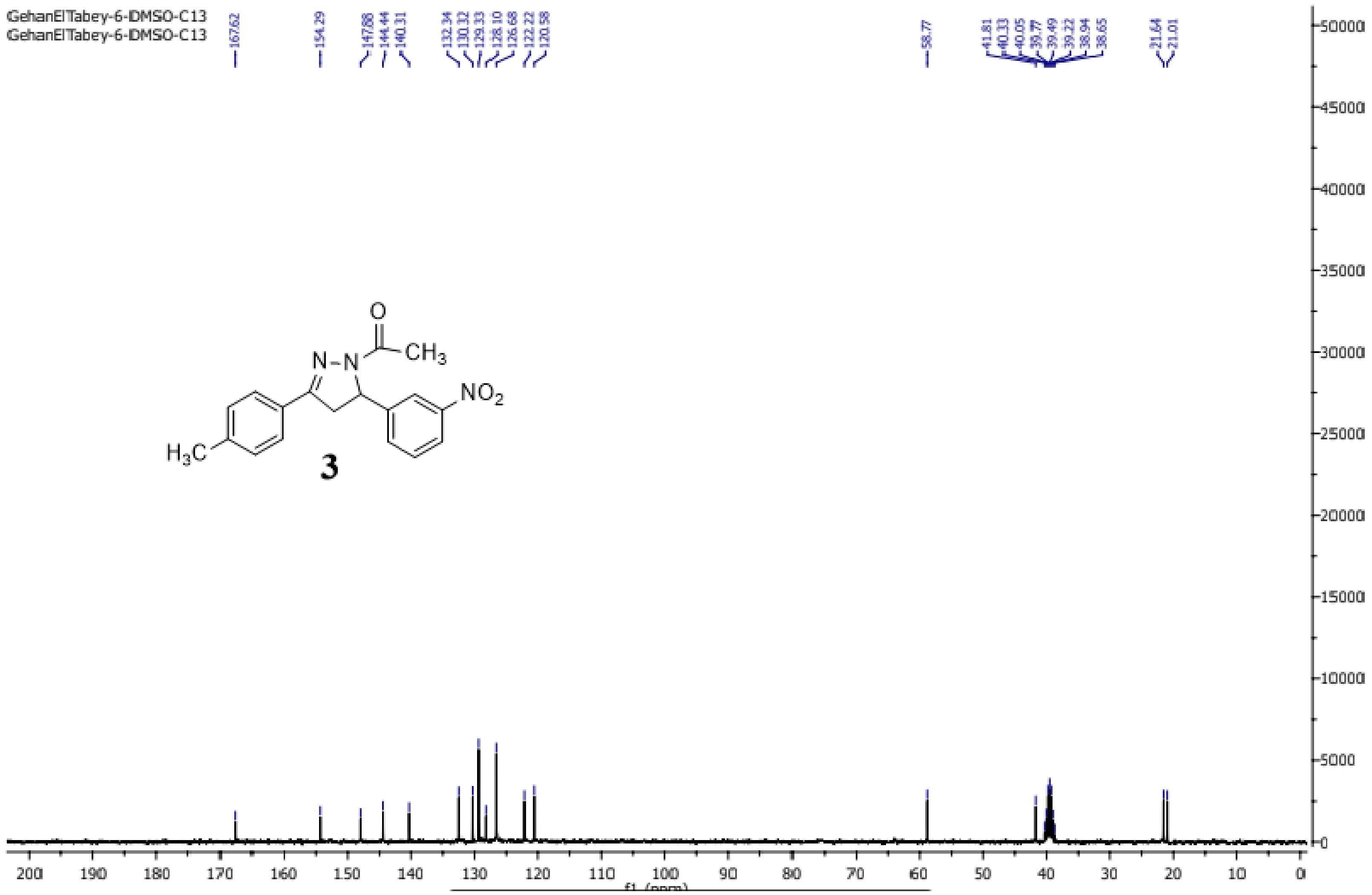

S6: ^{13}C-NMR Spectrum of Compound 3. The **^{13}C-NMR spectrum** of **compound 3** displayed a characteristic **carbonyl (C=O) signal at δ 167.62 ppm**, confirming the presence of the **carbonyl functional group**. Other chemical shifts correspond to aromatic carbons, aliphatic carbons, and substituted groups, supporting the molecular structure of the compound.

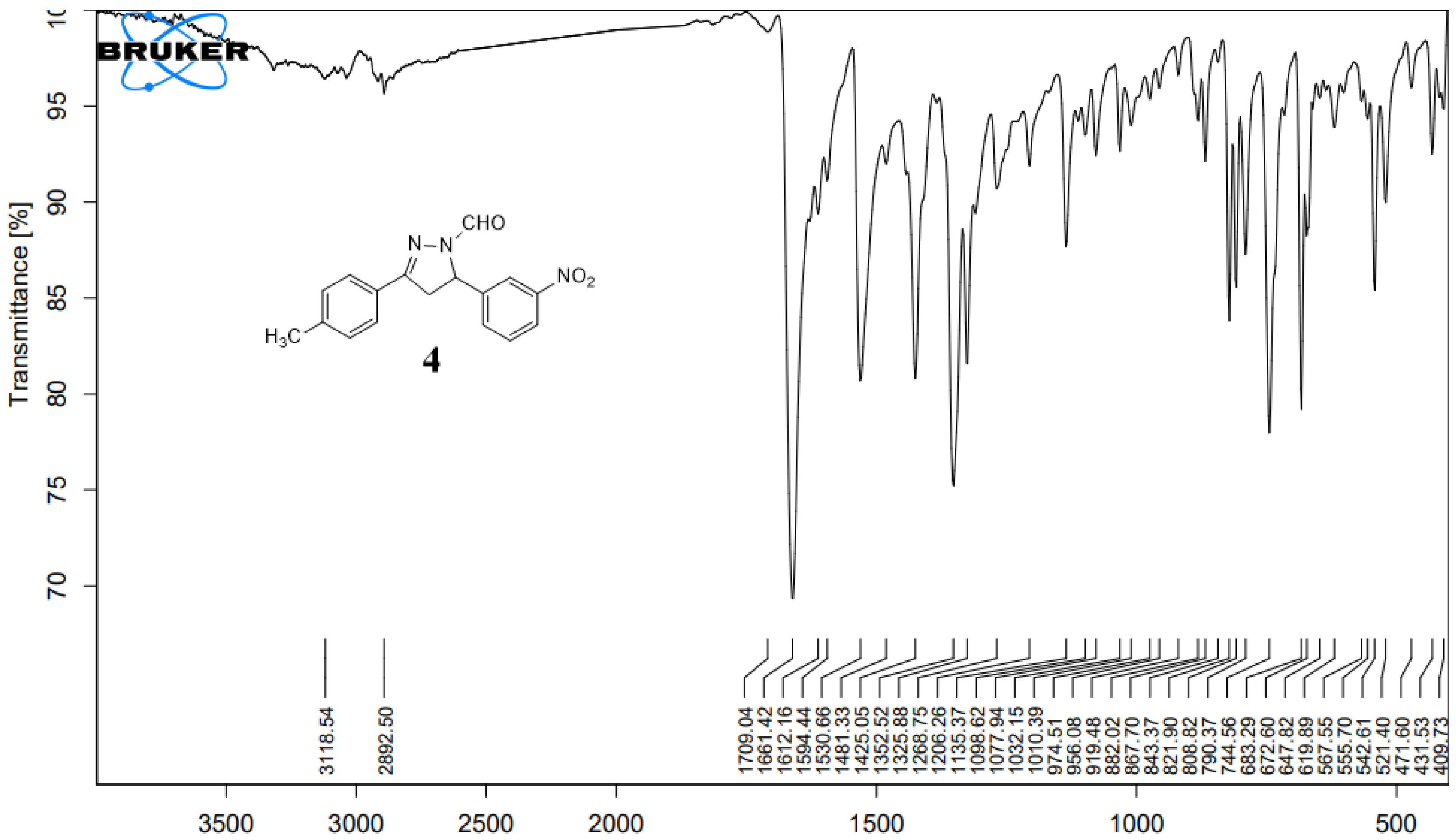

S7: FT-IR Spectrum of Compound 4. The FT-IR spectrum of compound **4** exhibited a strong absorption band at **1709.04 cm⁻¹**, which corresponds to the carbonyl (C=O) stretching vibration of the aldehyde functional group. Additionally, characteristic bands for C-H stretching were observed at **3118.54 cm⁻¹** (aromatic C-H) and **2892.50 cm⁻¹** (aliphatic C-H).

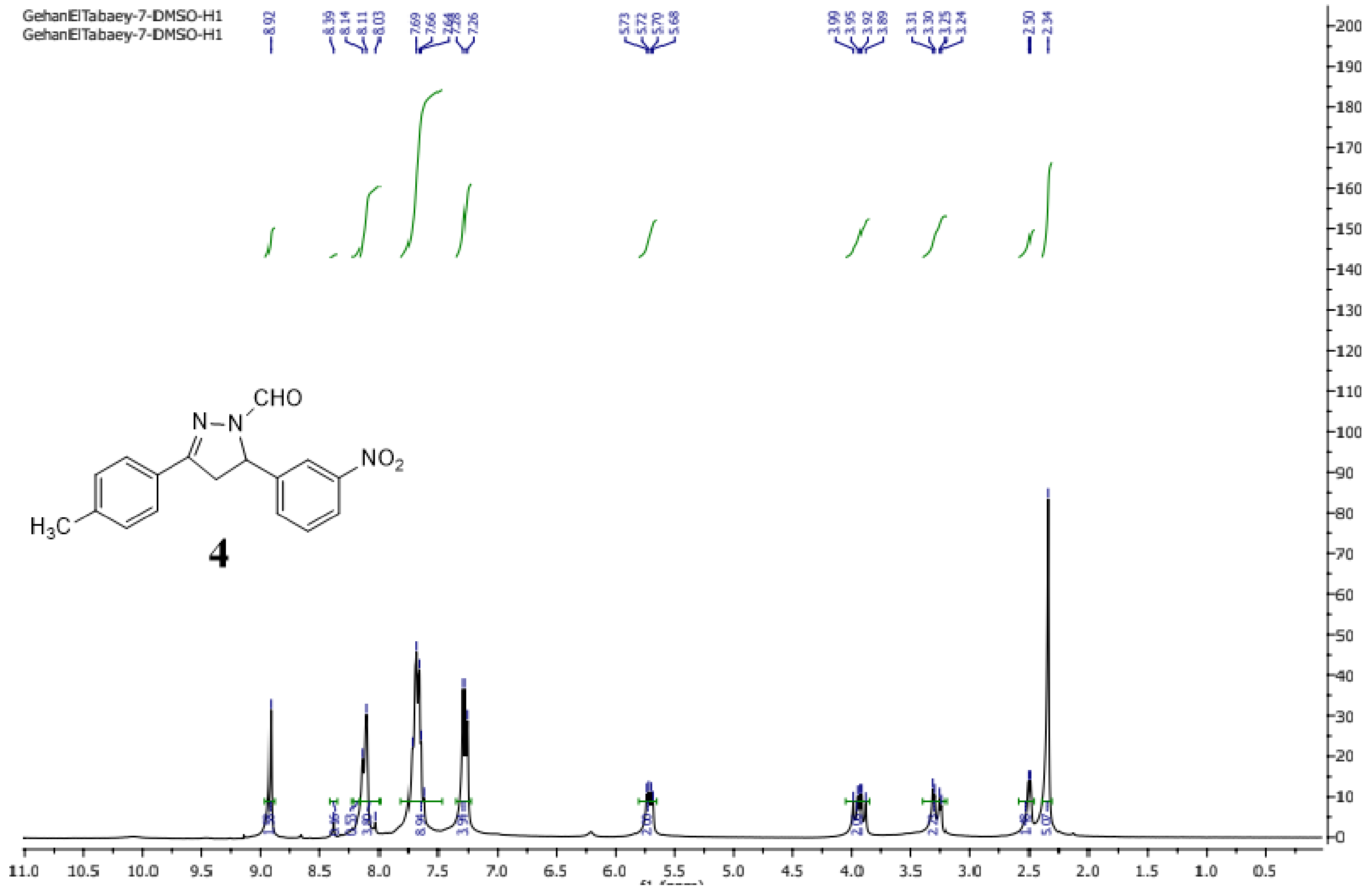

S8: **^{1}H NMR Spectrum of Compound 4**. The ^{1}H NMR spectrum of compound **4** exhibited a singlet at **δ 2.50 ppm** for **CH_3**, **CH_2** signals at **δ 3.89–3.99 ppm**, **CH** signals at **δ 5.68–5.73 ppm**, and an **aldehyde (-CHO) proton** at **δ 8.92 ppm**, confirming its molecular structure.

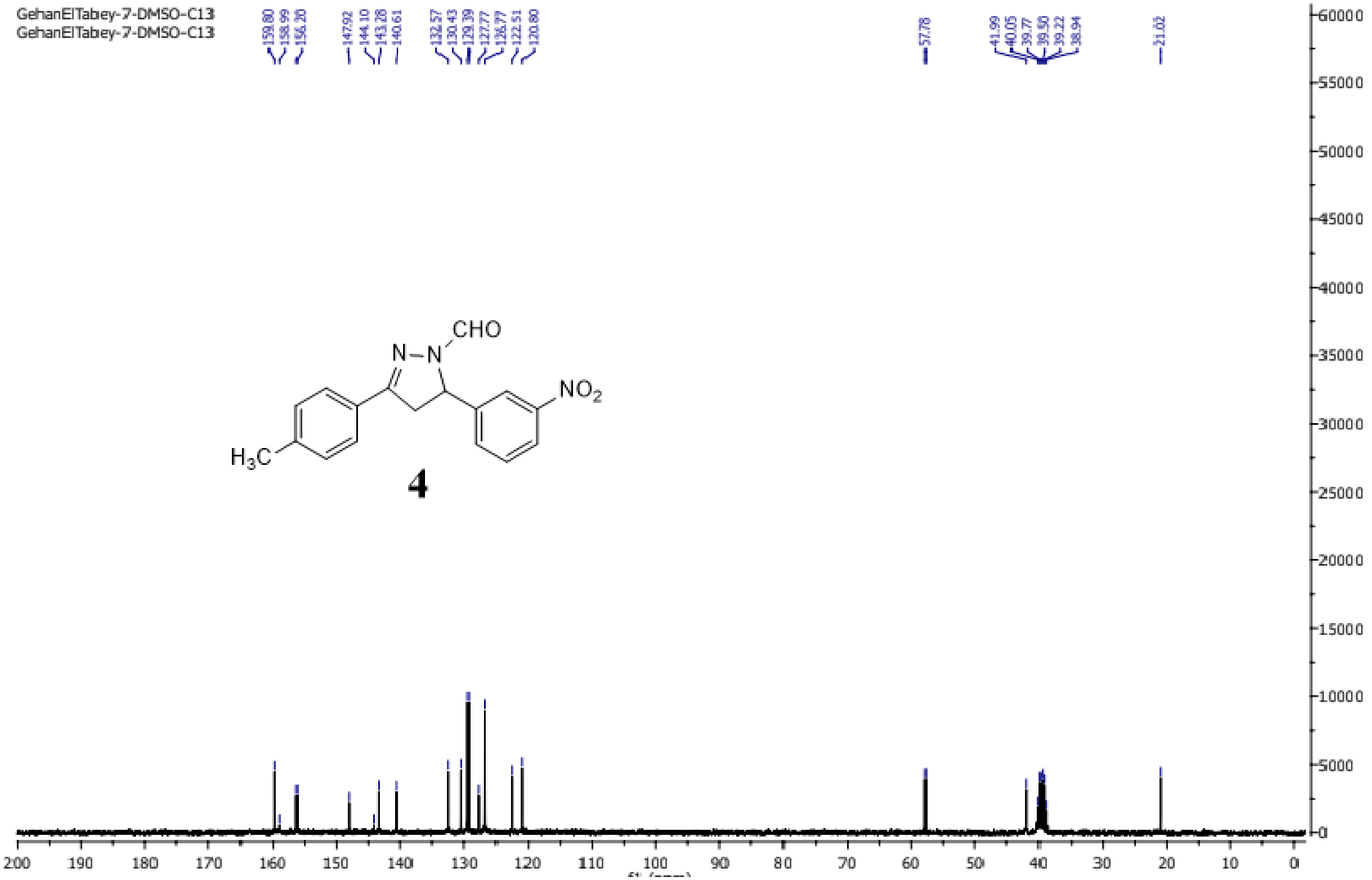

S9: ^{13}C NMR Spectrum of Compound 4.^{13}C NMR spectrum of compound **4** displayed signals in the low field region at **δ 156.20–159.80 ppm**, which were attributed to the **carbonyl (-C=O) group**, confirming the structural composition.

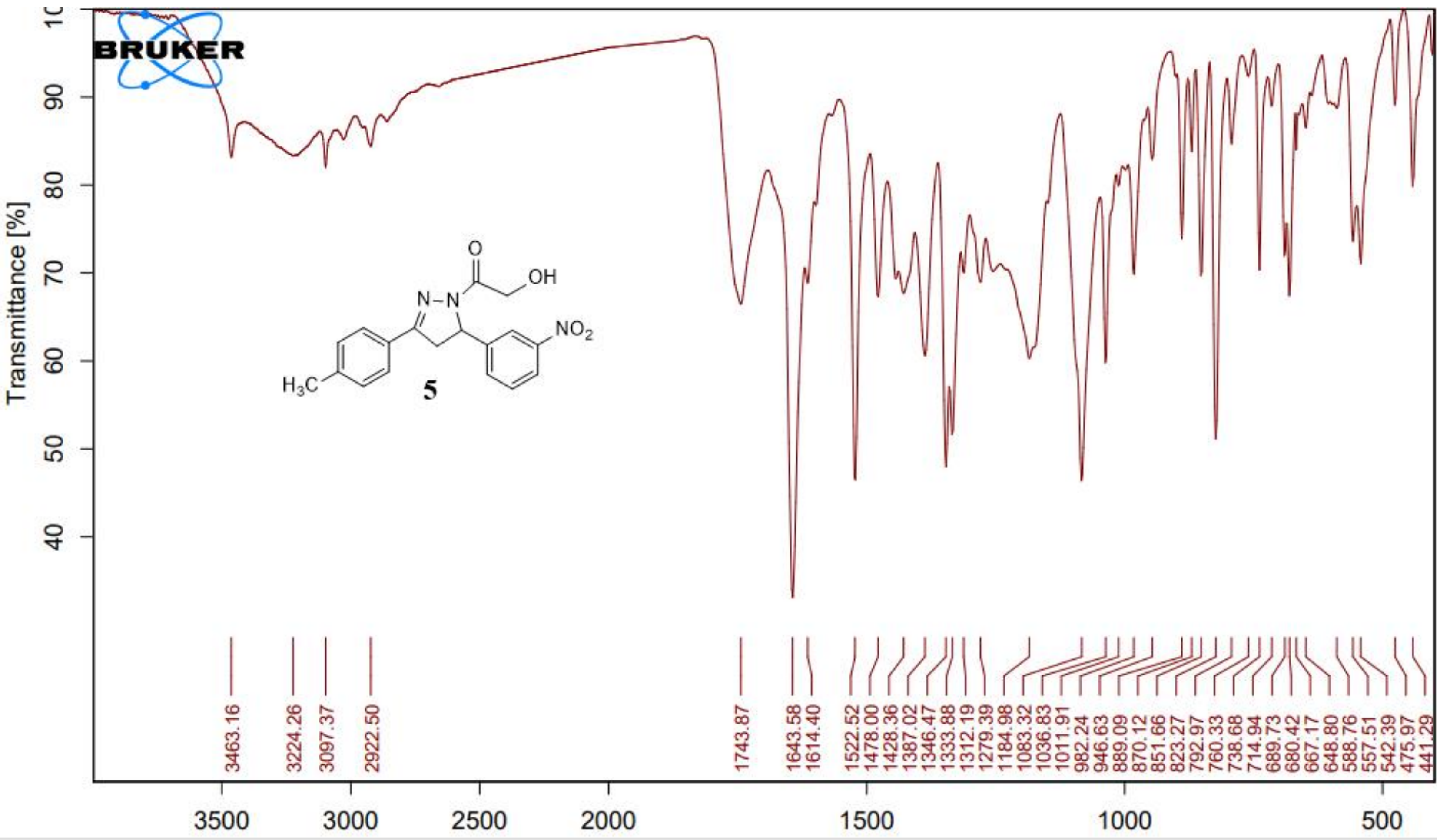

S10: **FT-IR Spectrum of Compound 5**. The FT-IR spectrum of compound **5** showed a broad band at **3224.26–3463.16 cm⁻¹** indicating the presence of an **OH** group, along with characteristic peaks at **1743.87 cm⁻¹ (C=O)** and **1643.58 cm⁻¹ (-C=N)** confirming the functional groups.

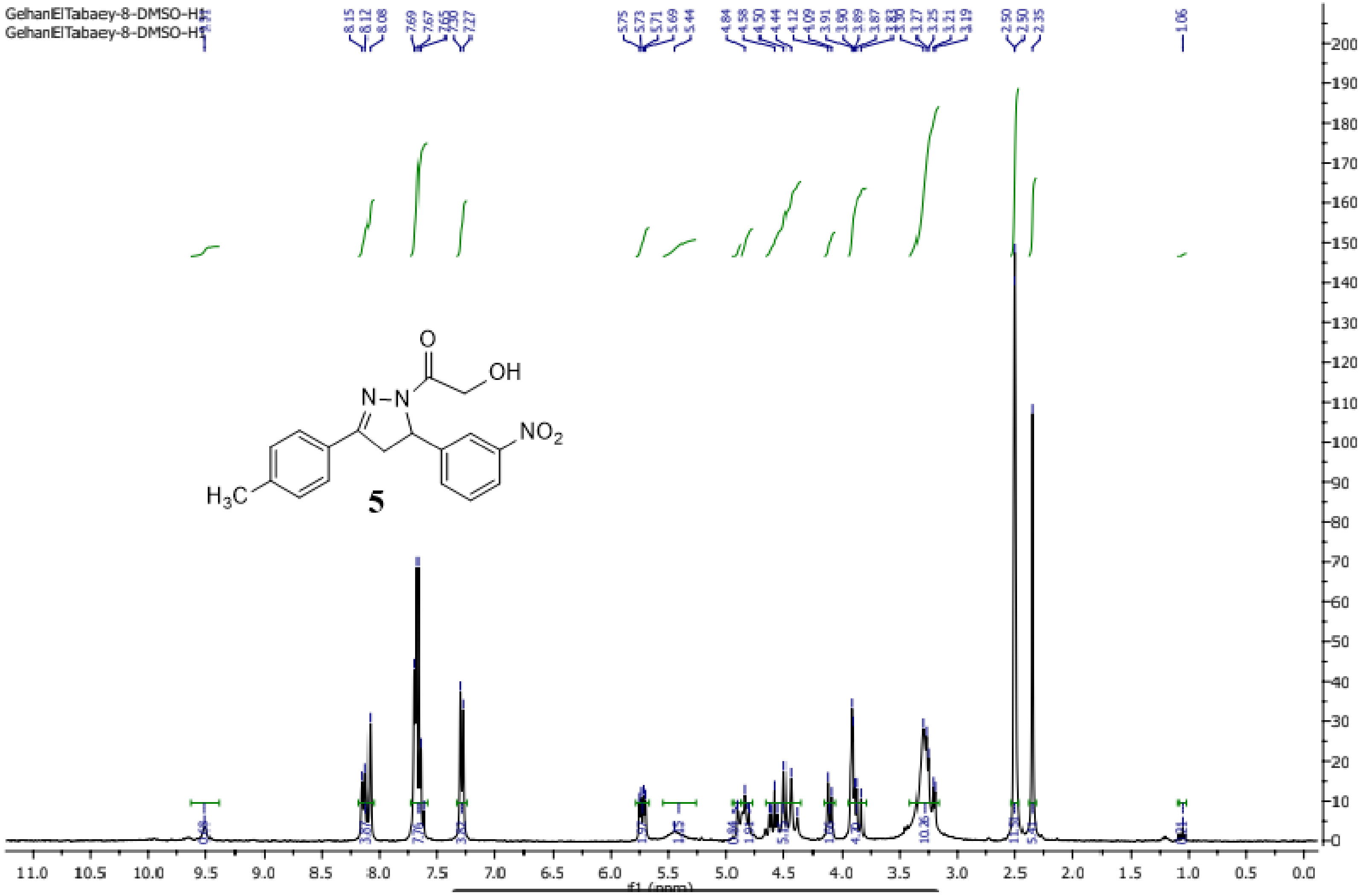

S11: **¹H NMR Spectrum of Compound 5.** The ¹H NMR spectrum of compound **5** in DMSO-d_6 displayed characteristic signals, including CH and CH_2 signals of the pyrazoline ring at **δ 3.83–4.84 ppm** and **δ 5.69–5.75 ppm**, respectively, an OH proton at **δ 9.51 ppm**, and CH_2 protons of the acylated group at **δ 5.44 ppm**, along with aromatic proton signals in the **7.0–8.5 ppm** range.

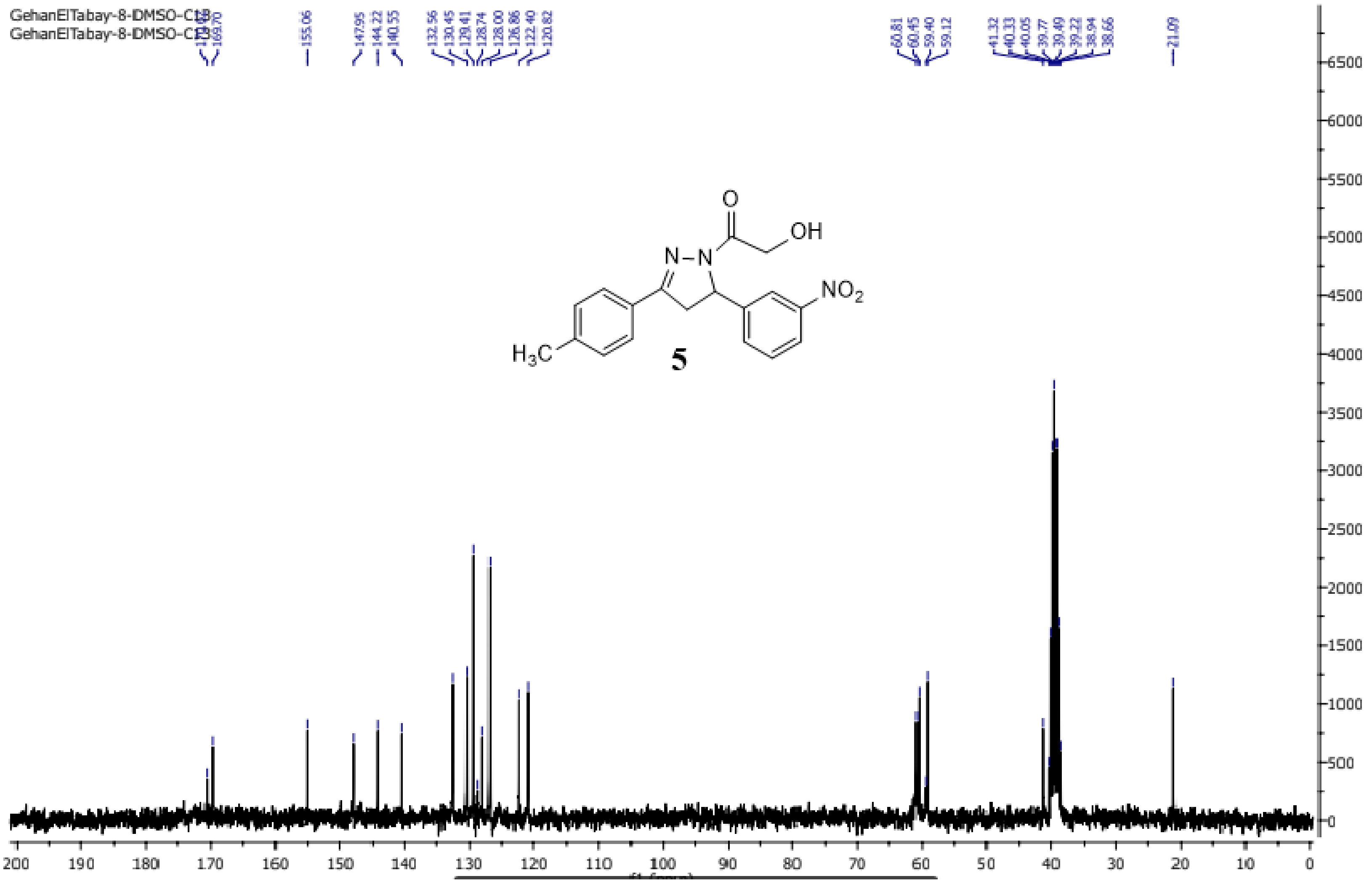

S12: **^{13}C NMR Spectrum of Compound 5.** The ^{13}C NMR spectrum of compound **5** displayed a characteristic signal at **169.70 ppm**, corresponding to the **sp^{2} carbonyl carbon**, along with multiple peaks in the aromatic and aliphatic regions.

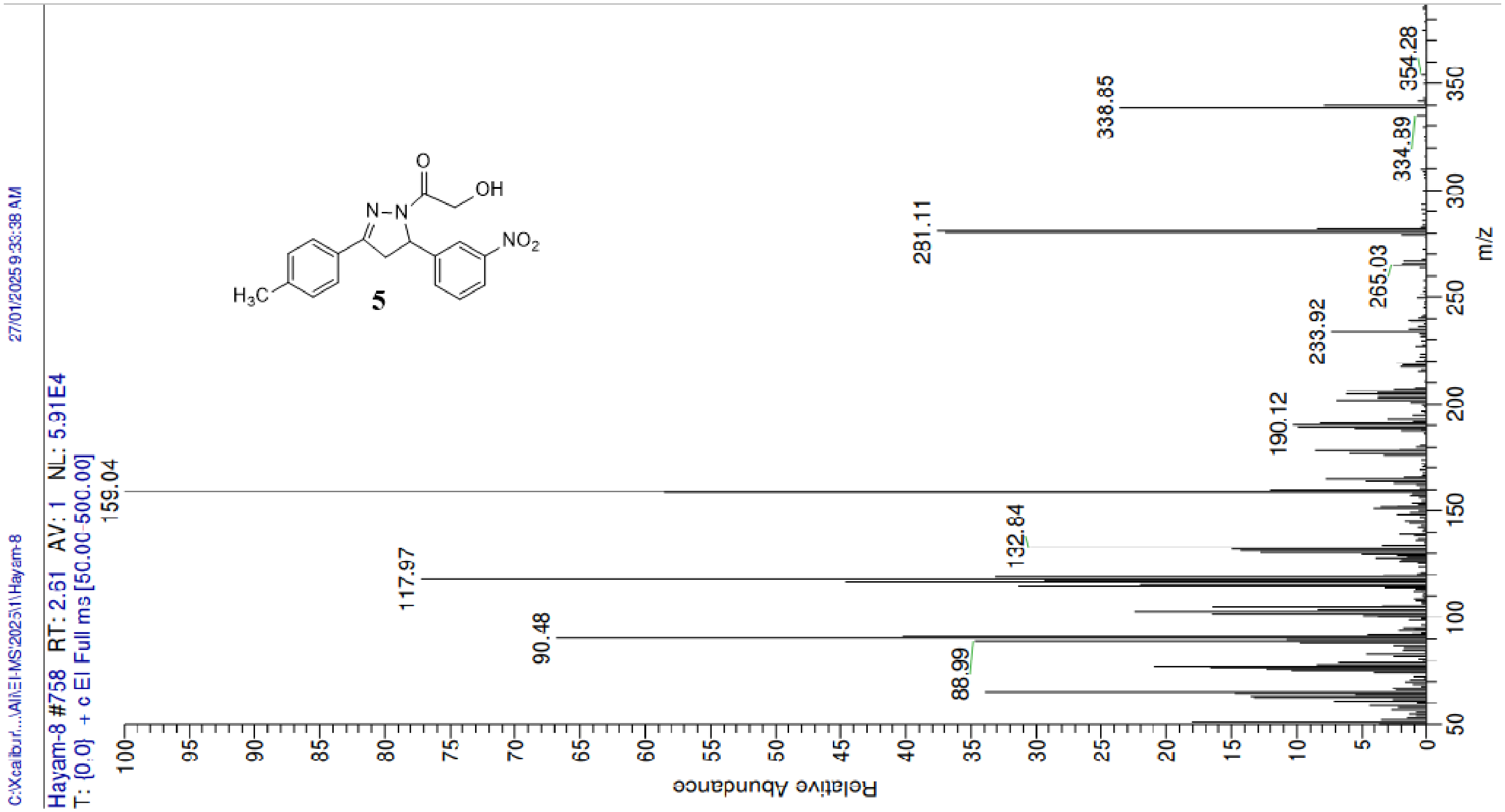

S13: **Mass Spectrum of Compound 5**. The mass spectrum of **compound 5** exhibits a molecular ion peak at **m/z 339.12 (M^+, 9%)**, indicating the molecular weight. The base peak appears at **m/z 159.04 (100%)**, representing the most stable and abundant fragment.

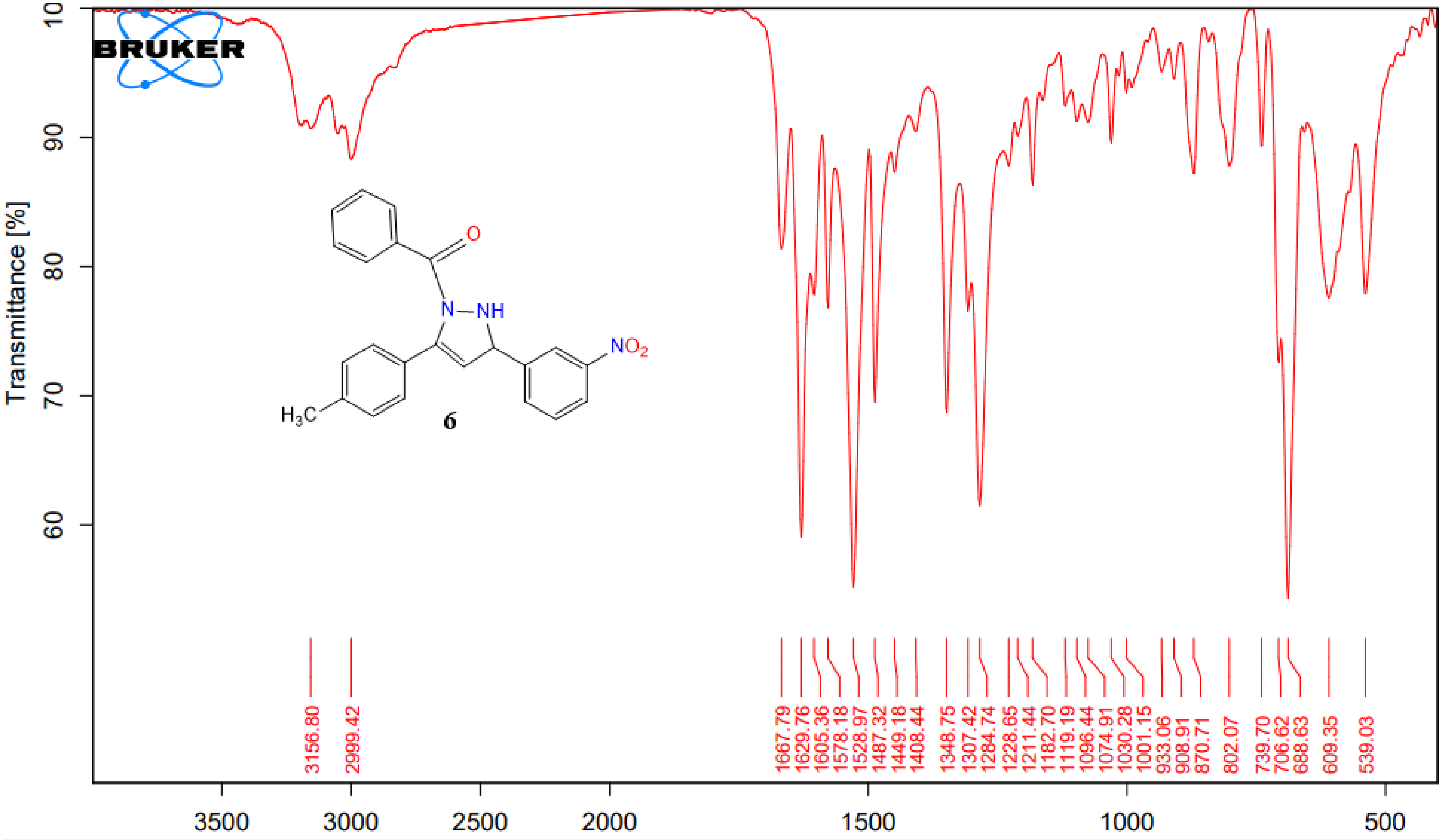

S14: FT-IR Spectrum of Compound 6. The FT-IR spectrum of **compound 6** reveals characteristic absorption bands at **3156.80 cm⁻¹** for the **N-H stretching vibration** and **1667.79 cm⁻¹** for the **C=O stretching vibration**.

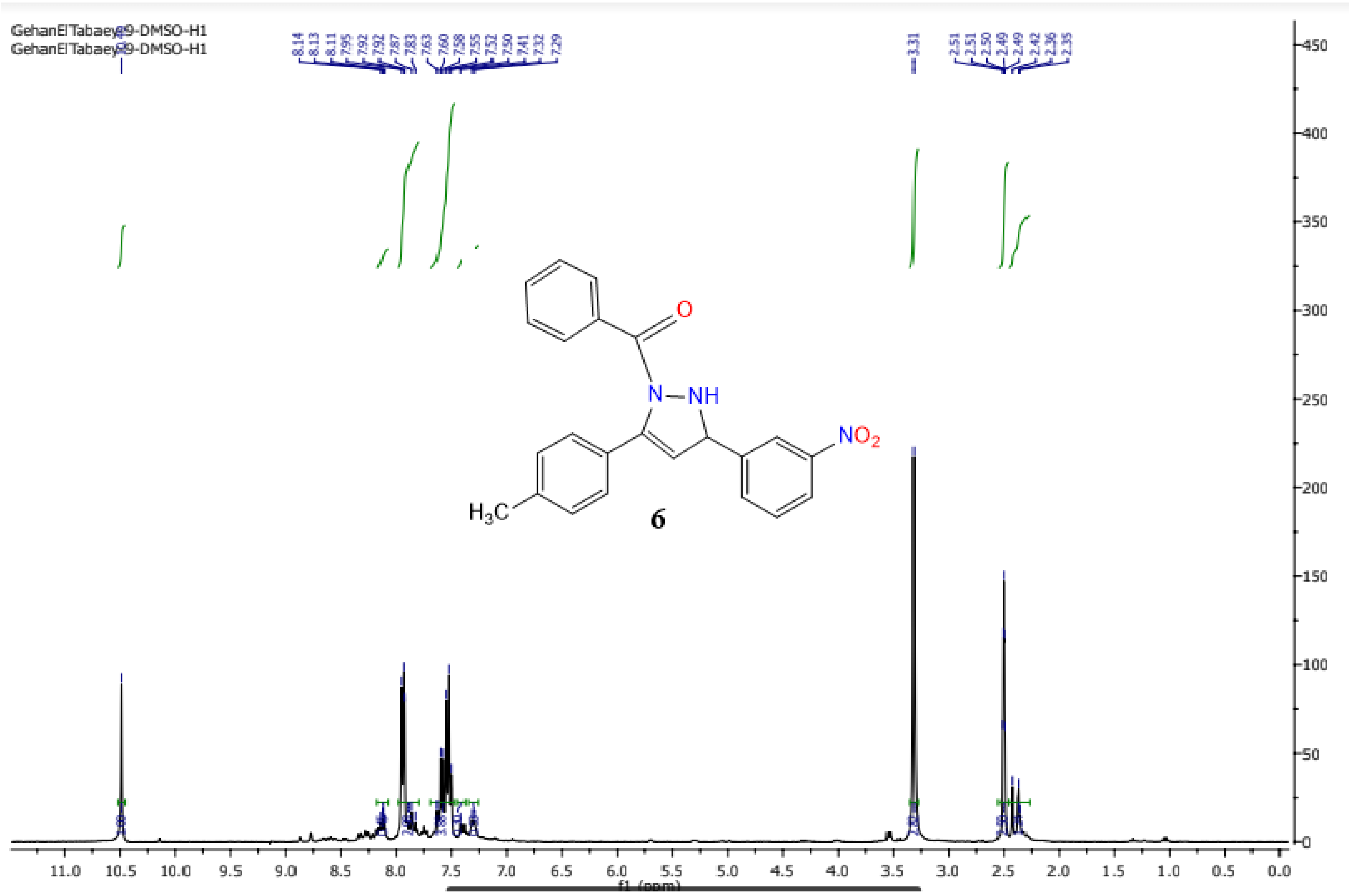

S15: [1]**H-NMR Spectrum of Compound 6**. The [1]**H-NMR spectrum** of **compound 6** confirms the structure with characteristic peaks. The **pyrazoline CH protons** appear at **δ 2.35-2.36 ppm** and **δ 3.56-3.57 ppm**, while the **NH proton** is observed at **δ 10.49 ppm**

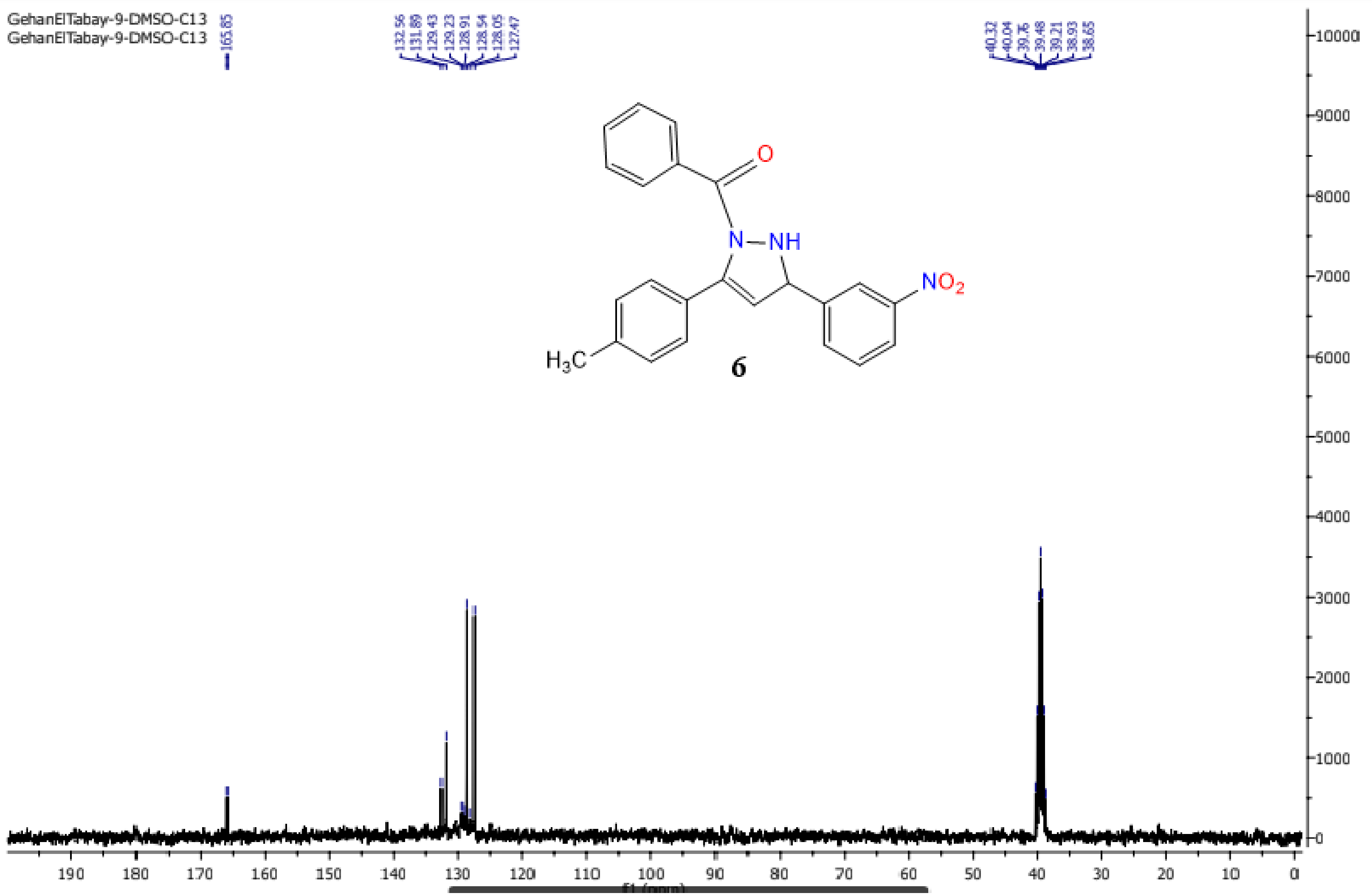

S16: **^{13}C-NMR Spectrum of Compound 6**. The **^{13}C-NMR spectrum** of **compound 6** shows multiple **aromatic carbon signals** in the region **δ 127.47-132.56 ppm**, confirming the presence of benzene rings. Additionally, the characteristic **carbonyl (C=O) carbon** signal appears at **δ 165.85 ppm**

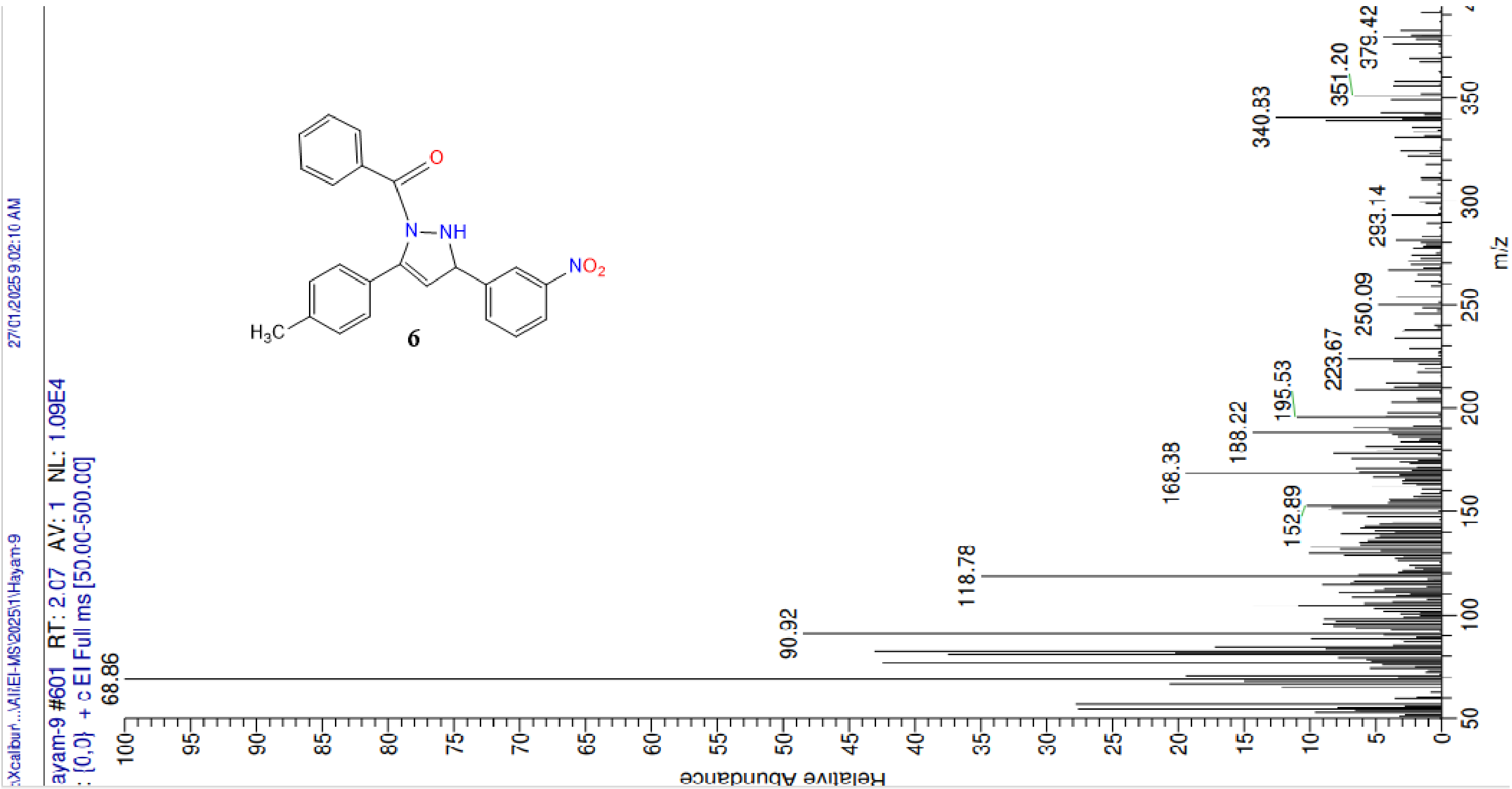

S17: Mass Spectrum (MS) of Compound 6. The **mass spectrum** of **compound 6** shows a **molecular ion peak (M^+) at m/z 385.14**, confirming the molecular weight. The **base peak at m/z 68.86** indicates the most abundant fragment.

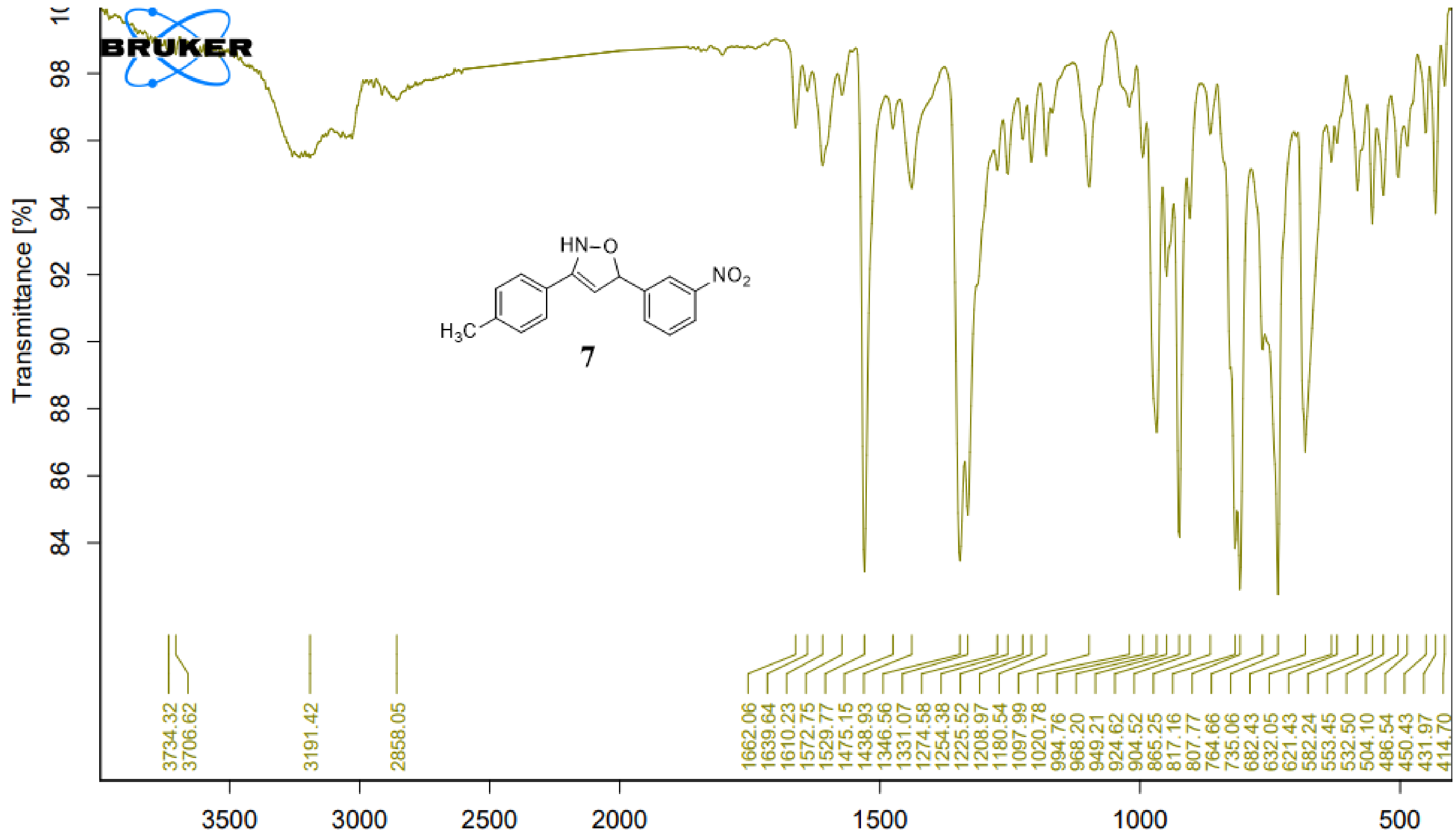

S18: FT-IR Spectrum of Compound 7. The **FT-IR spectrum** of **compound 7** shows characteristic absorption bands at **1662.06 cm^{-1}** for the **C=C stretching vibration** and at **3191.42 cm^{-1}** for the **N-H stretching vibration**.

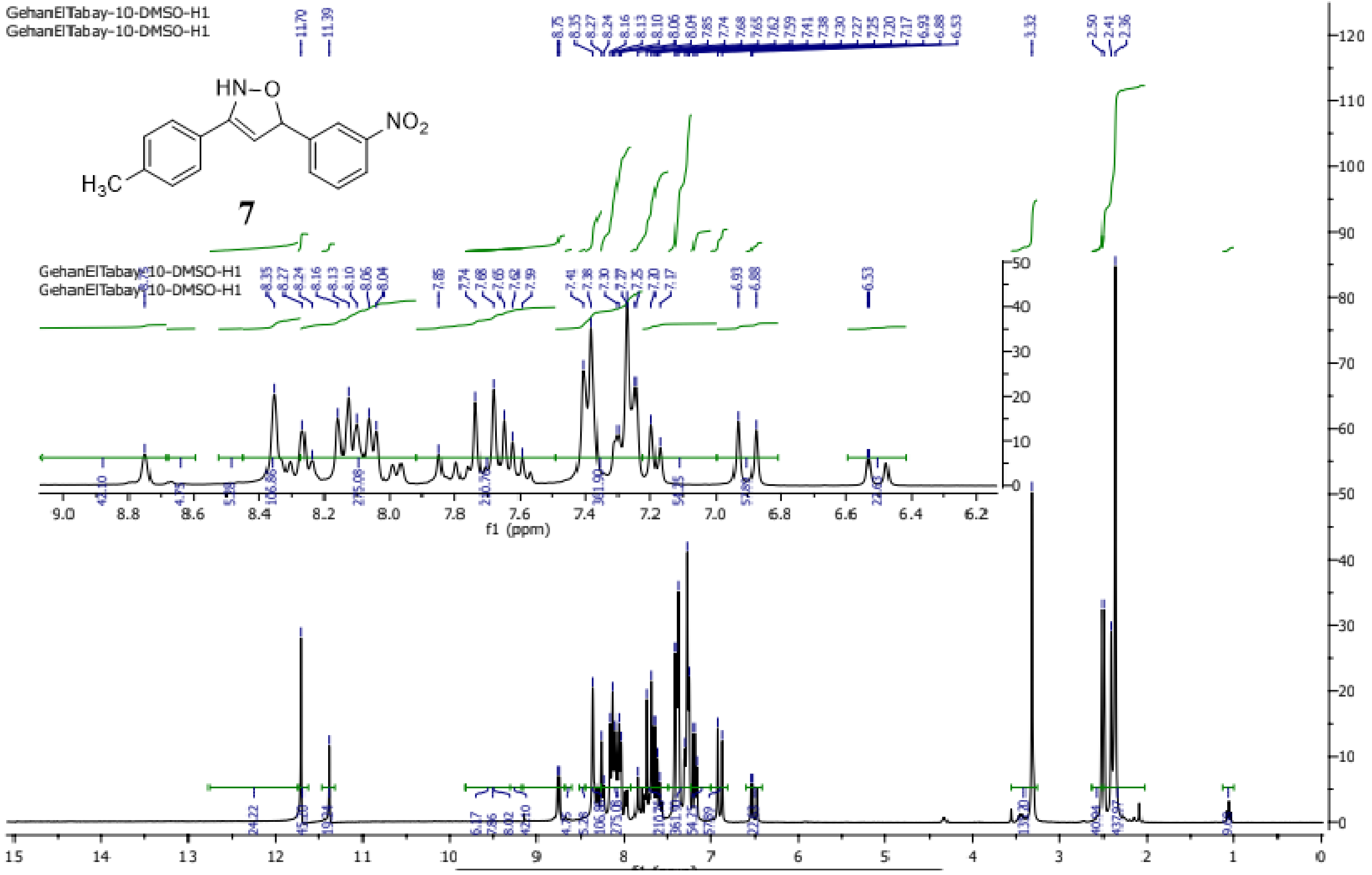

S19: **¹H-NMR Spectrum of Compound 7**. The **¹H-NMR spectrum** of **compound 7** shows a characteristic **singlet at δ 11.70 ppm**, which is assigned to the **NH proton**.

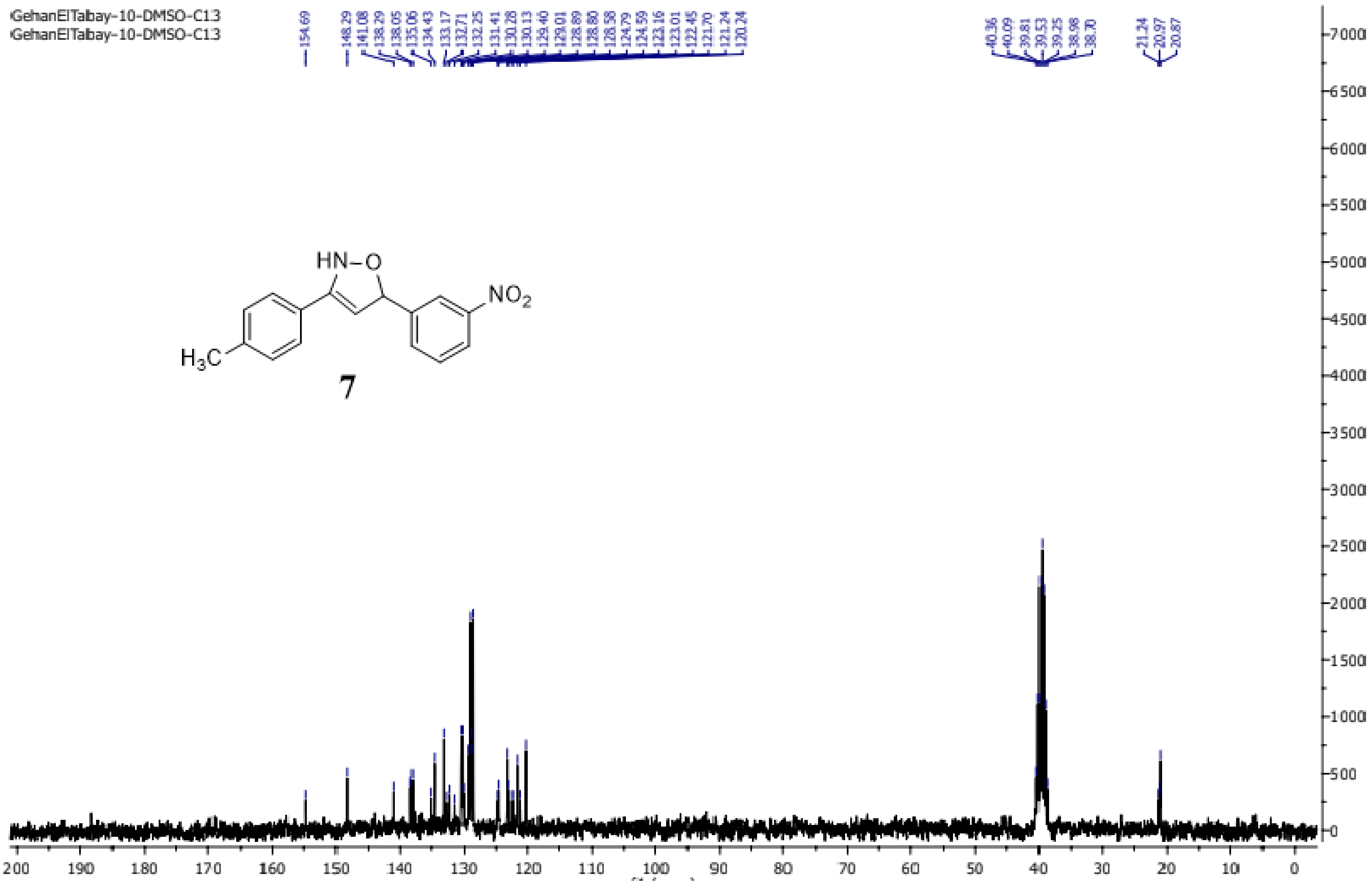

S20: **^{13}C NMR Spectrum of Compound 7.** The ^{13}C NMR spectrum of compound **7** displayed a characteristic signal at **169.70 ppm**, corresponding to the **sp² carbonyl carbon**, along with multiple peaks in the aromatic and aliphatic regions,

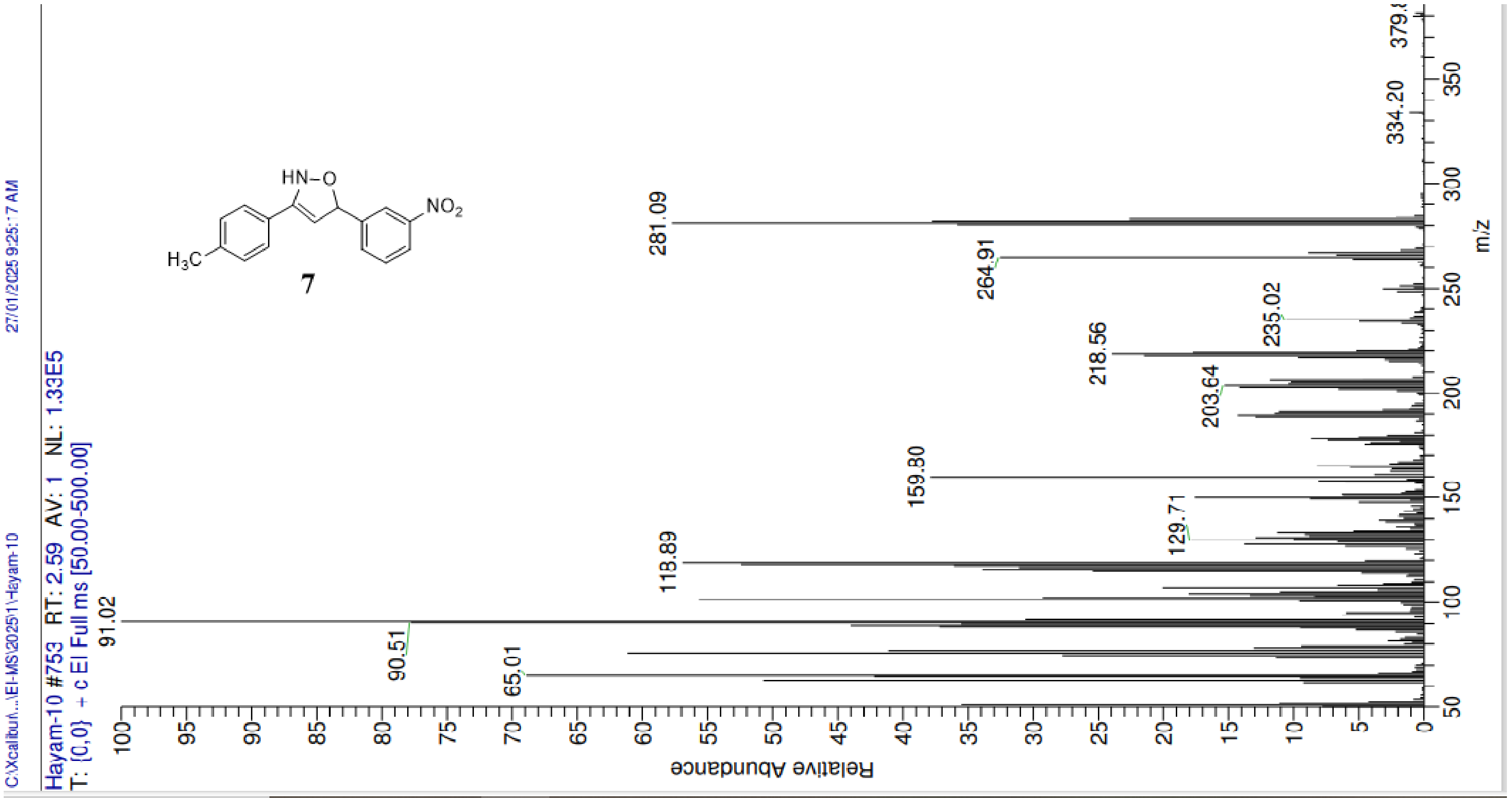

S21: **Mass Spectrum of Compound 7**. The **MS spectrum** of **compound 7** displays a **molecular ion peak (M^+) at m/z 280.10**, confirming its molecular weight. The **base peak at m/z 91.02** suggests a stable fragment, indicative of a benzyl cation.

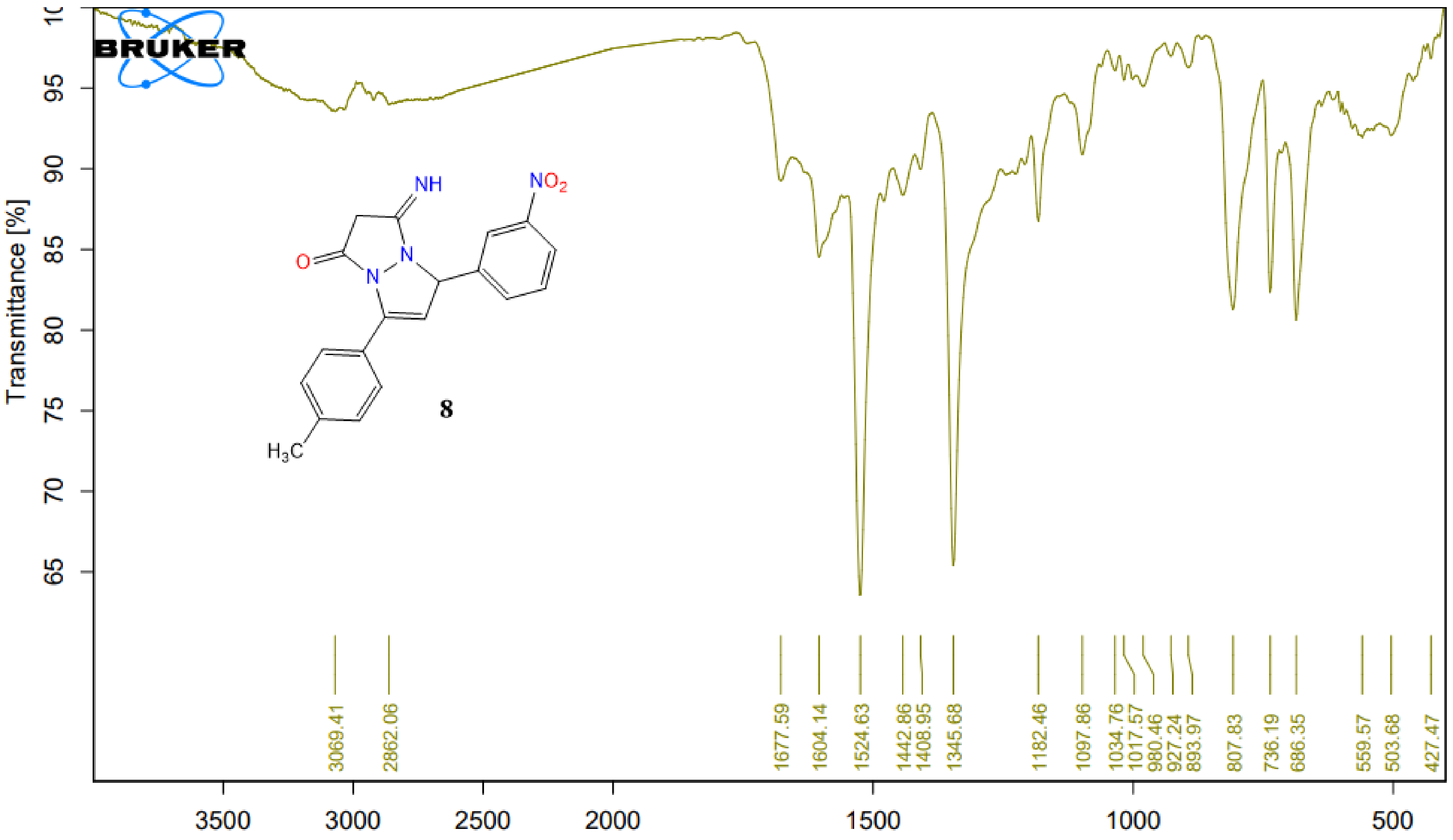

S22: IR Spectrum of Compound 8. The **IR spectrum** of **compound 8** shows characteristic absorption bands at **1677.59 cm⁻¹** for the **C=O (carbonyl) group** and **1604.14 cm⁻¹** for the **C=N (imine) bond.**

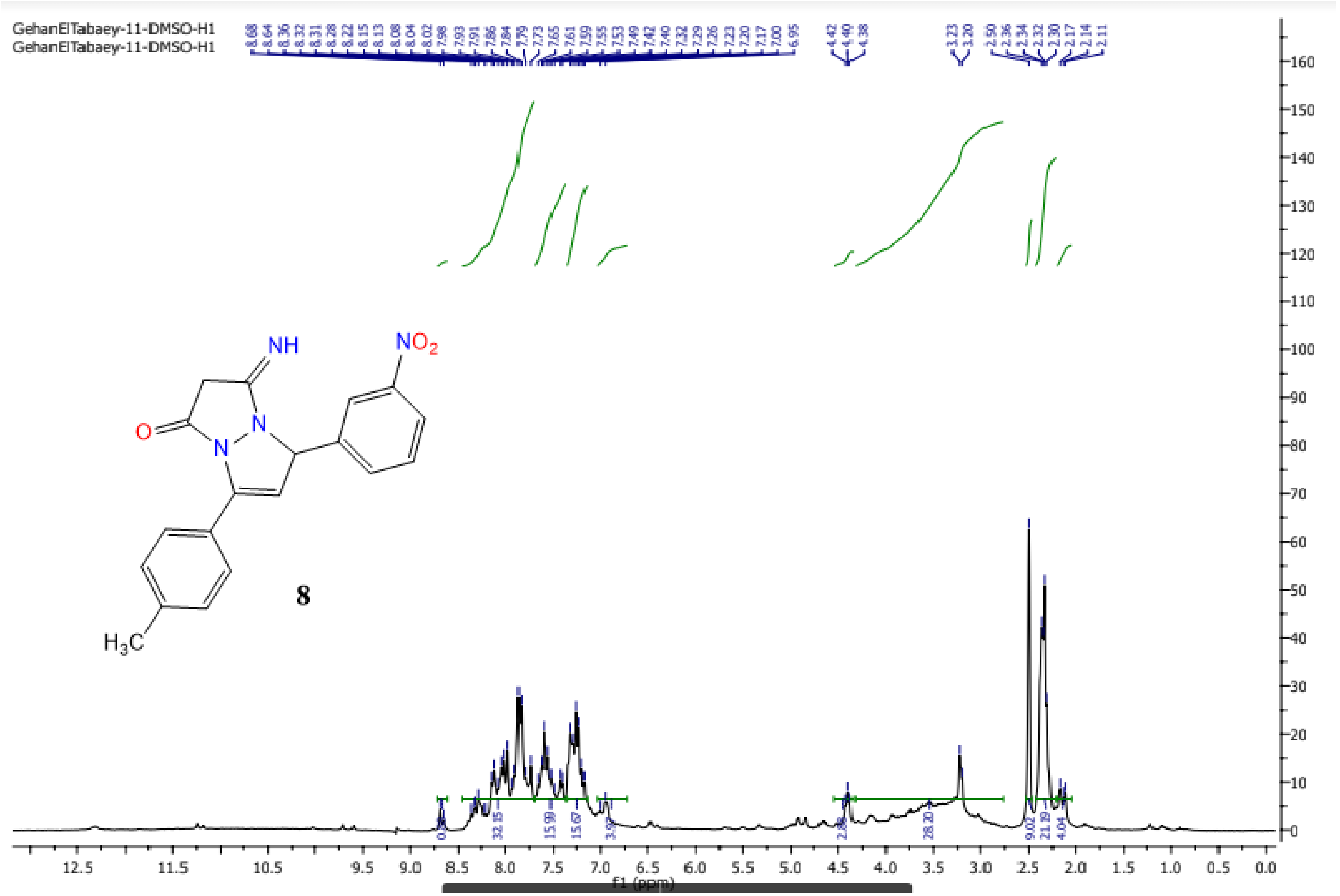

S23: **¹H-NMR Spectrum of Compound 8**. The **¹H-NMR spectrum** of **compound 8** displays two characteristic signals at **8.68 ppm**, corresponding to the **NH protons**, confirming the presence of amine functional groups.

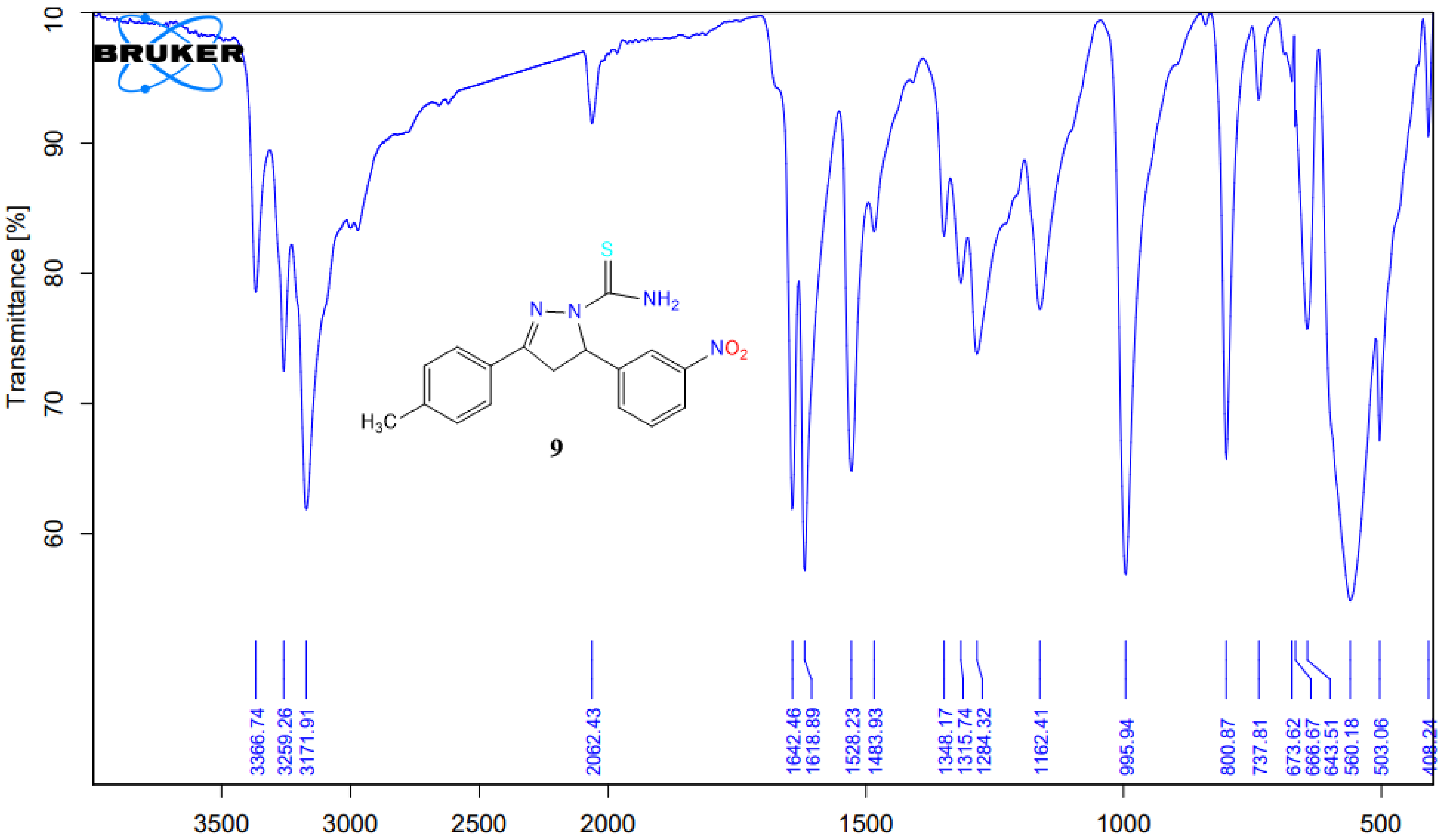

S24: IR Spectrum of Carbothioamide 9. The **IR spectrum** of **compound 9** confirms key functional groups: the **C=N** stretching band at **1642.46 cm^{-1}**, the **C=S** stretching band at **1284.32 cm^{-1}**, and the **NH_2** absorption band at **3259.26 cm^{-1}**.

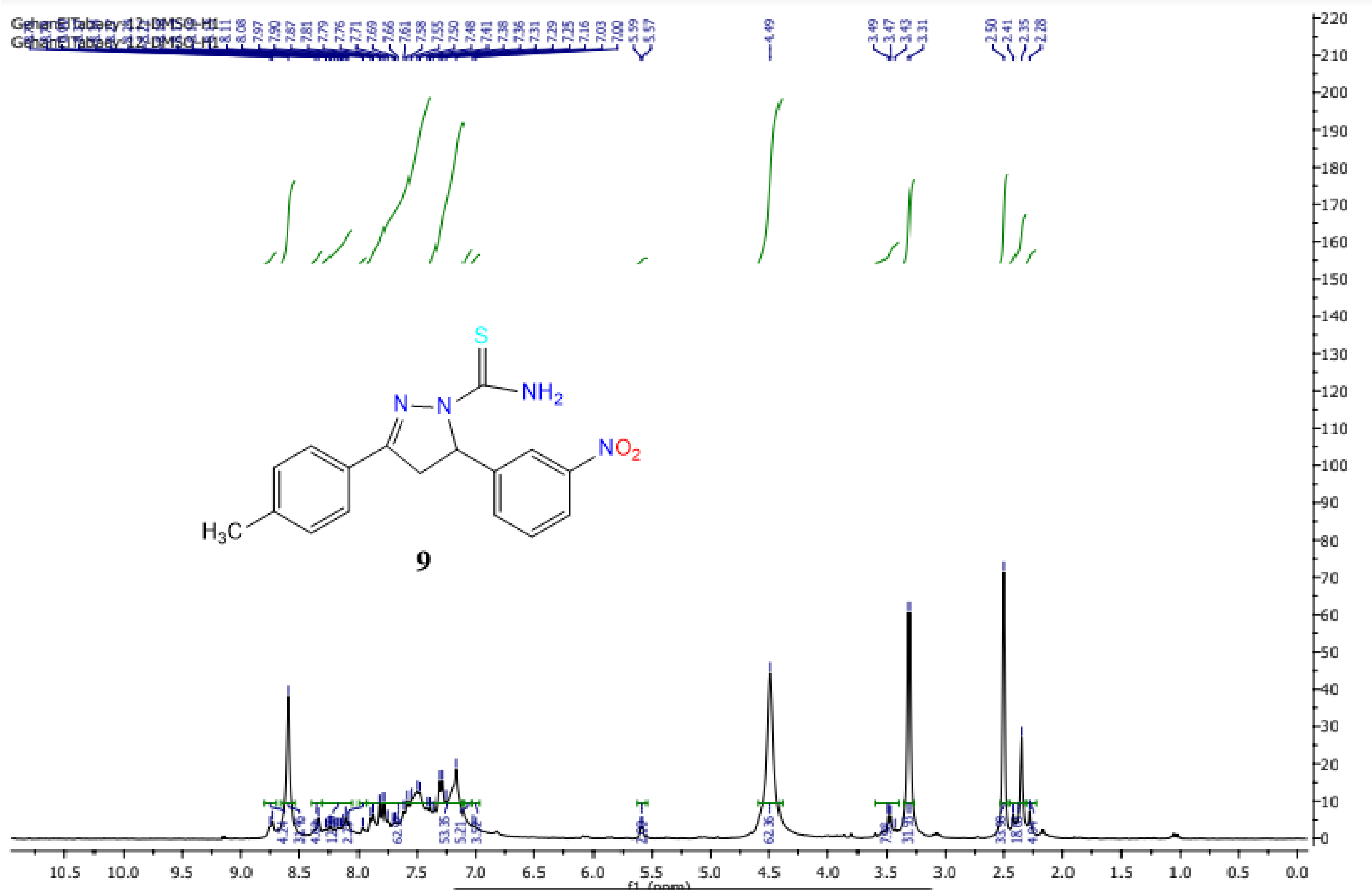

9

S25: **¹H-NMR Spectrum of Carbothioamide 9**. The **¹H-NMR spectrum** of **compound 9** displays characteristic signals of the pyrazoline ring: a **CH_2 multiplet** at **3.31–3.49 ppm**, a **CH singlet** at **5.57–5.59 ppm**, and the **NH proton** appearing as a distinct signal at **8.73 ppm**.

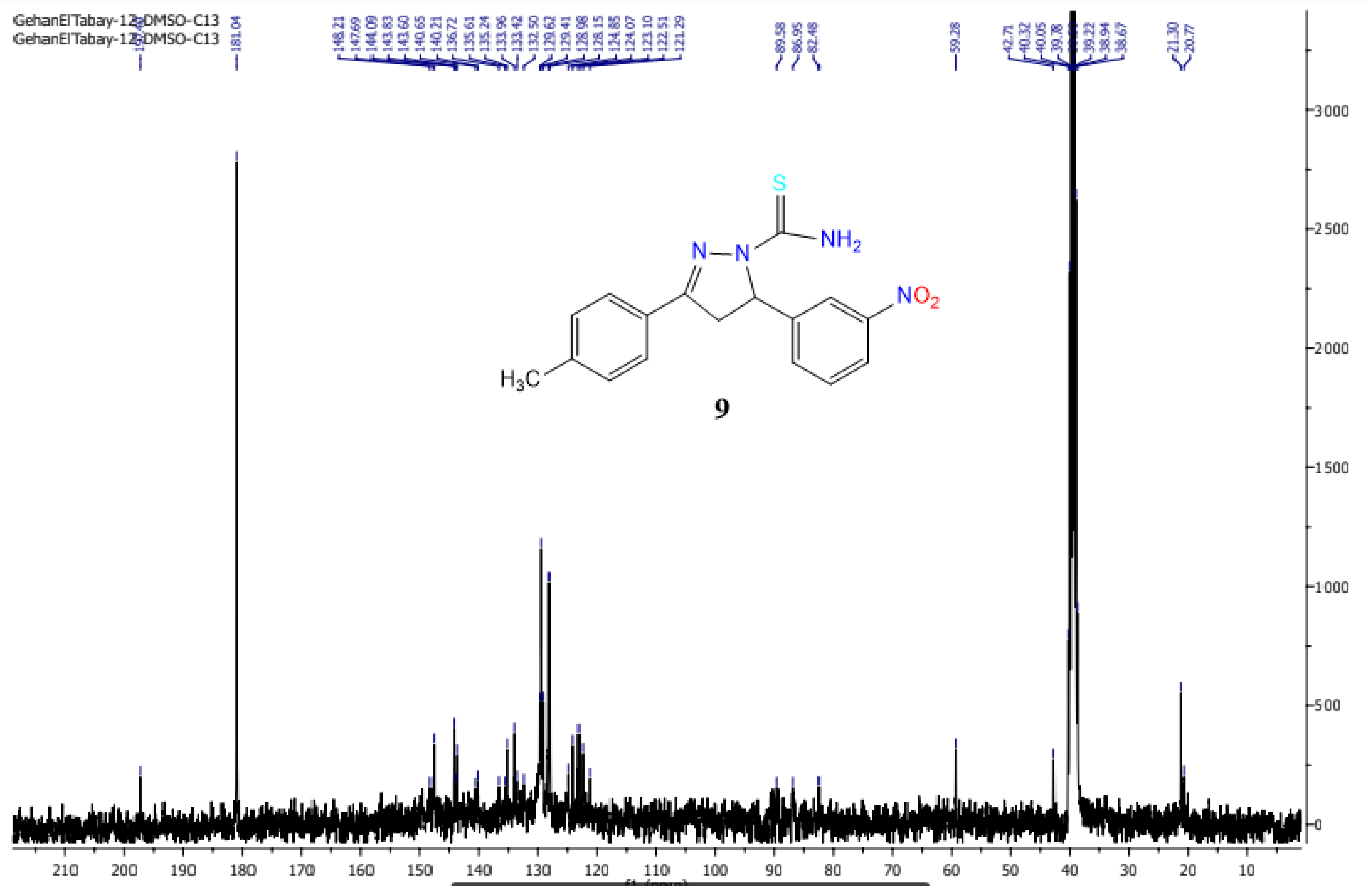

S26: **^{13}C-NMR Spectrum of Carbothioamide 9**. The **^{13}C-NMR spectrum** of **compound 9** displays a characteristic signal at **δ 181.04 ppm**, corresponding to the **C=S (thiocarbonyl) group**, confirming the presence of the carbothioamide functional group. Other signals in the spectrum represent aromatic and aliphatic carbons, supporting the molecular structure of the synthesized compound.

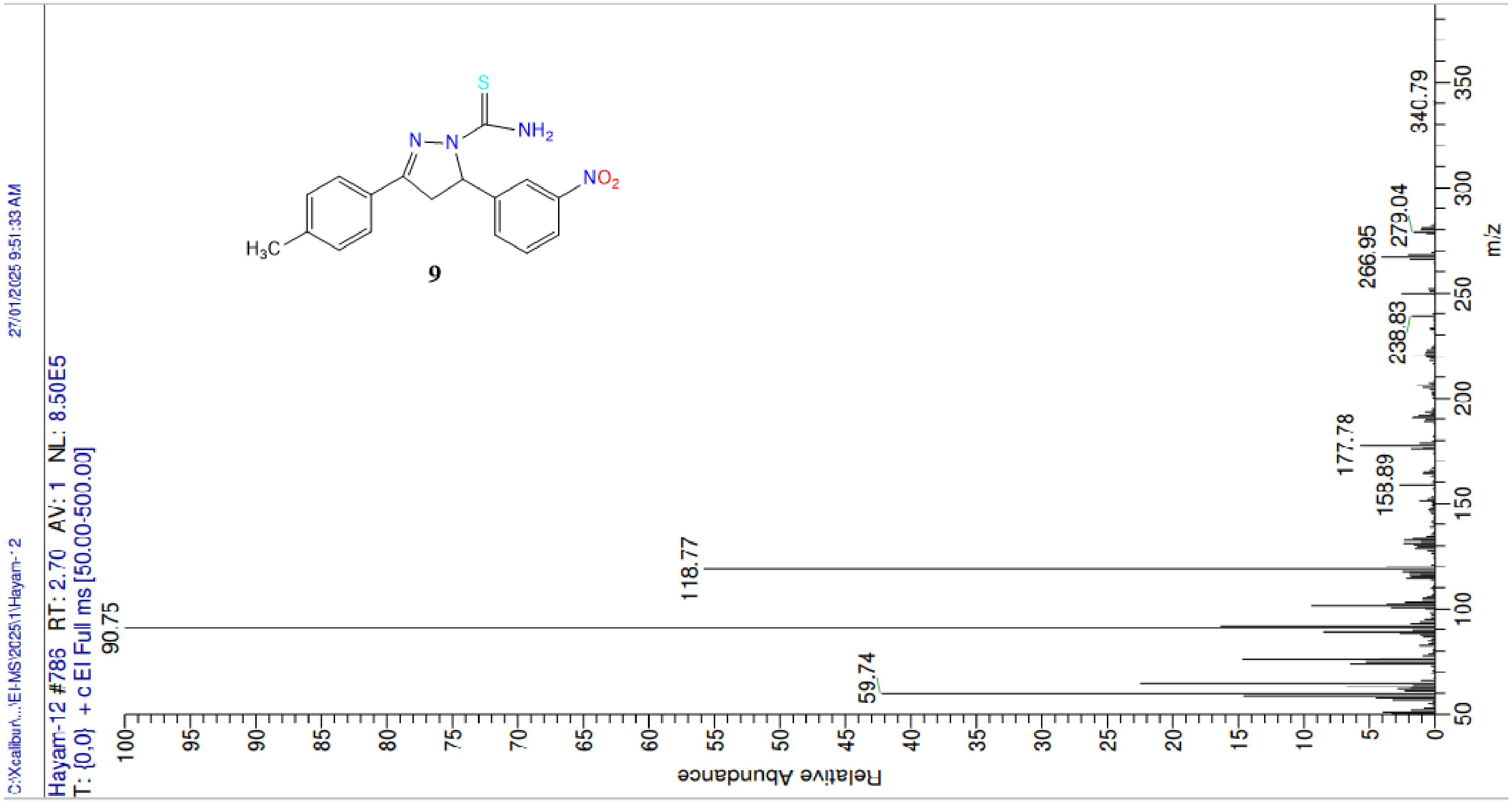

S27: **Mass Spectrum of Carbothioamide 9**. The **MS spectrum** of **compound 9** shows a molecular ion peak at **m/z 340.10 (M^+, 9%)**, confirming its molecular weight. The base peak at **m/z 90.75 (100%)** represents the most abundant fragment, indicating a stable cleavage pattern.

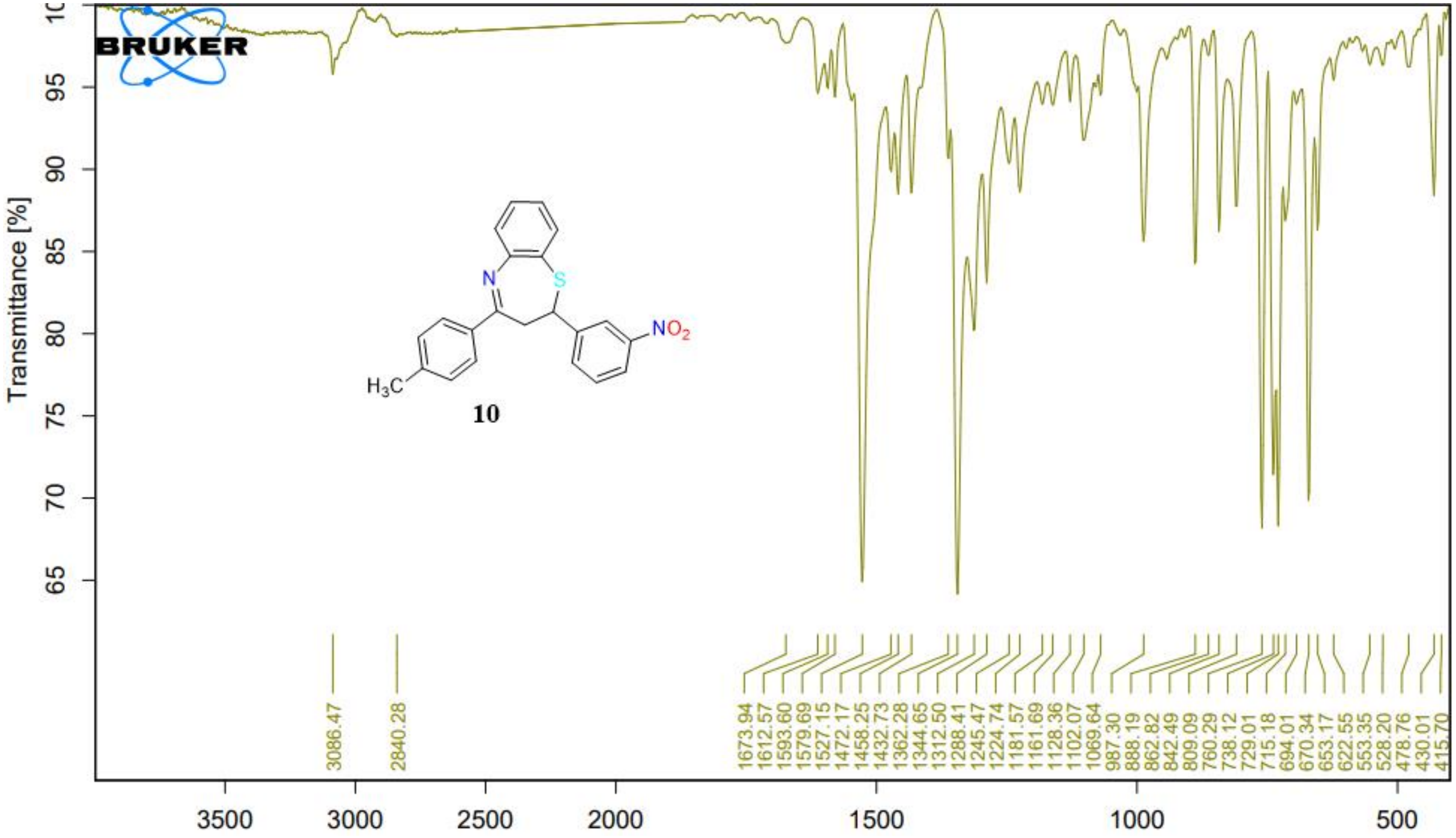

S28: IR Spectrum of Compound 10. The **IR spectrum** of **compound 10** exhibits a strong absorption band at **1673.94 cm⁻¹**, corresponding to the **C=N stretching vibration**. This confirms the presence of an imine (C=N) functional group in the molecular structure.

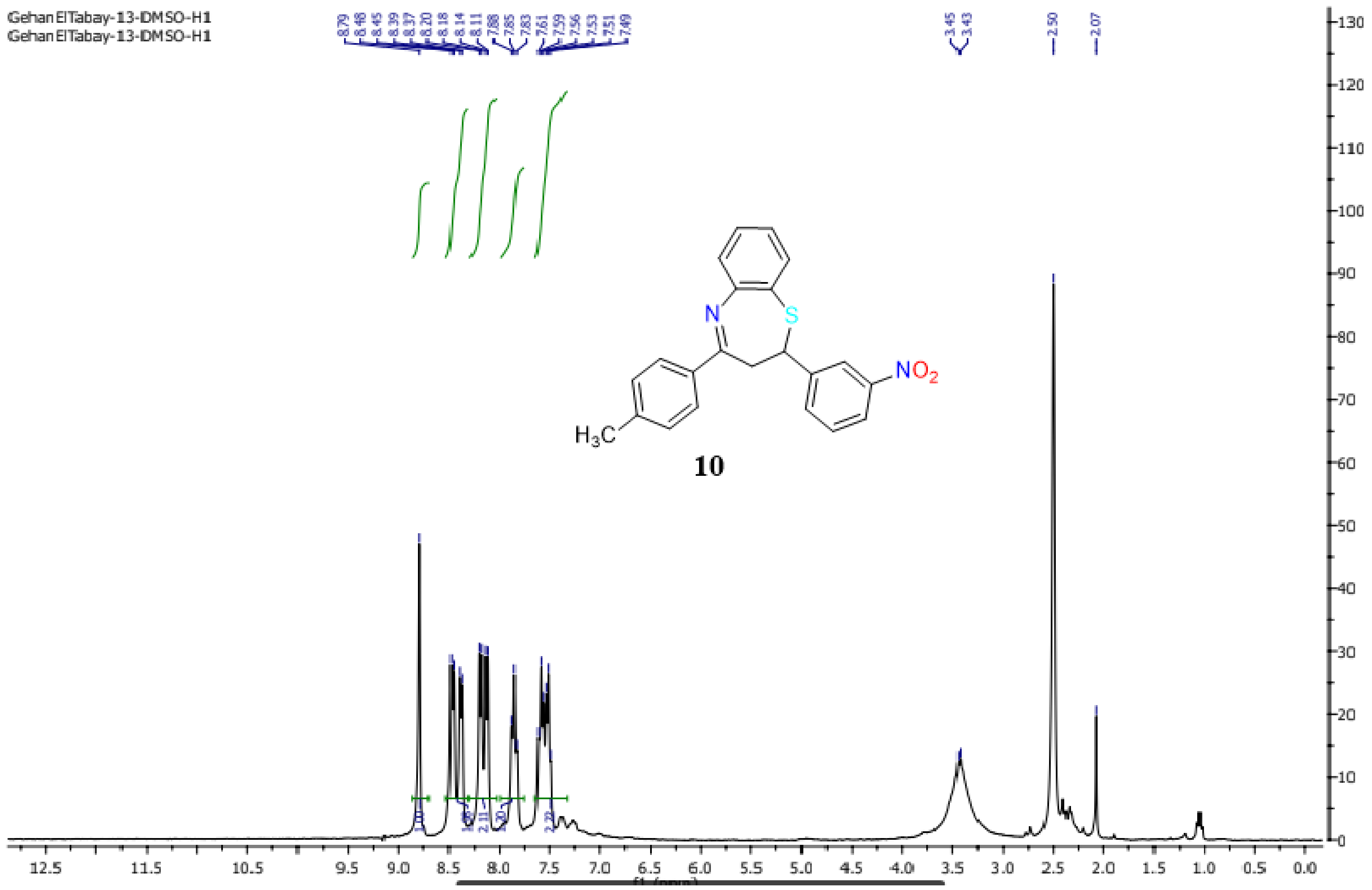

S29: [1]**H-NMR Spectrum of Compound 10**. In the [1]**H-NMR spectrum** of **compound 10**, a **multiplet signal** appears at **δ 3.43–3.45 ppm**, which corresponds to the **CH_2 protons of the thiazepine ring**. This confirms the presence of the thiazepine moiety in the molecular structure.

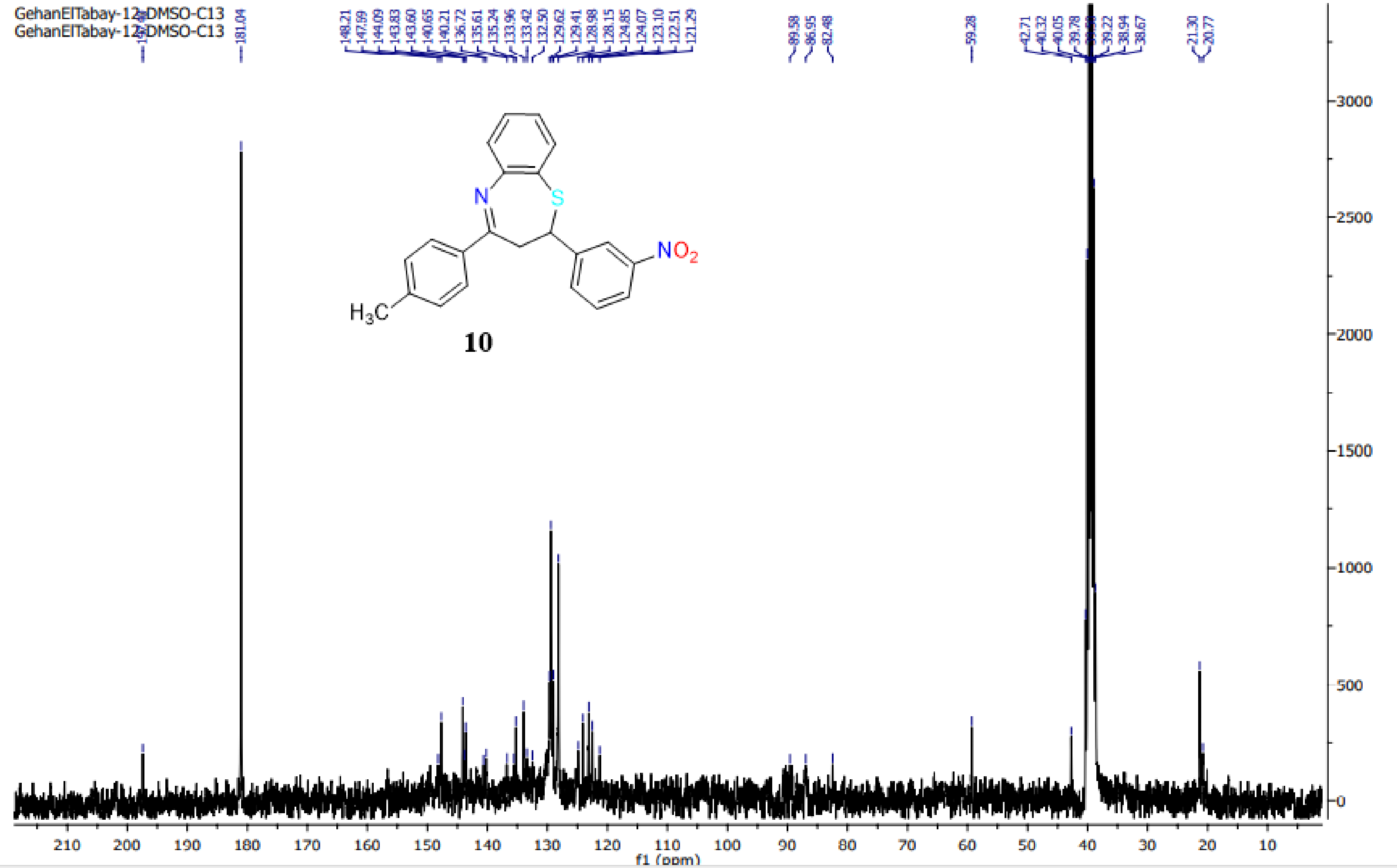

S30: ^{13}**C NMR Spectrum of Compound 10.** The ^{13}C NMR spectrum of compound **10** displayed a characteristic signal at **181.04 ppm**, corresponding to **C=N**, along with multiple peaks in the aromatic and aliphatic regions.

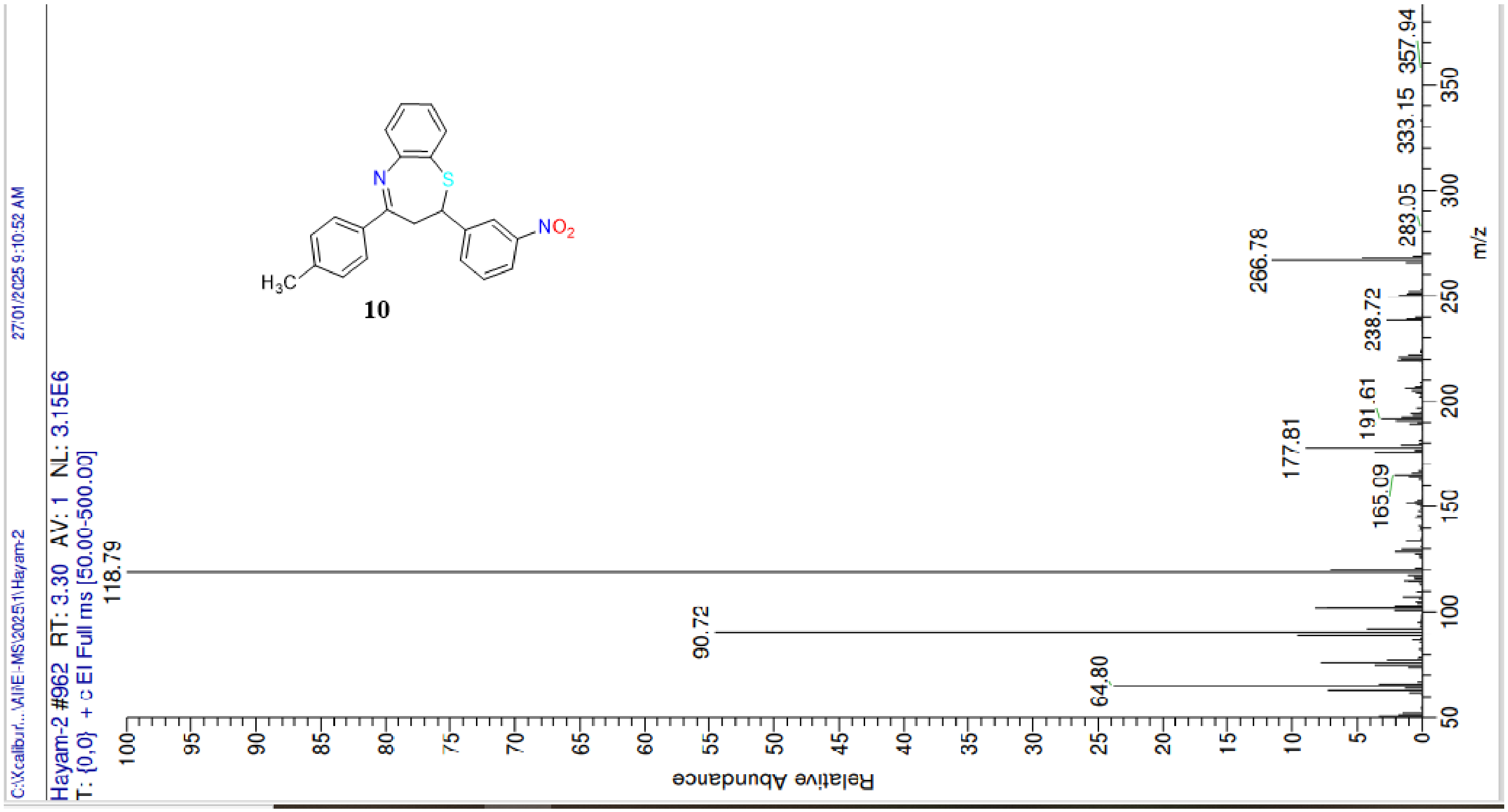

S31: **Mass Spectrum of Compound 10**. The **MS spectrum** of **compound 10** displays a **molecular ion peak (M^+) at m/z 374.11**, confirming its molecular weight. The **base peak at m/z 118.79** suggests a stable fragment.

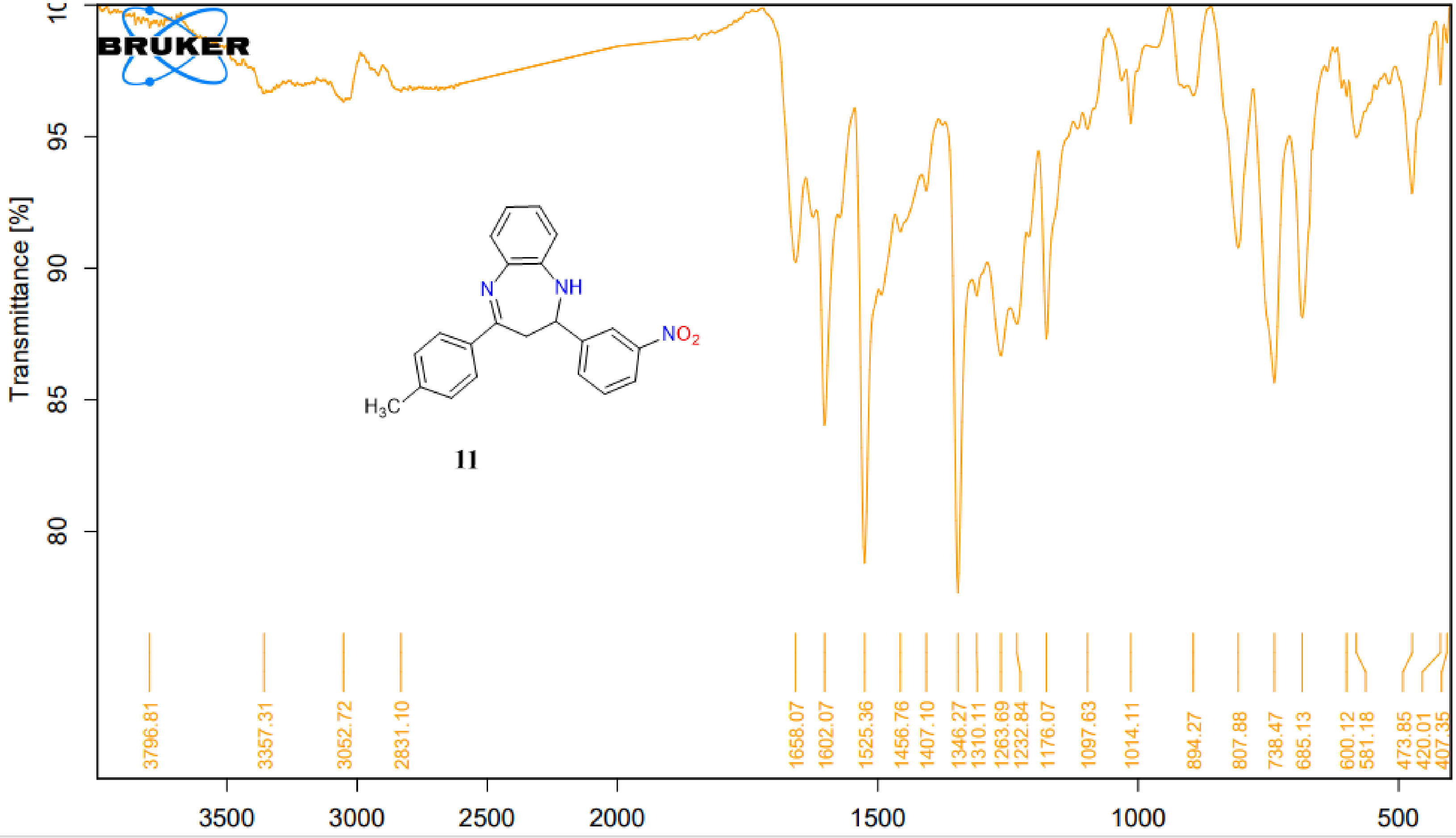

S32: FT-IR spectrum of compound 11 showing characteristic absorption bands at **3357.31 cm⁻¹** (NH) and **1658.07 cm⁻¹** (C=N stretching).

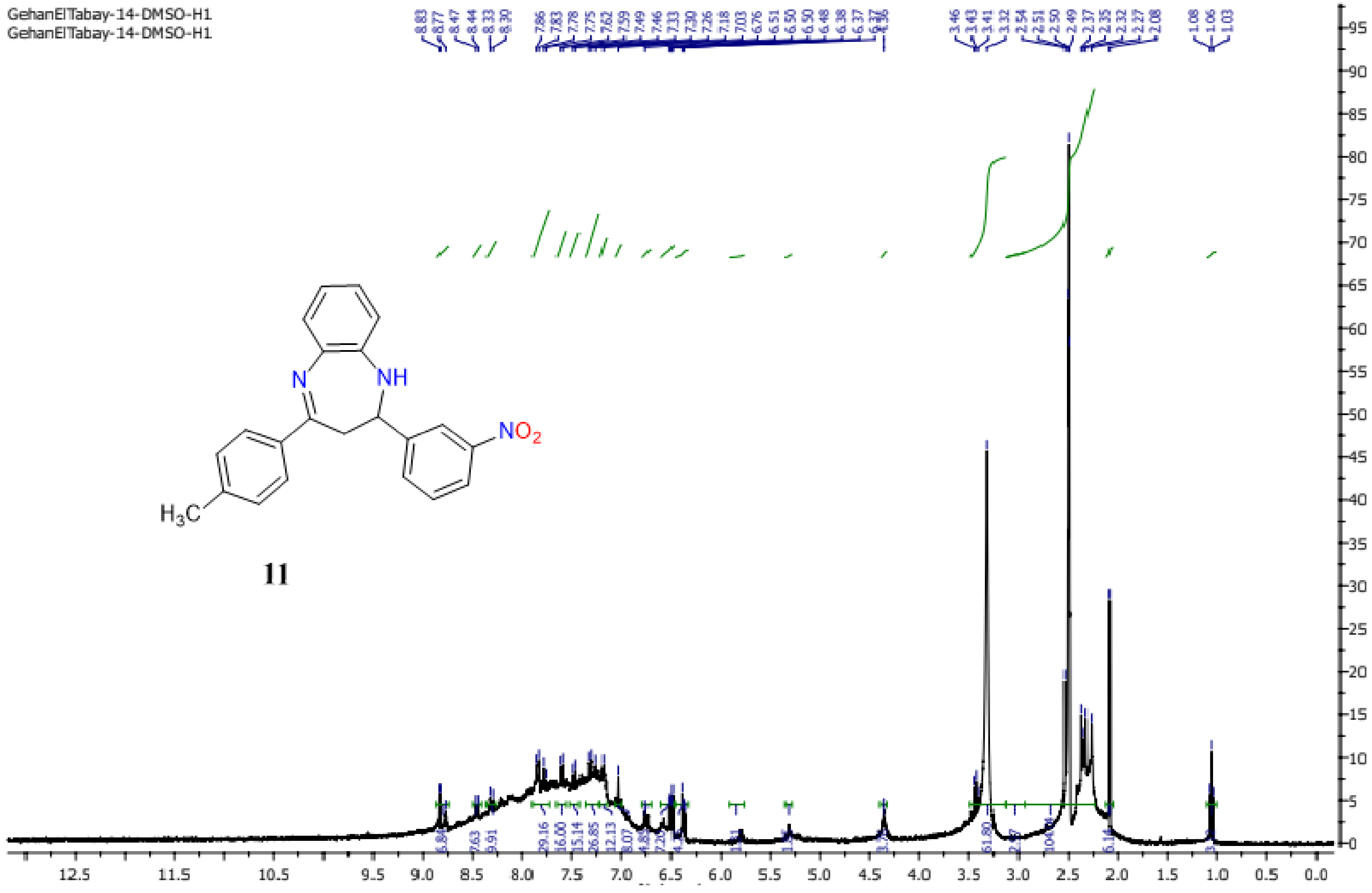

S33: **¹H-NMR Spectrum of Compound 11**. The **¹H-NMR spectrum** of **compound 11** shows a characteristic **singlet at δ 8.83 ppm**, which is assigned to the **NH proton** and **two singlet signals at δ 2.50 ppm and 3.32-3.46 ppm** for CH_3 and CH_2 correspondingly.

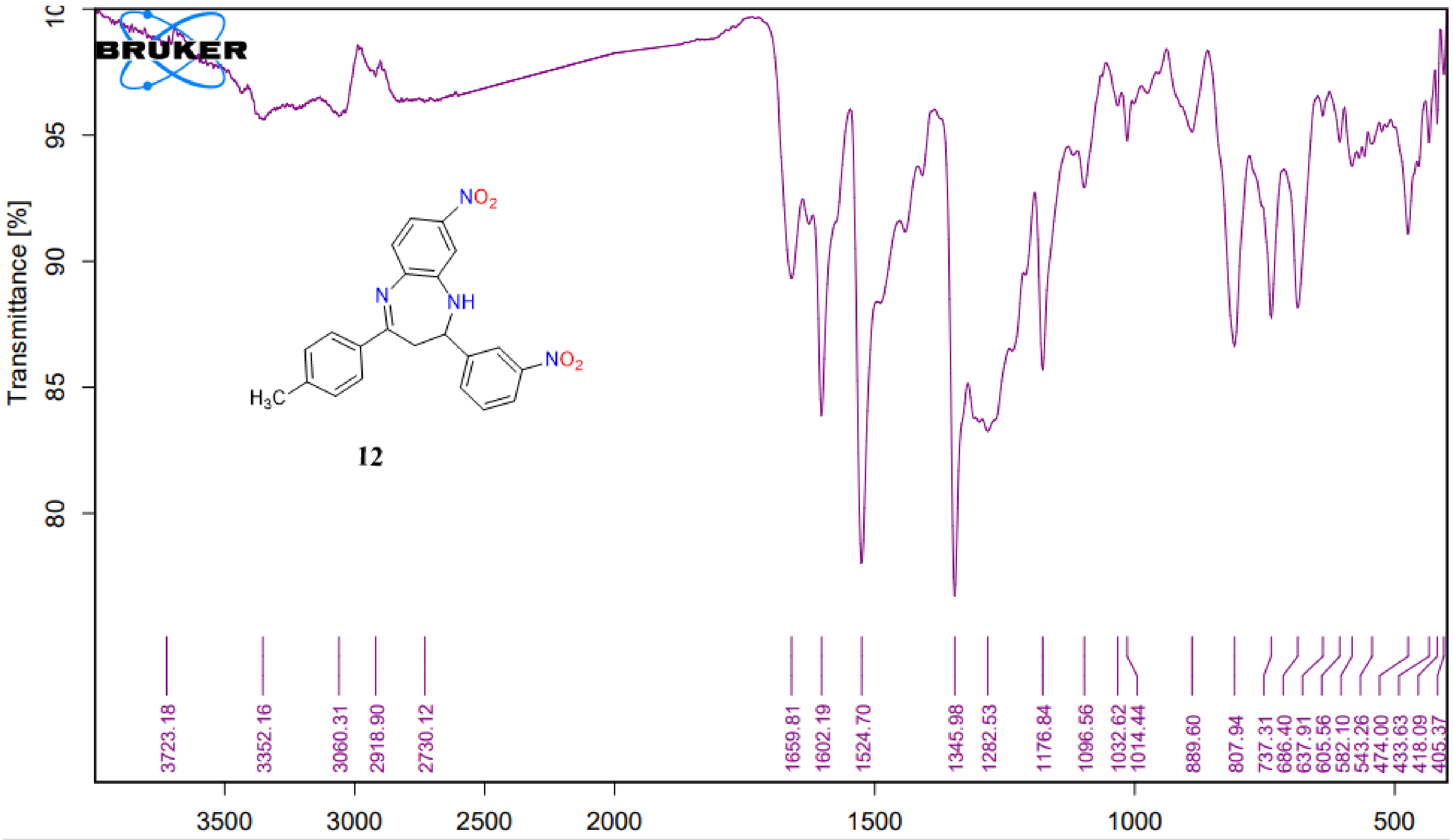

S34: IR Spectrum of Compound 12. The **IR spectrum** of **compound 12** exhibits a strong absorption band at **3352.16 cm^{-1}** for (NH) group and **1659.81 cm^{-1}**, corresponding to the **C=N stretching vibration**.

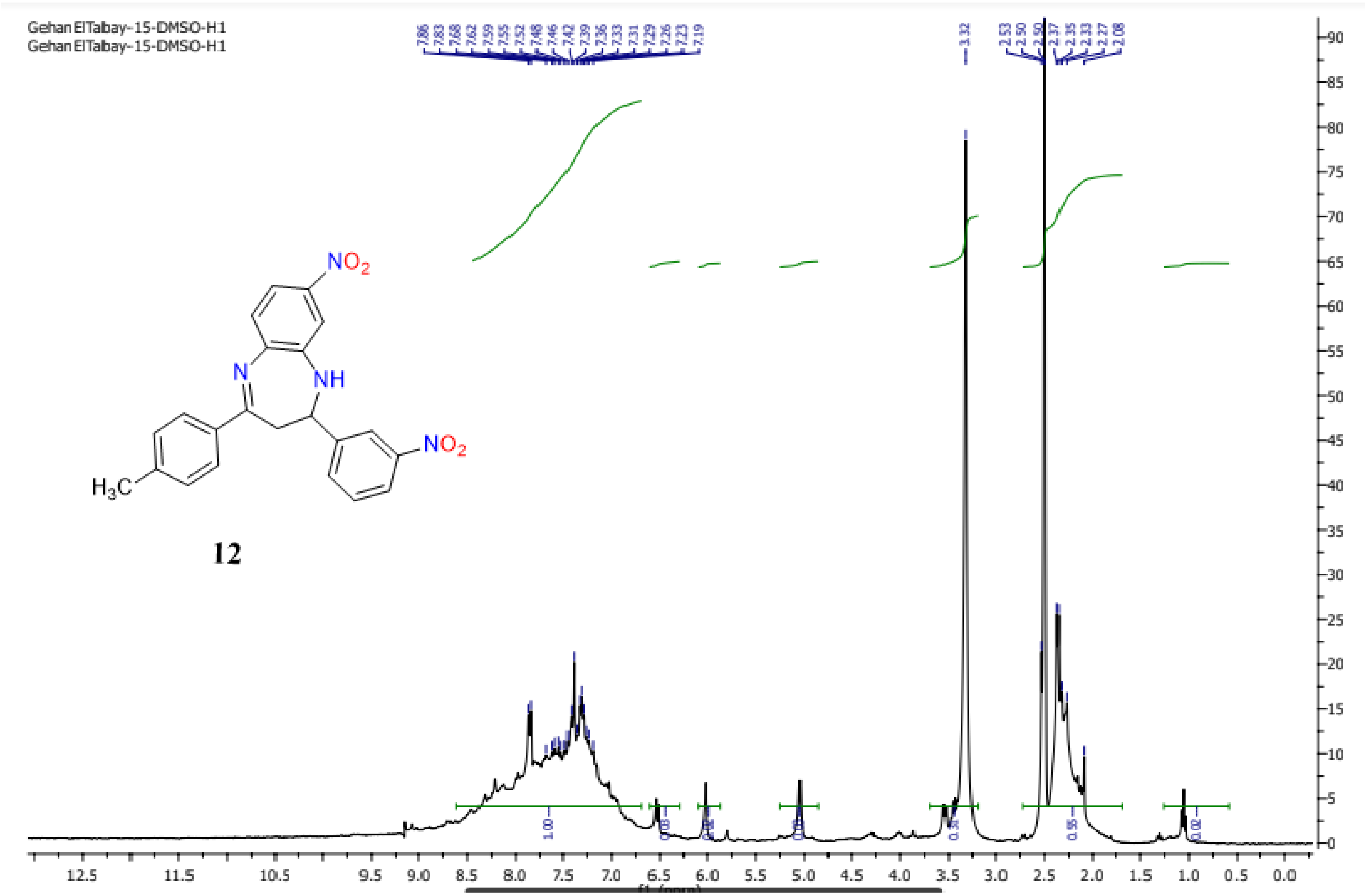

S35: **^{1}H-NMR Spectrum of Compound 12**. The **^{1}H-NMR spectrum** of **compound 12** displays a characteristic **singlet at δ 8.33 ppm**, which is assigned to the **NH proton** and **two singlet signals at δ 2.50 ppm and 3.32-3.46 ppm** for **CH_3** and **CH_2** compatibly.
